# Examining the Impact of Youth Mental Health Services Capacity Growth Trajectories and Digital Interventions on Youth Mental Health Outcomes: System Dynamics Modeling Analysis

**DOI:** 10.2196/71256

**Published:** 2025-08-21

**Authors:** Seyed Hossein Hosseini, Nicholas Ho, Samantha Huntley, Sarah Piper, Paul Crosland, Adam Skinner, Catherine Vacher, Kristen Tran, Kim-Huong Nguyen, Yun Ju Christine Song, Victoria Loblay, Olivia Iannelli, Shahana Ferdousi, Sithum Munasinghe, Sujata Rao, Devin Lam, Zoe Waterhouse-Bushnell, Garner Clancey, Ian B Hickie, Jo-An Occhipinti

**Affiliations:** 1 Youth Mental Health and Technology, Brain and Mind Centre, Faculty of Medicine and Health, Central Clinical School, University of Sydney, 1 King Street, Newtown, 2042, Australia, 61 93510774; 2 Faculty of Medicine, The University of Queensland, Brisbane, Australia; 3 Global Brain Health Institute, Trinity College, Dublin, Ireland; 4 Health Intelligence Unit, Western Sydney Primary Health Network, Westmead, NSW, Australia; 5 Translational Health Research Institute, Western Sydney University, Penrith, NSW, Australia; 6 Law School, University of Sydney, Sydney, Australia

**Keywords:** youth mental health, public health policy, mental health services, system dynamics, Western Sydney

## Abstract

**Background:**

Mental health (MH) issues are the leading cause of mortality for young people, highlighting the importance of timely, high-quality, and affordable care. However, recent trends show a deceleration in the growth of youth mental health (YMH) services capacity in Australia. Meanwhile, digital interventions hold significant potential to sustain and enhance YMH outcomes.

**Objective:**

This study aimed to evaluate (1) the comparative impact of different services capacity growth trajectories on YMH outcomes and (2) whether digital interventions can offset rising demand and declining workforce capacity, to offer insights into strategic resource allocation for sustained improvements.

**Methods:**

Participatory system dynamics modeling was used to investigate the impact of MH services capacity growth trajectories and digital interventions on YMH outcomes, with simulation results projected for 2025‐2035. The study focused on individuals aged 15‐24 years from a culturally diverse, rapidly expanding urban population in Australia. Outcomes assessed included years lived with psychological distress and disorders, MH-related emergency department presentations, and self-harm hospitalizations.

**Results:**

Among the services modeled, doubling the growth rates for specialized MH services had the greatest impact (8.4% reduction in cumulative years lived with symptomatic mental disorder). Doubling the growth rates for specialized MH service, *headspace* (*headspace* National Youth Mental Health Foundation Ltd), and referrals to online services, together, could significantly enhance YMH outcomes. Compared to baseline, this strategic investment approach is projected to reduce cumulative years spent with symptomatic mental disorders, cumulative MH-related emergency department presentations, and cumulative self-harm hospitalizations by 14%, 6.4%, and 4.1%, respectively, from 2025 to 2035. Digital interventions alone produced comparable impacts to specialized services, but critically, could not prevent worsening outcomes when specialized services experienced degrowth. Combining digital interventions with expansion of specialized services yielded best outcomes with reductions of 15%, 5.1%, and 4.4% in these indicators, respectively.

**Conclusions:**

The findings emphasize digital technologies as an effective interim and long-term solution to mitigate the slow and uncertain growth in the specialized MH workforce. However, simulation results showed that achieving sustained long-term improvements necessitates concurrent investment in expanding the specialized MH workforce, as digital interventions alone cannot compensate for degradation in specialized services capacity. A strategic combined approach offers the most effective pathway to improving YMH outcomes.

## Introduction

Mental health (MH) is integral to overall well-being. When individuals do not receive appropriate support and interventions, existing MH issues may deteriorate, increasing the likelihood of developing more severe mental disorders. Poor MH is associated with negative outcomes, including poor academic performance and labor market outcomes, substance misuse, self-harm, and suicide [1,2].

Given their prevalence and significant impact, mental disorders are among the top 10 contributors to the global burden of disease measured in terms of disability-adjusted life years [3]. There has been a dramatic increase in the prevalence of depression and anxiety between 2009 and 2021 in Australians aged between 15 to 34 years, and this experience has been repeated across several high-income countries [4]. In 2022, this number remains high with 49.1% of young people aged 15‐24 years in Australia experiencing moderate to very high levels of psychological distress [5]. Suicide is the leading cause of mortality among young people, with an age-specific death rate due to intentional self-harm of 11.6 per 100,000 individuals aged 15‐24 years in 2022 [6].

Several structural and individual factors are essential to improve MH outcomes, such as providing broad access to high-quality and affordable MH services. Early access to timely, high-quality, and affordable care has the potential to significantly increase recovery rates, thereby reducing waiting times, service disengagement, and MH-related emergency department (ED) presentations, which can subsequently improve the performance of MH care systems [7-9]. Since the average age of onset for mental disorders is 18 years, it is important to ensure that young people have adequate access to the right MH care at the right time [10,11]. However, barriers persist that hinder the effective delivery of such care, including inadequate MH funding, fragmented organization of the health care system, workforce challenges such as shortages of trained professionals, geographical disparities in service availability, and the impact of fluctuating socioeconomic conditions.

In Australia, regional approaches to MH service provision have been recommended to address these systemic barriers more effectively [12]. Regional approaches to MH services are one of the recommendations from a major MH review conducted in 2014 [13], and 31 Primary Health Networks (PHNs) operationally started from July 2015 to locally commission primary MH services [14]. This would provide the opportunity for PHNs to collaborate with different organizations and service providers to commission locally relevant interventions and service pathways by acknowledging sociodemographic profiles, available resources, priorities, and service capacity in the region.

However, the growth rate of youth mental health (YMH) services capacity at the regional level has declined over the past 3‐4 years compared to the trend observed since 2010 [15-17]. If this deceleration of services growth continues, previous gains in YMH service capacity expansion will potentially be lost (see Context section for more details). This emphasizes an urgent need for service reform, particularly through the integration of alternative approaches and digital technologies, such as online MH services and technology-enabled care coordination. In this paper, the term “digital technologies” is used broadly to encompass online MH services and technology-enabled integrated care coordination. Online services range from providing evidence-based information to enhance MH literacy to offering early assessments and cognitive behavioral therapy aimed at preventing mental disorders [18,19]. Additionally, technology-enhanced care has been proposed as a facilitator of more integrated, multidisciplinary care, enabling both consumer and clinician to better track meaningful MH outcomes over time and helping to deliver care based on clinical staging so that treatments are better aligned with a patient’s MH journey [20].

The introduction of these approaches is suggested to improve the efficiency of current YMH services and their capacity, as well as provide more accessible and scalable MH services for young people. Previous research showed that guided and unguided internet-based cognitive behavioral therapies are associated with reduced depressive symptom severity compared to treatment as usual [21]. Maintaining current levels of YMH services capacity necessitates sustained investments to address factors such as infrastructure depreciation and maintenance, workforce dynamics (including retirement, turnover, ongoing training needs, etc), and evolving regulatory requirements [22,23]. These factors pose considerable uncertainties around both health outcomes and budgetary impact, raising debates on the feasibility of major increases in YMH services capacity across all regions in Australia. Accordingly, an interesting question for health services planners and decision makers is the potential role and implications of incorporating digital technologies—an underused tool—in augmenting YMH services. Additionally, the impact of socioeconomic factors on the incidence of MH issues remains uncertain, which complicates projections of future demand for YMH services and the outcomes of such services. Addressing these uncertainties is essential for developing effective strategies to meet the evolving needs of young people.

Therefore, this study aims to answer two key questions: (1) What are the impacts of different YMH services capacity growth rate trajectories (under different scenarios of social determinants) on YMH outcomes? (2) Can digital technologies and services offset the rising demand for MH services and declining workforce capacity? We used the Western Sydney Primary Health Network (WSPHN), a diverse and fast-growing population catchment in Greater Sydney, Australia, as a test case to answer these questions. Moreover, from different YMH outcomes, our focus is on the following items for individuals aged 15 to 24 years: the prevalence of moderate to very high psychological distress with disorder, MH-related ED presentations, and self-harm hospitalizations. To account for the dynamic behavior of these outcomes over time, we will present the cumulative measures as follows: cumulative years spent with symptomatic mental disorders, cumulative ED presentations, and cumulative self-harm hospitalizations.

## Methods

### Context

A regional (PHN-level) system dynamics (SD) model was developed for WSPHN, as part of the Right Care, First Time, Where You Live Program; this initiative aims to strengthen YMH systems by developing innovative decision support tools and enhancing community capacity to leverage systems modeling across 8 sites in Australia [15-17]. The WSPHN catchment covers 4 local government areas: Blacktown, Cumberland, Parramatta, and the Hills Shire and is home to nearly 1.1 million people in 2021. In the 10 years leading up to 2021, Western Sydney’s population grew by more than 20%, which was double the rate of growth for the rest of New South Wales [24]. The area is sociodemographically diverse, with 52.6% of the population having been born outside of Australia and 58.2% not speaking English at home, the highest percentage among all PHNs in Australia [25].

WSPHN has a high youth population, with 33.5% of residents aged younger than 25 years, compared to 30.5% for New South Wales overall. The learning or earning rate in the catchment stands at 84.4%, slightly below the New South Wales rate of 84.9% [26]. The prevalence of any 12-month mental disorder among young people aged 16‐24 years in the WSPHN catchment is 31%, slightly lower than the New South Wales prevalence of 36.3%. Rates of help-seeking in the WSPHN catchment are lower than the New South Wales average rate. In 2023, a total of 19.3% of young people had an MH consultation and 7.9% accessed MH services via digital technologies in the previous 12 months [27]. Additionally, the WSPHN catchment is characterized by lower rates of MH presentations to the ED and self-harm hospitalization for young people below 25 years compared to corresponding rates in New South Wales over the last 5 years. In the first quarter of 2024, nearly 500 per 100,000 young people aged 12‐24 years in the WSPHN catchment presented to ED for MH problems, compared to 720 per 100,000 in New South Wales. Similarly, 119 per 100,000 young people aged 15‐24 years in the WSPHN catchment were admitted to the hospital for self-harm, compared to 187 per 100,000 in New South Wales. Despite these lower rates, the levels of MH-related ED presentations and hospital-presented self-harm have remained stable over the past 5 years in the WSPHN catchment [28].

The WSPHN provides MH support for young people through a range of MH and suicide prevention initiatives including (1) *headspace,* Australia’s National Youth Mental Health Foundation, providing early intervention MH services to those aged 12‐25 years to support young people with MH, physical health (including sexual health), alcohol and other drug services, as well as work and study support [29]; (2) a youth-enhanced support service, which is an outreach program offering MH support for young people aged 12‐25 years who are at risk of developing MH conditions; and (3) child and youth-specific services, which is a part of the primary MH care services [30]. In the 2021 to 2022 financial year, over 83,000 PHN-funded psychological service sessions were provided to residents of Western Sydney, including young people. Additionally, *headspace* and early psychosis services delivered over 10,400 and 21,300 service sessions to 2234 and 641 young people, respectively [25].

**Figure 1.**
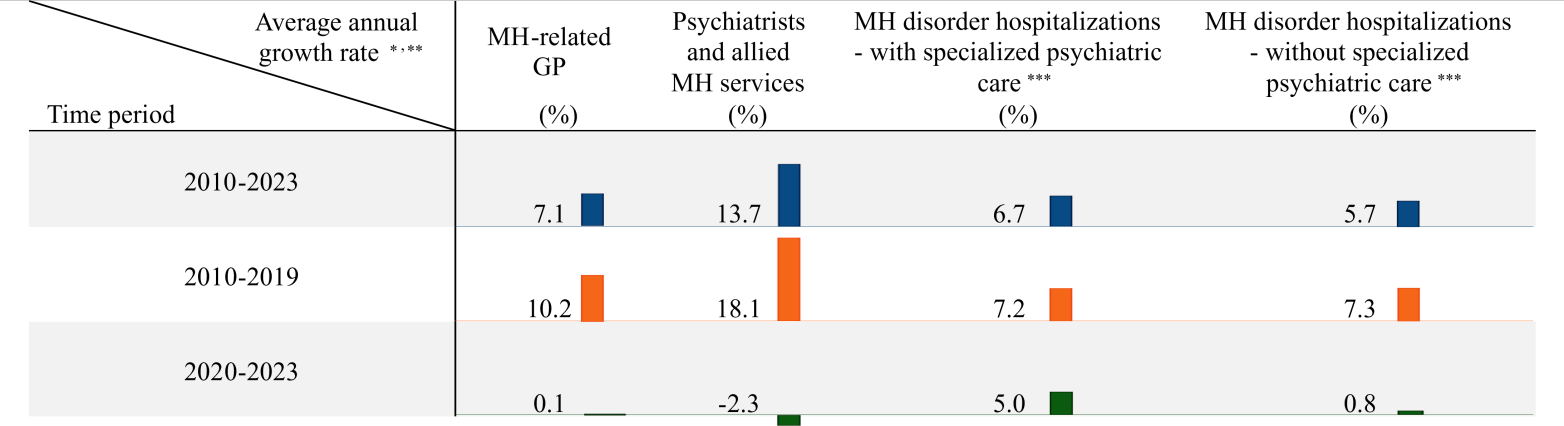
The short-term versus long-term average annual growth rate in YMH services capacity. Data presented here are based on the Australian Institute of Health and Welfare [31], the Centre for Epidemiology and Evidence [32], and the Department of Health [33]. ^*^The state-level data and the data reported at statistical areas were converted to Primary Health Network level using the concordance files provided by the Department of Health and Aged Care [34]. ^**^The data pertains exclusively to individuals aged 15-24 years. ^***^Here, the last data point used for the hospitalizations is 2022. GP: general practitioner; MH: mental health; YMH: youth mental health.

The growth rate of YMH services capacity in the WSPHN catchment has been decelerating in recent years compared to the trend observed since 2010. Figure 1 highlights the difference in near-term growth rates compared to the long-term trend for some MH services. This analysis is based on the data published for the total number of MH-related service contacts for primary care practitioners (henceforth, will be referred to as general practitioners), psychiatrists, and allied MH professionals (including psychologists and MH nurses), as well as mental disorder hospitalizations (with and without specialized psychiatric care). The recent growth rate has been negative or significantly lower than the long-term trend.

Given the expected average yearly population growth rate of 1.42% for individuals aged 15‐24 years (and 1.75% for the overall population) in the WSPHN catchment [35], coupled with the impending exacerbation of the MH workforce shortage due to upcoming retirements (ie, 20% of psychiatrists in Australia are aged older than 50 years, as reported by the Department of Health and Aged Care) [36], the declining rate of growth in service capacity is expected to significantly impact future YMH outcomes. It is therefore critical to examine the impact of different YMH services capacity growth scenarios (trajectories) on YMH outcomes within the WSPHN catchment.

### Model Development Approach

The SD model was technically developed based on the methodology explained by Sterman [37], with steps including problem articulation (boundary selection), developing a dynamic hypothesis, formulating a conceptual and mathematical simulation model, model validation, and policy formulation and evaluation. However, we used a participatory approach that was adopted throughout the modeling process (see Freebairn et al [38] for the details of the modeling approach used). An SD modeling approach with participatory model-building processes was selected as the most appropriate method due to its potential for high-level, strategic, transparent, and accountable decision-making [39,40]. The model development commenced with 3 in-person preliminary site visits for arrangements and planning with WSPHN, followed by 3 in-person workshops engaging over 70 representatives from various stakeholders, including WSPHN, Western Sydney Local Health District (WSLHD), Sydney Children’s Hospitals Network, Department of Communities and Justice, Department of Education, NSW (New South Wales) Ambulance, related nongovernmental organizations, and community MH services (ie, Baabayn Aboriginal Corporation, Youth Off the Streets, Stride, Neami National, and One Door), substance misuse services or programs (ie, Ted Noffs Foundation), tertiary educational and research institutions (The University of Sydney, Western Sydney University, University of Canberra, and Pharmaceutical Society of Australia), and, most importantly, local young people with lived experience of mental ill health and caregivers. In summary, the main agenda for workshop 1 was as follows: introduce systems modeling and the research approach to the stakeholders; collaboratively map determinants of MH and the MH service pathways in the region; and prioritize YMH interventions of interest. In workshop 2, the model structure and logic were presented and discussed, and the interventions were defined by the stakeholders. Subsequently, workshops 1 and 2 provided us with expert knowledge that informed the development of the model and the design of the intervention. The main agenda for workshop 3 was to demonstrate the model and user interface, provide user testing through predefined and user-customized simulation runs, and discuss the key health and economic insights derived from model simulations, which supported the formal simulation analysis by expert knowledge.

Throughout the process, 9 virtual model development group sessions were held with key participants from the workshops and other stakeholders as needed. The number of participants varied across sessions, but each session consistently included representatives from the research team, WSPHN, and young people with lived experience of MH conditions. Other participants primarily comprised stakeholder representatives at the managerial level, who provided insights into data availability, real-world structures to be replicated in the model, and consensus-building on intervention parameters. Throughout these sessions, local experts provided critical insights regarding the shortcomings of available data sources by engaging in detailed discussions on the model structure, underlying assumptions, and the data sources used. These insights informed the parameter ranges used in our baseline model calibration processes, ensuring that our models accurately reflected local conditions despite data limitations. Furthermore, expert advice was essential for determining locally relevant intervention parameters and assumptions, particularly those related to implementation factors such as likely intervention uptake rates. Such parameters were further subject to sensitivity analysis to account for uncertainties. Afterward, 3 training sessions, named super user training, were conducted, involving 25 participants from stakeholder groups which consisted of 1 general training session (focusing on enhancing users’ skills in using the interface to generate insights into YMH outcomes), 1 session focused on economic aspects, and 1 session customized for young people with lived experience of MH conditions that included an additional advocacy component to support the use of the model.

The model was continuously refined throughout the workshops, model development group sessions, and training sessions based on the feedback received from the ongoing engagement of the experts and participants representing various stakeholders. The model structure and parameter values were strongly supported by an evidence base extracted from the literature, available regional, state, and national time series data, and expert opinion. Additionally, constrained optimization [41] was used to estimate parameter values in cases where evidence or data were lacking (see Multimedia Appendix 1 for the main parameter values extracted from the literature and the data sources). Model construction, interface design, and simulation analysis were performed using Stella Architect (version 3.0.1 [42]).

### Ethical Considerations

This study has been approved by the Human Research Ethics Committee of the Sydney Local Health District (X21-0151 and 2021/ETH00553) and was conducted per the NHMRC (National Health and Medical Research Council) National Statement on Ethical Conduct in Human Research (NHMRC 2007, updated 2023). The study used only aggregate-level secondary data sources. Stakeholder participation in the model co-design workshops was completely voluntary, and they were not the subject of the research. Where relevant, travel and meal costs were reimbursed to participants.

### Model Overview

The model includes 13 subsystems as detailed below:

1. The population subsystem captures population dynamics through births, migration (both overseas and interstate), aging, and mortality. The population is segmented into the following age groups: 0‐4, 5‐11, 12‐14, 15‐17, 18‐24, and 25 years and older.
2. The education subsystem models students enrolled in primary, secondary, and postsecondary education. It also tracks the number of people with secondary and postsecondary qualifications.
3. The labor force subsystem models the flow of people from sufficiently employed, underemployed, unemployed, and not-in-the-labor force statuses in 2 age groups: 15‐24 years and 25 years and older. It includes unemployment, underemployment, and participation rates.
4. The NEET (Not in Employment, Education or Training) subsystem models the population aged 15‐24 years who are NEET.
5. The strengths and difficulties subsystem models the effects of early life exposures for children aged 0‐4 years and 5‐11 years on the flows of people to and from states of close to average, slightly raised, and high or very high risk of emotional and behavioral problems as measured using the Strengths and Difficulties Questionnaire [43].
6. The psychological distress or disorder subsystem models the flows of people to and from states of low psychological distress (K10 score 10‐15) [44], moderate to very high psychological distress (K10 score 16 and above but not meeting the criteria for 12-month psychological disorder) and moderate to very high psychological distress (K10 score 16 and above) meeting the criteria for 12-month psychological disorder; the prevalence of each state is calculated in this subsystem.
7. The substance misuse subsystem models the prevalence of 12-month substance misuse disorder and substance misuse closed treatment episodes.
8. The homelessness subsystem models the population experiencing homelessness.
9. The justice subsystem models the flow of young people into and out of the justice system for people aged 12 and older.
10. The social cohesion subsystem models population-level social cohesion according to the Scanlon-Monash Index of Social Cohesion [45].
11. The family and domestic violence subsystem models exposure to family and domestic violence across all age groups.
12. The suicidal behaviors subsystem models the rates of suicide attempts (using self-harm hospitalizations as a proxy) and suicide deaths for people aged 12 years and older with different distress levels.
13. The services subsystem models the flow of psychologically distressed individuals through various service pathways, including general practitioners, specialized services (including MH services provided by psychiatrists and allied MH professionals such as psychologists, social workers, and occupational therapists), EDs, psychiatric and nonspecialized hospitalizations, community-based MH services (including Child and Youth Mental Health Services and community mental health care), *headspace* services, and online services. Henceforth, the term “all services” will refer collectively to the MH services outlined in this paragraph.

**Figure 2.**
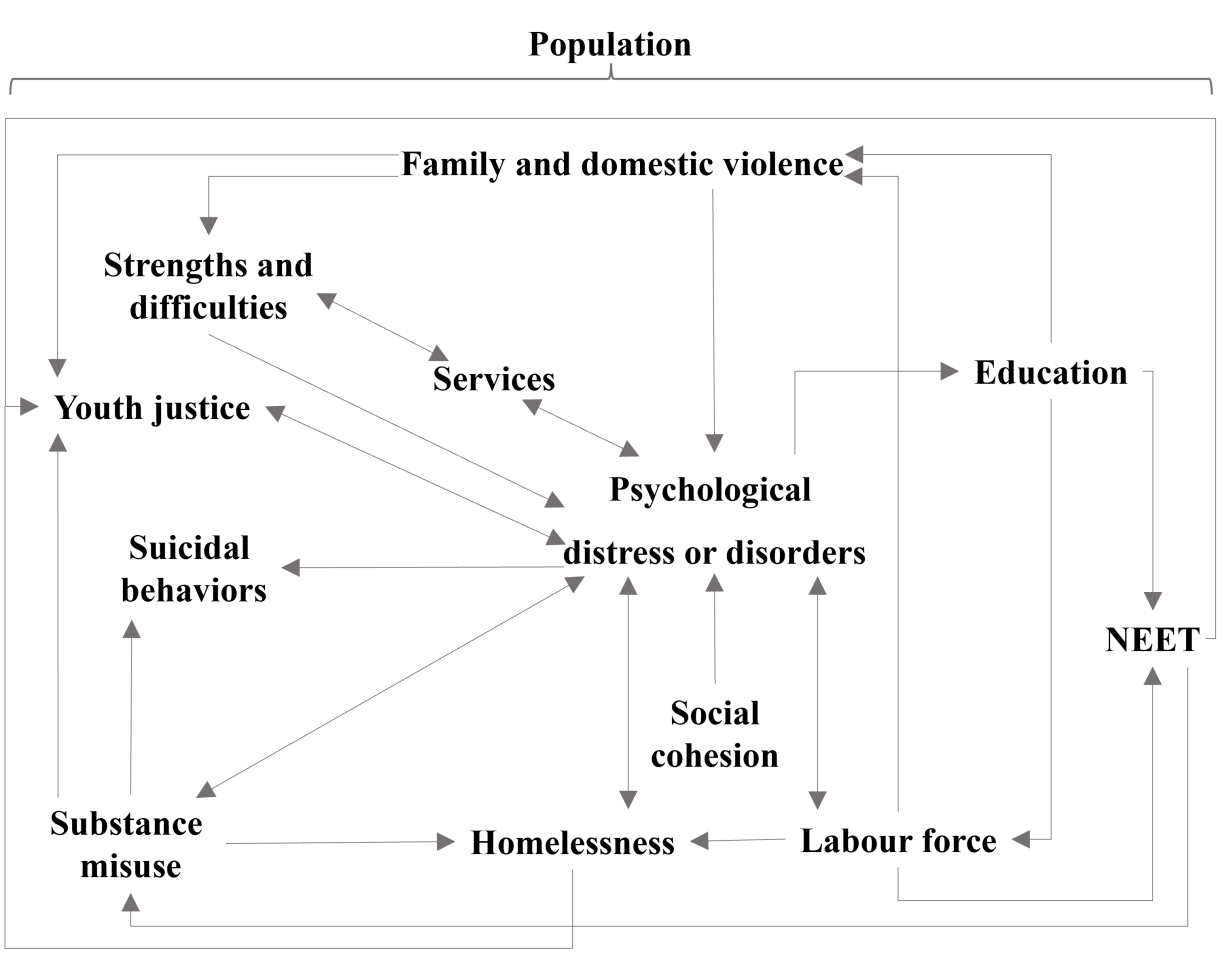
Overview of the subsystems and causal structure of the WSPHN system dynamics model. NEET: Not in Employment, Education or Training; WSPHN: Western Sydney Primary Health Network.

Figure 2 provides a high-level overview of the causal structure of the model in which arrows represent how subsystems of the model affect other parts of the model; single-headed arrows indicate unidirectional causal connections; bidirectional causal connections are shown as double-headed arrows.

More details about the main model structure, including the structure of each subsystem and its interactions with other subsystems, data sources, and evidence from previous studies used in each subsystem, as well as model validation, are provided in Multimedia Appendix 1.

### Simulation Analyses

Three main analyses were conducted in this research. To answer the research question (1), we examined the impact of various YMH services’ capacity growth trajectories to find the scenario that offers the greatest improvement in YMH outcomes. Specifically, we aim to identify which capacity increase will most effectively improve YMH outcomes, providing valuable insights for investment decisions regarding YMH service capacity growth. This necessitated 5 scenarios that were simulated and then compared to the baseline:

- Doubling growth rate of services capacity (all services)
- Doubling growth rate of specialized services (alone)
- Doubling rate of referrals to online services (alone)
- Doubling growth rate of *headspace* services (alone)
- Doubling growth rate of specialized, online, and *headspace* services (combined)

In the first simulation, starting in 2025, the capacity growth rate for all YMH services was doubled, based on the historical trend from the past 3‐4 years used in the baseline (see Multimedia Appendix 1 for the main parameter values). The model assumes that the recent 3‐4 year trend in service capacity growth rates will continue into the future. Consequently, any changes introduced in these scenarios are applied relative to this baseline trend. The subsequent simulations followed a similar setup.

To address the research question (2), we extended the initial analysis by factoring in constraints on YMH service capacity growth in the short term, primarily due to budget limitations and workforce shortages. This analysis investigates whether increased referrals to online MH services, alongside the implementation of technology-enabled integrated care coordination, can effectively mitigate these challenges. To answer this, we tested 4 scenarios based on the most impactful services identified in the first analysis and compared them to the baseline, as follows:

- Doubling growth rate of specialized services (alone)
- Doubling referrals to online services plus technology-enabled integrated care
- Degrowth in specialized services even with digital interventions in place
- Digital interventions plus doubling growth rate of specialized services from 2028

Similar to the simulation setup in the previous analysis, the first scenario doubles the specialized services capacity growth rate from 2025 based on the historical trend from the past 3‐4 years used in the baseline. The next scenario simulates digital interventions, including [1] the doubling of referrals to online services and implementing technology-enabled integrated care coordination. The third scenario explores whether digital interventions alone can improve YMH outcomes when there is negative growth in specialized services capacity (ie, a 3-fold reduction in the historic growth rate in services capacity). To reflect the real-world barriers to immediately increasing the specialized capacity growth (eg, staff hiring, training, etc) and to better understand the implications for short-term YMH outcomes, the final scenario applies the 2 digital interventions while delaying the doubling of specialized services capacity growth by 3 years (starting in January 2028). This scenario accounts for how policy cycles, budget constraints, and other real-world factors such as workforce training pipelines could delay the expansion of services.

Finally, the third analysis accounts for uncertainties in both the implementation of digital interventions and the social determinants of YMH through 2 separate sensitivity analyses. Here, 2 key sources of uncertainty were considered. The first relates to uncertainties in the implementation of digital interventions. The primary factors include the uptake rate of technology-enabled integrated care and the size of its effects on recovery, disengagement from MH services, and the rate of referrals to specialized care. Additionally, uncertainties about the recovery rate and treatment duration for online MH services are considered. The second set of uncertainties relates to the social determinants of YMH. In this analysis, we examine the impact of variations in historical trends for factors such as social cohesion, the onset of substance misuse, and adverse early life exposures.

Table 1 outlines the setup for the 2 sensitivity analyses. The Latin hypercube sampling method [46] was used to generate 200 random combinations of the listed parameters via Lognormal and Random Uniform distributions, depending on the nature of the data and available evidence. The selection and fitting of these distributions were based on standard practices for representing uncertainty in similar policy modeling studies [47]. For the probabilistic sensitivity analysis of digital interventions implementation, parameter variability was informed by 3 key data sources. First, for parameters with available evidence from the literature, we extracted confidence or uncertainty intervals (UIs) from published meta-analyses, systematic reviews, or randomized controlled trials, as detailed in Table 1. Next, the sensitivity analysis addresses the uncertainties in key social determinants of YMH (ie, social cohesion, substance misuse, and adverse early life exposures as indicated by scores on Strengths and Difficulties Questionnaire administered to children aged younger than 12 years), with parameter ranges derived from the maximum observed variability in the historical data. Lastly, for parameters informed by expert opinion (ie, the implementation uptake rate of the “technology-enabled integrated care” intervention), we used expert estimates to define the UIs. Both sensitivity analyses started in 2025.

**Table 1.** Setup of the 2 sensitivity analyses.

Parameters	Baseline value	Random distribution setup		Details
		Distribution	Parameters	
**Uncertainties in the implementation of technology-enabled integrated care and online mental health services**				
Technology-enabled integrated care uptake rate	50%	Random uniform	Lower bound=30% and upper bound=70%	The lower and upper bounds were determined based on expert opinions obtained during the participatory modeling process.
Effect size of technology-enabled integrated care on recovery	1.177	Lognormal	μ=0.16324; σ=0.24061	Values were derived from Woltmann et al [48], a meta-analysis of the effect of collaborative chronic care models on global MH^a^. Cohen d and the related 95% CI were used to calculate the odds ratio (as the baseline value), μ, and σ.
Effect size of technology-enabled integrated care on disengagement	0.72	Lognormal	μ=−0.3285; σ=0.12506	Values were based on Campbell et al [49], which examined the effect of internet-delivered treatment on retention in treatment. The mean hazard ratio was used as the baseline value, with the associated 95% CI used to calculate μ and σ.
Effect size of technology-enabled care on referrals to specialized care (medium to very high distress, with disorder)	1.266	Lognormal	μ=0.23579; σ=0.40718	Values were derived from Badamgarav et al [50], a systematic review of the effectiveness of disease management programs in referrals to specialized care. Cohen d and its 95% CI were used to calculate the odds ratio (as the baseline value), μ, and σ.
Online services recovery rate (medium to very high distress, without disorder)	0.4	Lognormal	μ=−0.91629; σ=0.28552	Values were sourced from Christensen et al [18], which analyzed the effectiveness of delivering interventions for depression by using the internet. The average odds ratio across different services was used as the baseline value, with the 95% CIs applied to determine μ and σ. The effect size ratio of primary care to specialized care was set at 0.462687, based on Cuijpers et al [51]
Online services recovery rate (medium to very high distress, with disorder)	0.18507	Lognormal	μ=−1.687; σ=0.28552	Values were sourced from Christensen et al [18], which analyzed the effectiveness of delivering interventions for depression by using the internet. The average odds ratio across different services was used as the baseline value, with the 95% CIs applied to determine μ and σ. The effect size ratio of primary care to specialized care was set at 0.462687, based on Cuijpers et al [51]
Mean treatment duration for online services (weeks)	6	Random uniform	Lower bound=2 and upper bound=10	We used the mean, minimum, and maximum duration of the online services mentioned in Zhou et al [52]—a systematic review of the effectiveness of online MH interventions for young people. Notably, 6 points (out of a total of 37 studies) with durations exceeding 10 weeks were excluded as outliers.
**Uncertainties in (selected factors from) social determinants of YMH** ^**b**^				
Change in the historical trend of social cohesion (from 2025)	0	Random uniform	Lower bound =−14% and upper bound=7%	The maximum and minimum change rate ratio of the historical data on social cohesion measured by the Scanlon-Monash Index [53] were used to define the lower and upper bounds of the random uniform distribution. To account for unforeseen uncertainties, these bounds were widened by approximately 50%.
Change in the historical trend of substance misuse onset rate (from 2025)	0	Random uniform	Lower bound =−10% and upper bound=0%	The maximum and minimum change rate ratio of the historical data on individuals with 12-month substance use disorder [27] were used to define the lower and upper bounds of the random uniform distribution. To account for unforeseen uncertainties, these bounds were widened by approximately 50%.
Change in the historical onset of raised SDQ^c^ scores (from 2025)	0	Random uniform	Lower bound =−20% and upper bound=16%	The maximum and minimum change rate ratio of the historical data on the prevalence of different SDQ states and the population in different SDQ levels [54] were used to define the lower and upper bounds of the random uniform distribution. To account for unforeseen uncertainties, these bounds were widened by approximately 50%.

a MH: mental health.

b The parameters in this section of the table were applied as yearly change rate multipliers, augmenting the baseline change rate derived from historical trends.

c SDQ: Strengths and Difficulties Questionnaire.

## Results

### YMH Outcomes in Western Sydney With Different Capacity Growth Trajectories

**Figure 3.**
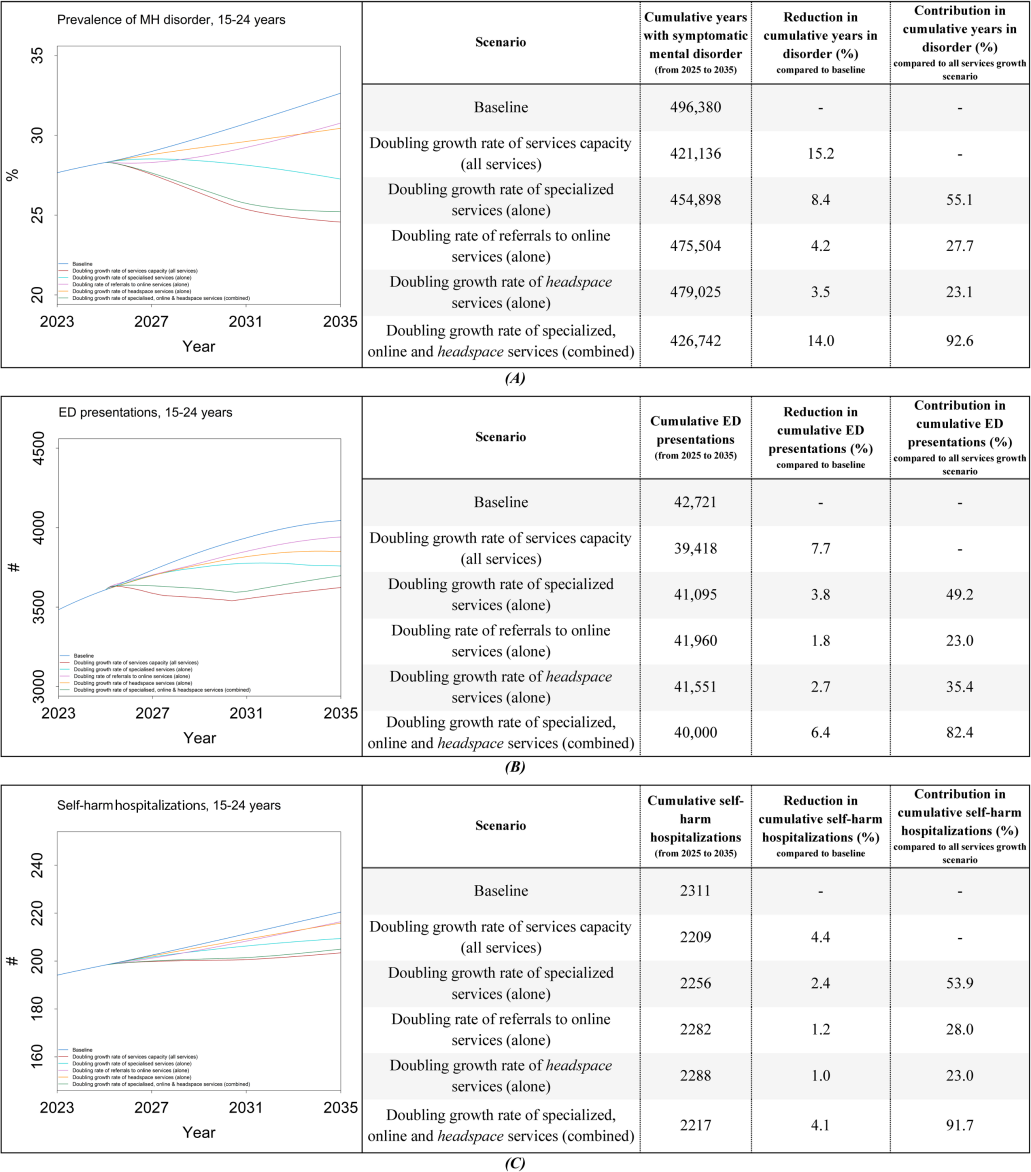
Simulation results under different YMH services capacity growth rates for (**A**) prevalence of mental disorder, (**B**) ED presentations, and (**C**) self-harm hospitalizations (for individuals aged 15‐24 years). ED: emergency department; MH: mental health; YMH: youth mental health.

The first scenario shows that doubling the capacity growth rate for all YMH services leads to reductions in the prevalence of mental disorder, MH-related ED presentations, and self-harm hospitalizations for individuals aged 15‐24 years compared to the baseline by 2035 (Figure 3). Specifically, results for the indicators accumulated from 2025 to 2035 show a 15.2% reduction in the cumulative years spent with symptomatic mental disorders (cumulative years of being in the state of moderate to very high psychological distress with disorder), a 7.7% decrease in cumulative MH-related ED presentations, and a 4.4% decline in cumulative self-harm hospitalizations. When comparing the impacts of doubling services capacity individually, as seen in scenarios 2 to 4, specialized services were the most impactful. Doubling the capacity growth rate for *headspace* services had an impact comparable to that of doubling referrals to online services. The fifth scenario shows that when the capacity growth rate doubled for specialized services, *headspace,* and referrals to online services, they accounted for a large proportion of the impacts of doubling all services capacity growth rates (contributing 92.6%, 82.4%, and 91.7% to the total impact, respectively, across the 3 population MH outcomes).

### Impact of Digital Interventions

**Figure 4.**
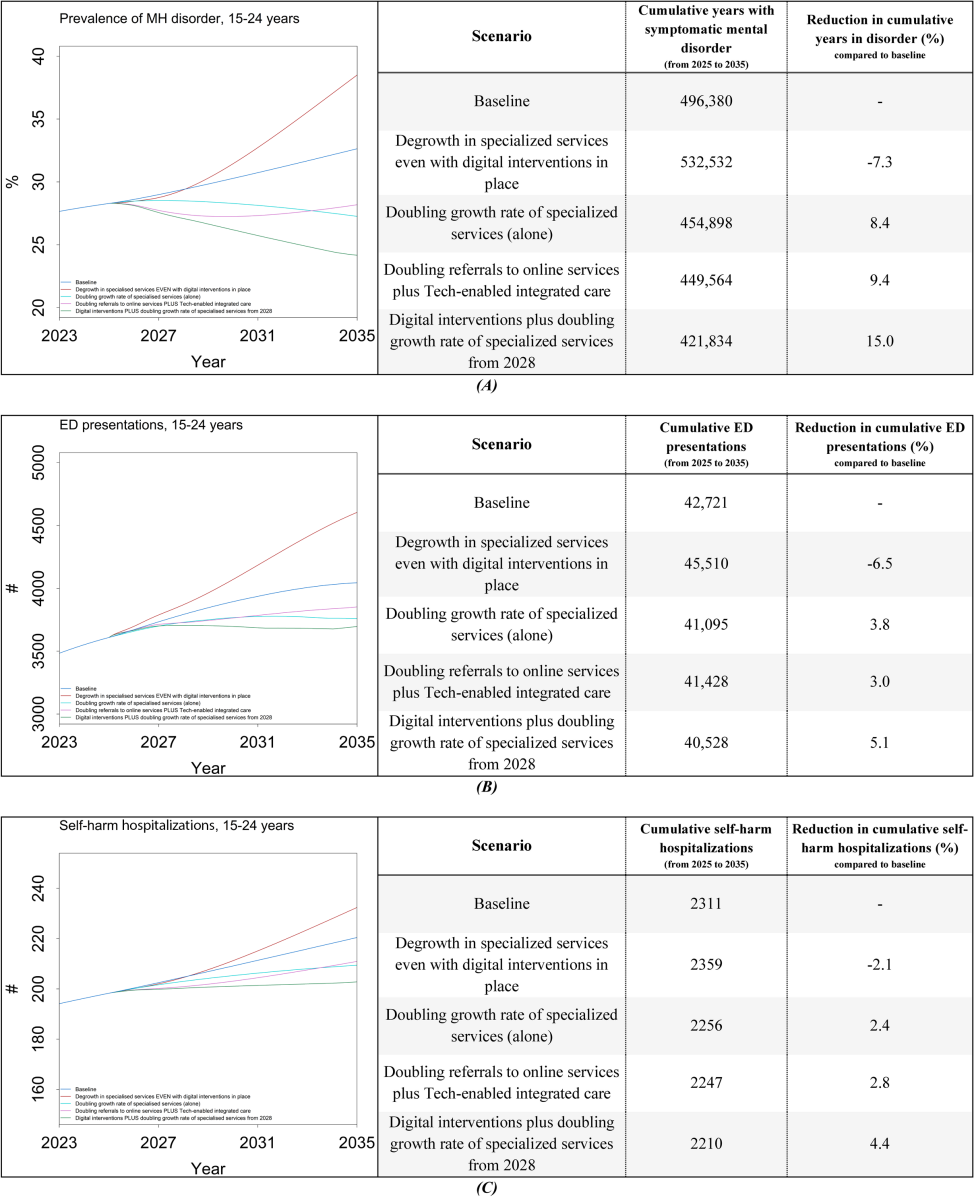
Simulation results under different implementation scenarios of digital interventions for (**A**) prevalence of mental disorder, (**B**) ED presentations, and (**C**) self-harm hospitalizations (for individuals aged 15‐24 years). ED: emergency department; MH: mental health.

As shown in Figure 4, the simulation results for the degrowth scenario showed that without continued investment in maintaining and expanding specialized services capacity, digital interventions alone are insufficient to improve YMH outcomes; instead, significant increases are observed in the prevalence of mental disorders, MH-related ED presentations, and self-harm hospitalizations for youths aged 15‐24 years.

Additionally, the results for the cumulative indicators showed that doubling the growth rate of specialized services alone has a similar impact compared to implementing digital interventions alone. The final (combined) scenario demonstrates a slightly less-than-additive effect, leading to reductions of 15% in the cumulative years spent with symptomatic mental disorders, 5.1% in the cumulative MH-related ED presentations, and 4.4% in the cumulative self-harm hospitalizations, compared to the baseline.

### Sensitivity Analysis to Capture Uncertainty

**Figure 5.**
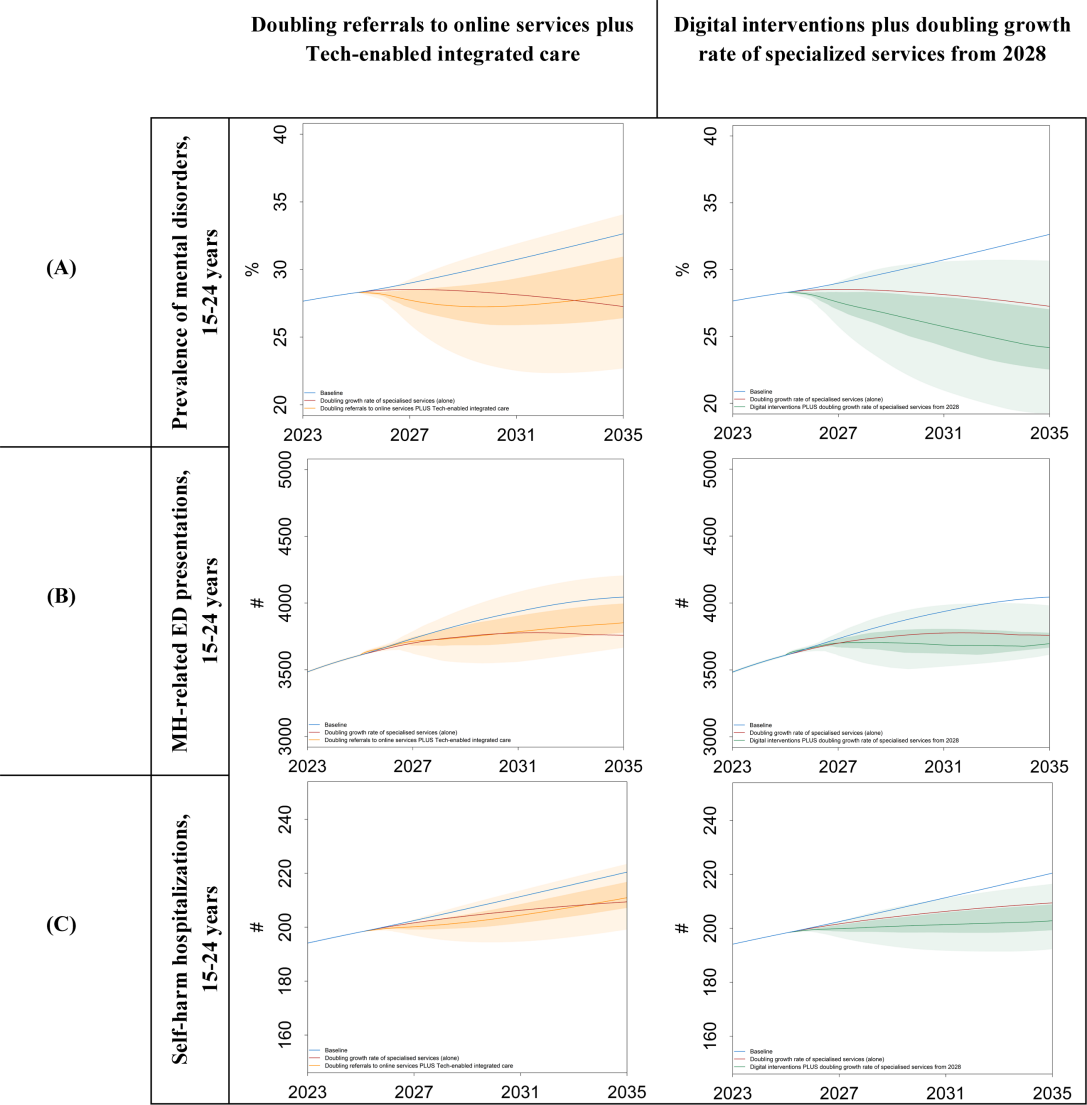
Results of sensitivity analysis for uncertainties in the implementation of digital interventions for (**A**) prevalence of mental disorder, (**B**) ED presentations, and (**C**) self-harm hospitalizations (for individuals aged 15‐24 years). ED: emergency department; MH: mental health.

The graphical results of the first sensitivity analysis are presented in Figure 5. The solid lines represent the scenario outcomes using the default parameter values, with darker shading indicating 50% UIs and lighter shading showing 95% UI. For “doubling referrals to online services plus technology-enabled integrated care,” the probability that it results in a reduction in the prevalence of MH disorders, MH-related ED presentations, and self-harm hospitalizations compared with the baseline scenario is somewhere between 50% and 95%. We are less certain whether it achieves better outcomes than doubling the growth rate of specialized services (alone) because the estimated outcomes for all 3 measures remain within the bounds of the 50% UI at nearly all time points.

When the digital interventions are combined with doubling the growth rate of specialized services, there is at least a 95% probability that all 3 outcomes are better than baseline by the end of the time horizon. There is at least a 50% probability that the prevalence of MH disorders and self-harm hospitalizations is better compared with doubling the growth rate of specialized services (alone), but we are less certain of this for ED presentations.

**Figure 6.**
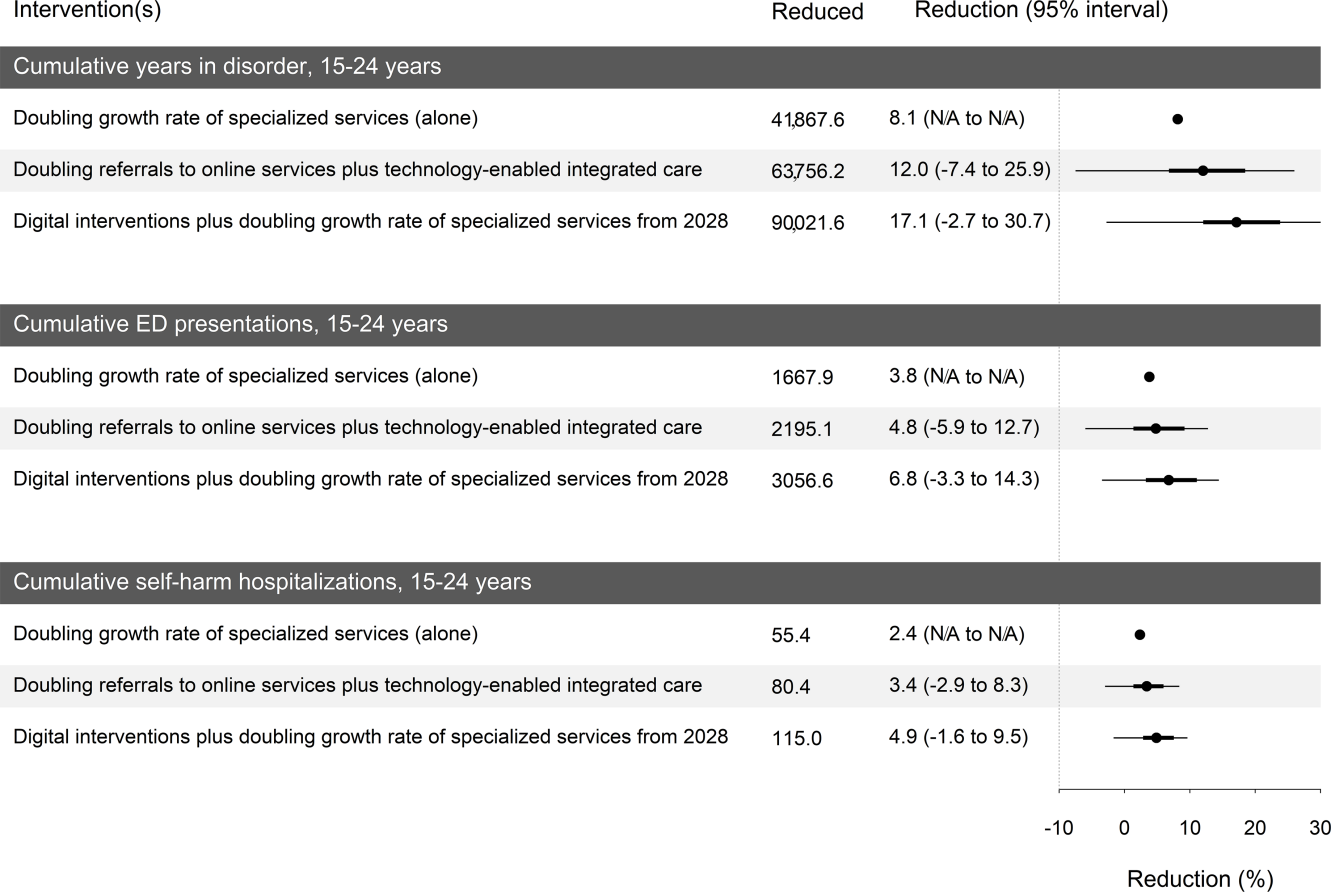
Summary of sensitivity analysis results for uncertainties in the implementation of digital interventions. 95% UI is not reported for the first scenario, as no sensitivity analysis was conducted due to the absence of a technology component in this scenario. ED: emergency department; N/A: not applicable; UI: uncertainty interval.

Figure 6 summarizes the results for the cumulative indicators compared to the baseline (accumulated from 2025 to 2035). For instance, the average reductions in the cumulative years spent with symptomatic mental disorders are 8.1%, 12% (95% UI −7.4% to 25.9%), and 17.1% (95% UI −2.7% to 30.7%) for the scenarios “doubling the growth rate of specialized services (alone),” “doubling referrals to online services plus technology-enabled integrated care,” and “digital interventions plus doubling the growth rate of specialized services starting in 2028,” respectively. The average reductions for the cumulative MH-related ED presentations and cumulative self-harm hospitalizations ranged from 3.8% to 6.8% and 2.4% to 4.9%, respectively.

**Figure 7.**
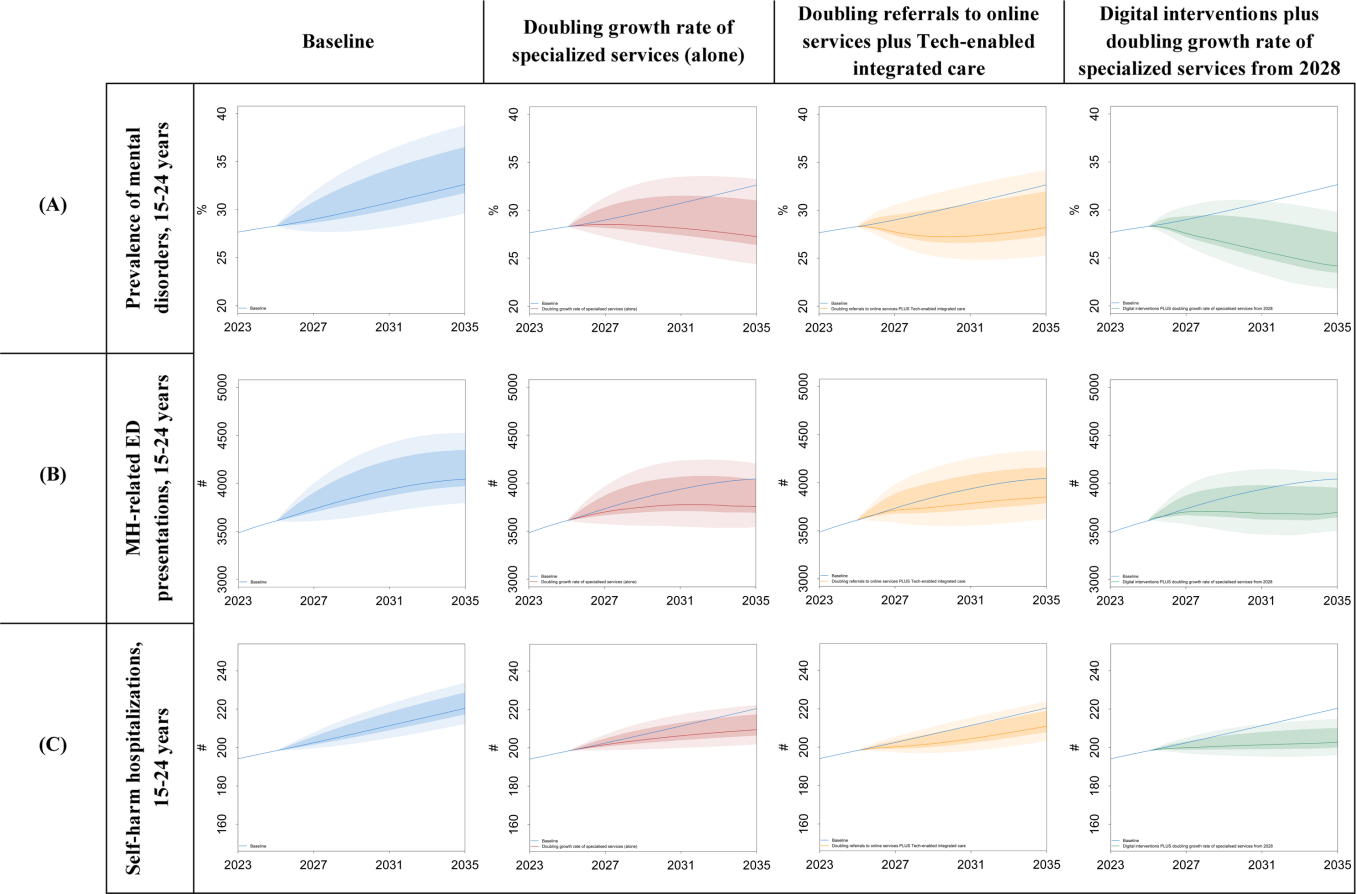
Results of sensitivity analysis for uncertainties in the social determinants of YMH for (**A**) prevalence of mental disorder, (**B**) ED presentations, and (**C**) self-harm hospitalizations (for individuals aged 15‐24 years). ED: emergency department; MH: mental health; YMH: youth mental health.

The results of the second sensitivity analysis, as presented in Figure 7, show a narrower range of variation compared to the first analysis. Self-harm hospitalizations exhibit less response to uncertainties in the selected social determinants of YMH, while the prevalence of mental disorders and ED presentations demonstrates a higher sensitivity to these uncertainties. For “digital interventions plus doubling growth rate of specialized services from 2028,” there is at least a 95% probability that self-harm hospitalizations and the prevalence of mental disorders will be improved compared to the baseline by the end of the time horizon, even when uncertainties in the broader social determinants of YMH are considered. However, for MH-related ED presentations, the probability of achieving better outcomes under similar conditions is at least 50%.

**Figure 8.**
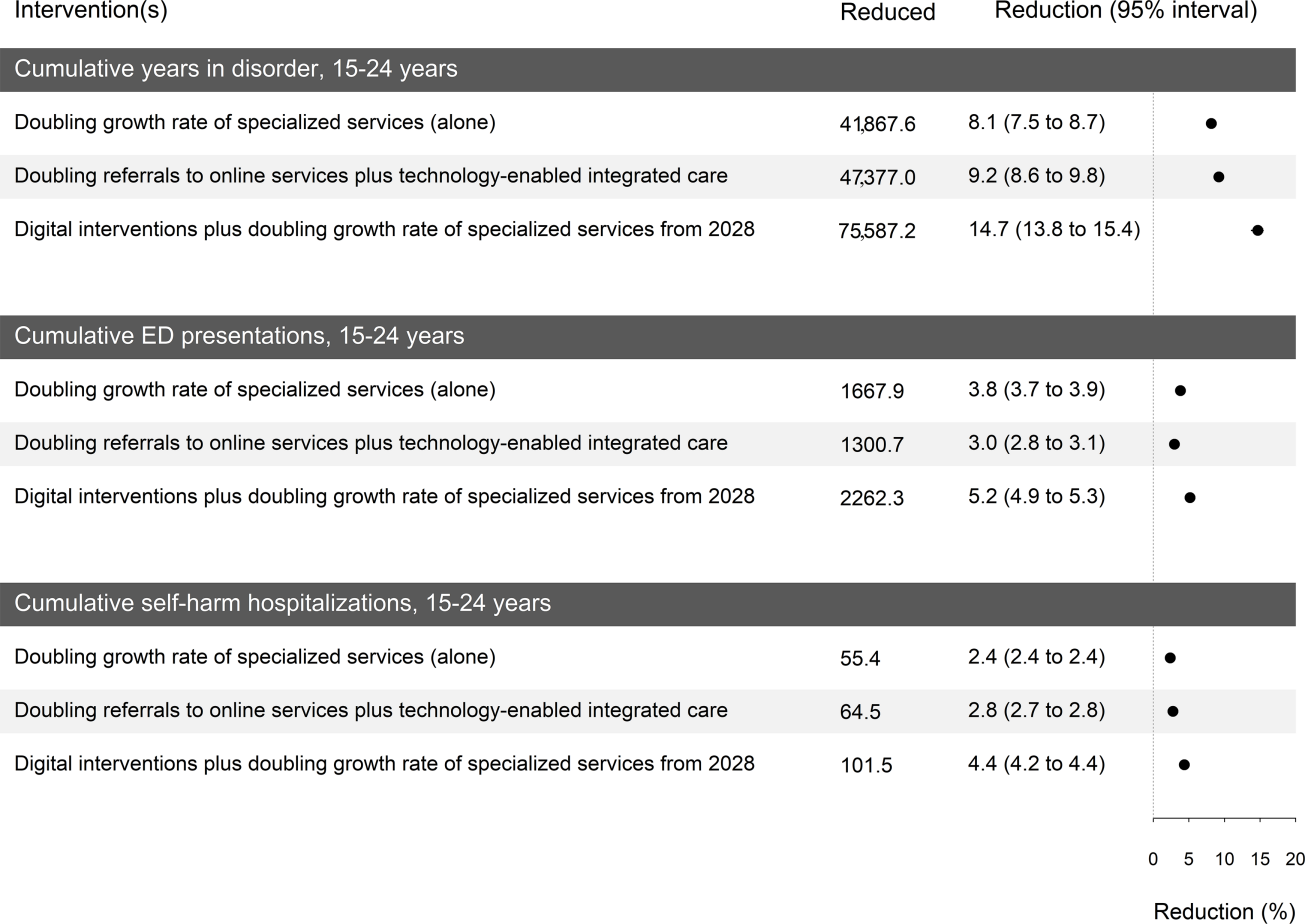
Summary of sensitivity analysis results for uncertainties in the social determinants of YMH. The 95% UI ranges are not distinctly visible in the graphical form due to their narrow range. ED: emergency department; UI: uncertainty interval; YMH: youth mental health.

Figure 8 summarizes the results for the cumulative indicators compared to the baseline. For instance, the average reductions in the cumulative years spent with symptomatic mental disorders are 8.1% (95% UI 7.5% to 8.7%), 9.2% (95% UI 8.6% to 9.8%), and 14.7% (95% UI 13.8% to 15.4%) for the scenarios “doubling the growth rate of specialized services (alone),” “doubling referrals to online services plus technology-enabled integrated care,” and “digital interventions plus doubling the growth rate of specialized services starting in 2028,” respectively. The average reductions for the cumulative ED presentations and the cumulative self-harm hospitalizations ranged from 3% to 5.2% and 2.4% to 4.4%, respectively.

## Discussion

### Principal Findings

This study used SD modeling to simulate the impact of different MH services capacity growth trajectories and the role of digital interventions on YMH outcomes within the WSPHN catchment.

The simulation results demonstrated a deteriorating trend in the prevalence of mental disorder, MH-related ED presentations, and self-harm hospitalizations under a baseline, business-as-usual scenario, and how different trajectories in YMH services capacity growth are projected to variably impact population MH outcomes for young people. Key findings indicate that a combination of increasing capacity growth rates in specialized services, *headspace*, and increasing referral rates to online services was projected to provide the greatest impact on population-level YMH outcomes. This emphasizes the importance of timely expansion of access to specialized [7,8] and online [18,21] services for YMH. These results, however, do not diminish the value of other services that play important roles in the care system, particularly for specific subpopulations. Additionally, the simulation results clearly highlight the potential system-wide impacts of digital interventions, aligning with findings from previous studies that have demonstrated their efficacy and with calls for them to support integrated MH care [18,20,21]. Digital interventions offer a valuable interim measure while longer-term investments are made to expand the specialized workforce. Our modeling also showed that while specialized MH services had the greatest individual impact, the combined strategy with digital interventions yielded substantially better outcomes across all outcomes, providing valuable guidance for strategic resource allocation decisions.

This study’s findings extend the existing international evidence base regarding digital MH interventions for youth populations. Our projections on digital intervention efficacy align with the World Health Organization’s Mental Health Action Plan (2013‐2030), which emphasizes digital technologies as a strategic approach to address global service gaps [55]. The projected impacts of integrated approaches combining digital and specialized services are consistent with the recent Lancet Psychiatry Commission on YMH [56], which advocates for multimodal service models across diverse health care systems. Our findings also further substantiate a systematic review by Garrido et al [57], which identified that while digital interventions for YMH demonstrate efficacy, their optimal implementation requires systematic integration with existing service structures—congruent with our finding that digital interventions cannot independently compensate for specialized service degradation.

While it might be easy to conclude that specialized services growth is unnecessary if we implement digital solutions, the MH workforce shortage and population growth in the WSPHN catchment, as discussed in the Context section, along with the simulation results for the “degrowth in specialized services capacity” scenario, suggest otherwise. It was outlined in the Results section (Impact of Digital Interventions) that even with digital interventions in place, both specialized services and digital solutions are needed to stabilize and subsequently improve YMH outcomes, emphasizing the critical role of timely access to specialized services [8]. Our finding that digital interventions alone could not prevent worsening outcomes when specialized services experienced degrowth underscores the limitations of a digital-only approach, regardless of its implementation quality.

The shortage of a multidisciplinary workforce, including both clinical and nonclinical professionals, has been identified as a significant barrier to reforming youth primary MH care toward an integrated model [56]. Workforce constraints are a global challenge, as evidenced by World Health Organization data indicating a projected shortfall of 18 million health workers by 2030, including critical MH professionals [58]. The density of MH workers remains critically low worldwide, with significant disparities between countries. Low-income countries have a median of 1.4 per 100,000 population, while lower-middle-income countries have it at 3.8 per 100,000 [59]. This contrasts sharply with high-income countries, which have a median of 62.2 per 100,000 [59]. While our findings are specific to the Australian context, the SD modeling approach used in this study offers a methodological framework that could be adapted to examine similar questions about digital interventions and service capacity in other health care systems facing comparable challenges. The increasing global demand for YMH services, compounded by growing populations, highlights the urgent need to address gaps in the specialized workforce. As recently underscored by the *Lancet Psychiatry Commission* on YMH [56], integrated digital interventions and blended models of care hold considerable potential to drive innovation, facilitate system reform, and enhance the overall effectiveness of YMH services. Our findings further demonstrate that while digital interventions can play a critical role in addressing short-term shortages in specialized services, sustained long-term improvements require concurrent investment in expanding the specialized MH workforce. This dual approach ensures that the gains achieved through digital interventions are maintained and integrated into a robust and effective YMH care system.

Finally, the sensitivity analysis results revealed a relatively wide range of model reactions to the uncertainties in the implementation of technology-enabled integrated care and online MH services. This highlights both the potential benefits and the critical importance of the effective implementation of the interventions related to digital technologies targeted at young people. Additionally, the sensitivity analysis results showed that the uncertainties in (selected factors from) social determinants of YMH, including social cohesion [45], substance misuse [2], and adverse early life exposures [43], should not be overlooked, as they can significantly influence the effectiveness and success of new interventions.

### Perspectives of Youth Lived Experience

As discussed in the Methods section, we used a participatory systems modeling approach, engaging various stakeholders throughout the entire process, from defining the problem to summarizing key insights. Here, we present the summarized perspectives of 2 young people with lived experience of MH conditions on the results discussed above (Textbox 1).

Textbox 1. **Perspectives of youth lived experience.**

We participated in all workshops and super user training, with DL as part of the model development group. The participatory systems modeling approach lets us, as young people, help create a more targeted model by sharing the real-life challenges we and others in our region face. While the model is a representation, it has been shaped by some of the experiences of young people with lived experience in an attempt to maximize its accuracy. The co-designed process provided a safe, collaborative space where our voices were heard. Having other stakeholders, like service planners, attend the workshops meant that young people could use their perspectives to increase the knowledge of service planners about youth mental health treatment. This direct line of communication between consumer and provider was invaluable, and it is our hope that the service planners incorporate our feedback in future service provision. Our ongoing role in the national reference group will ensure transparency and accountability so this evidence informs real-world interventions, not just academia.

Reducing the prevalence of mental health disorders is the most critical outcome. This should logically lower emergency department presentations, self-harm hospitalizations, and other negative outcomes. Prevention and early intervention would make mental health concerns easier to treat and reduce young people's distress.

We understand that addressing mental health needs requires a multifaceted approach. While doubling the growth rate of all services is effective, it’s unrealistic. Digital interventions could serve as a first contact for young people, some of whom prefer them over face-to-face care. However, technology alone isn’t the solution. Doubling specialized services' growth rate from 2028, combined with digital interventions, seems most feasible given financial and efficacy considerations.

Our personal experiences highlight the need for evidence-based, effective improvements in youth mental health outcomes amidst declining trust in institutions. Ultimately, progress depends on the responses of institutions and communities. The model isn’t perfect—for instance, challenges in schools aren’t included in definitions of young people NEET (Not in Employment, Education or Training). This program also underscores the need for further research, like exploring how peer workers might influence outcomes.

DL and ZW-B served as youth lived experience advisors in the Right Care, First Time, Where You Live program for this region. They are now members of both the local reference group in Western Sydney and the National Youth Reference Group for the program. The National Youth Reference Group brings together youth champions from the 8 sites across Australia, facilitating shared learnings across regions participating in the program. The formation of this network has supported the youth champions in enhancing systemic cooperation within the YMH system (Loblay et al, unpublished data, 2025).

### Limitations

This study does not assess the impact of various interventions on YMH outcomes from health or economic perspectives (for example, see Crosland et al [60]). Instead, it focuses solely on YMH outcomes under different trajectories of YMH services capacity growth. Building on the research presented here, further analysis could be conducted to identify the optimal combination of interventions to enhance YMH outcomes within the WSPHN catchment (see Occhipinti et al [61] for an example). Additionally, further investigation is required to assess whether budget constraints on intervention implementation, as well as uncertainties in the broader social determinants of YMH, might alter the optimal solution.

Moreover, we acknowledge potential limitations in the quality and accuracy of secondary data sources used to parameterize the model, despite using strategies such as triangulation of multiple data sources, parameter estimation via constrained optimization, and local verification to identify plausible estimates.

### Conclusions

This study used an SD simulation model to explore how different trajectories of YMH service growth can impact outcomes within the WSPHN catchment area. The findings indicate that a strategic combination of investment in specialized, *headspace*, and online services can significantly enhance population-level YMH outcomes. Importantly, this research highlights the essential role of digital technologies as a viable interim solution while investments are being made to expand the specialized workforce over the longer term.

However, it is essential to recognize that the effectiveness of digital interventions is maximized when integrated with increasing specialized services capacity; both components are necessary to effectively and sustainably improve population-level YMH outcomes.

The results of sensitivity analysis highlighted both the potential benefits and the critical importance of effectively implementing interventions based on digital technologies. They also emphasized the significance of uncertainties in social determinants for long-term planning to improve YMH.

## Supplementary material

10.2196/71256 Multimedia Appendix 1 Model structure, modeling approach for services capacity growth rate, and numerical inputs.

## Abbreviations

ED: emergency department
MH: mental health
NEET: Not in Employment, Education or Training
NHMRC: National Health and Medical Research Council
PHN: Primary Health Network
SD: system dynamics
SDQ: Strengths and Difficulties Questionnaire
UI: uncertainty interval
WSPHN: Western Sydney Primary Health Network
YMH: youth mental health
