# Supplementary material for: Examining the Impact of Youth Mental Health Services Capacity Growth Trajectories and Digital Interventions on Youth Mental Health Outcomes: System Dynamics Modeling Analysis
Source: J Med Internet Res. 2025 Aug 21;27:e71256. doi: 10.2196/71256 (PMC12375514; doi:10.2196/71256)
Supplement: Multimedia Appendix 1 [file jmir-v27-e71256-s001.docx]

**Contents**

Model structure 2

Overview 2

Population 2

Education (students) 4

Education (highest level of qualification) 6

Labour force 8

Not in employment nor education (NEET) 10

Psychological distress / disorder 11

Strengths and Difficulties 13

Homelessness 14

Family & domestic violence (FDV) 15

Youth justice 16

Substance misuse (substance misuse disorder) 17

Substance misuse (substance misuse closed treatment episodes) 18

Suicidal behaviours 19

Social cohesion 20

General Practitioner (GP) 21

Specialist services 22

Community mental health care services 23

headspace 25

Online mental health services 26

Mental health related emergency department (ED) presentations 27

Psychiatric admitted care 28

Non-specialised admitted care 29

Help-seeking and disengaged 30

Modelling approach for services capacity growth rate 31

Numerical inputs 32

References 43

# **Model structure**

## **Overview**

The system dynamics model reflects the Western Sydney Primary Health Network (WSPHN) catchment and consists of multiple sectors that represents different aspects of the population.

The sectors of the model are explained in section 2.3 and the overview of the model is depicted in Figure 1 in form of a subsystems diagram in the primary paper entitled “Examining the Impact of Youth Mental Health Services Capacity Growth Trajectories and Digital Interventions on Youth Mental Health Outcomes”. Following, Figures S1 to S23 present the structure of each sector. Please note that for each stock, an inflow representing the suicide deaths prevented through various interventions is included in the model but have been left out of these figures for visual clarity. For further details on each sector, please contact the corresponding author.

Data used to calibrate each sector are described in figure legends. For any data extracted at the Statistical Area (SA) level of geographic granularity (such as those from the Australian Bureau of Statistics (ABS)), we concorded these data to Primary Health Network (PHN) level estimates through concordance files supplied by the Australian Department of Health and Aged Care [1].

## **Population**

The population sector models the estimated resident population of the WSPHN catchment into six age-specific stocks. The stocks correspond to people aged 0-4 years, 5-11 years, 12-14 years, 15-17 years, 18-24 years, and 25 years and older. Each stock has a mortality outflow and a net migration biflow. Births flow into the stock of 0-4-year-olds and people follow an ageing chain flowing from the younger to older age stocks. This sector is calibrated with data based on estimated resident births, deaths, and population data from HealthStats at NSW Ministry of Health [2–4] and the Public Health Information Development Unit (PHIDU) at Torrens University Australia [5], and migration [6–8] statistics from the ABS.

**Figure S1.** Structure of the population sector.


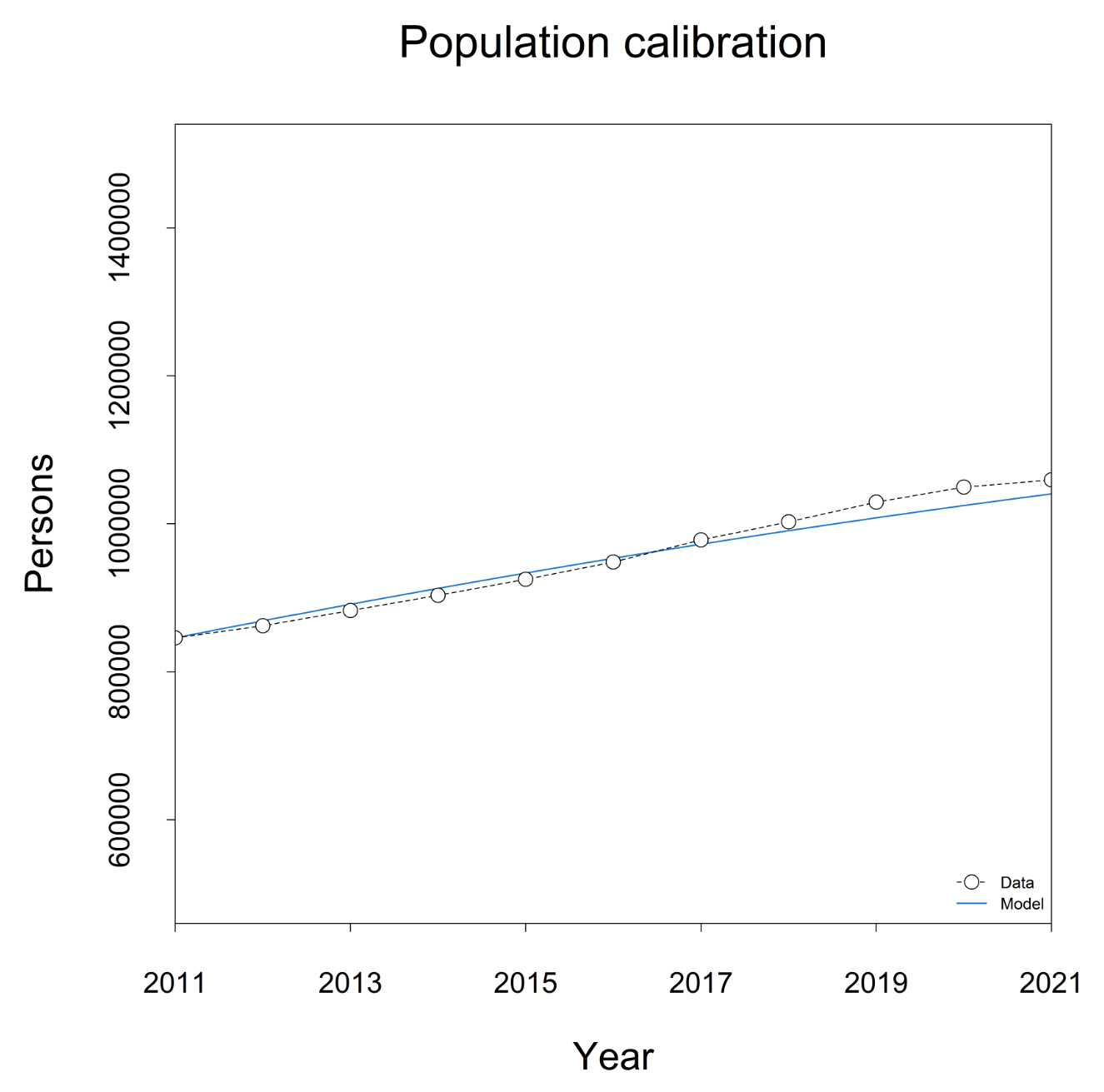

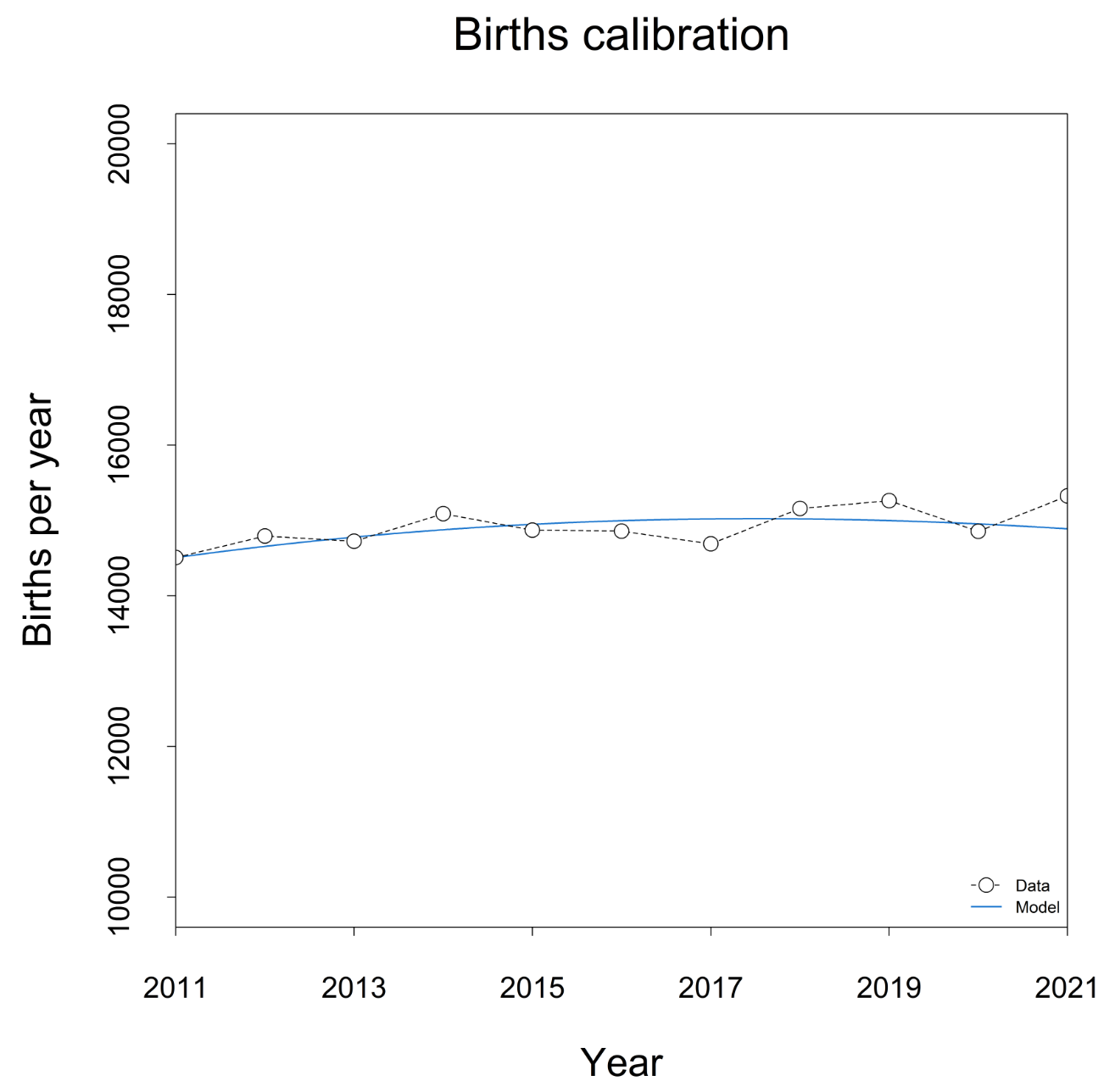

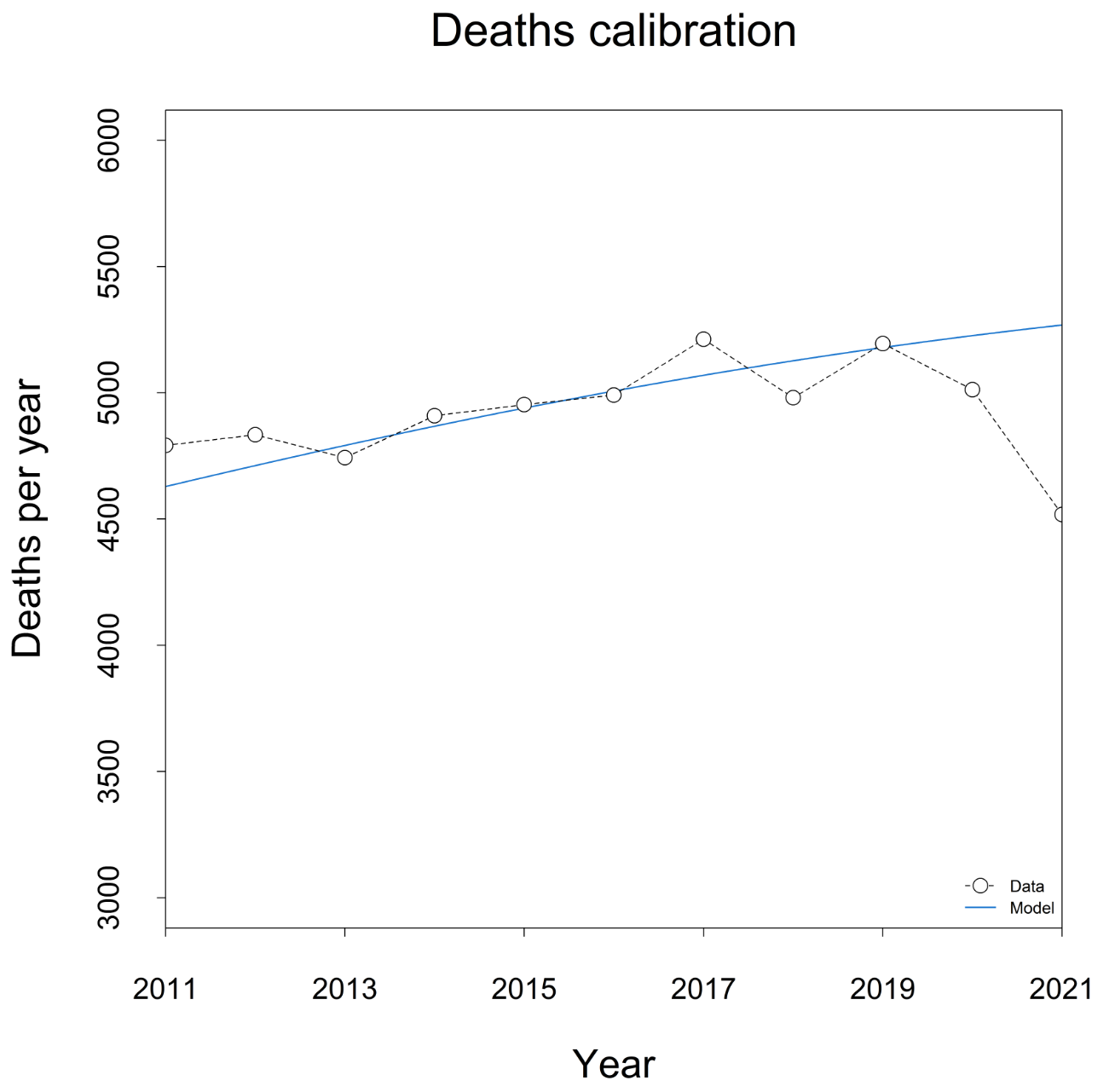


**Figure S2.** Calibration plots from the population sector.

## **Education (students)**

The education (students) sector models students enrolled in education in WSPHN. The stocks correspond to students enrolled in primary education, secondary education and post-secondary education. Each stock has a mortality outflow and a net migration biflow. People flow into the “Studying primary education” stock as they age from 4 to 5 years of age. Graduates of primary education then transition to secondary education, and graduates of secondary education can either transition to post-secondary education or not (e.g. those commencing employment). Students in secondary or post-secondary education may discontinue their studies at rates dependent on the prevalence of psychological distress / disorder [9,10]. People may enter secondary or post-secondary studies without directly transitioning from primary education or secondary education respectively (e.g. re-enrolling after discontinuation, people enrolled in both secondary and post-secondary studies). This sector is calibrated using student enrolment [11] and completion [12] data from the Australian Curriculum Assessment and Reporting Authority (ACARA), post-secondary education destinations data from the NSW Department of Education [13], and education and work statistics from the ABS [14].


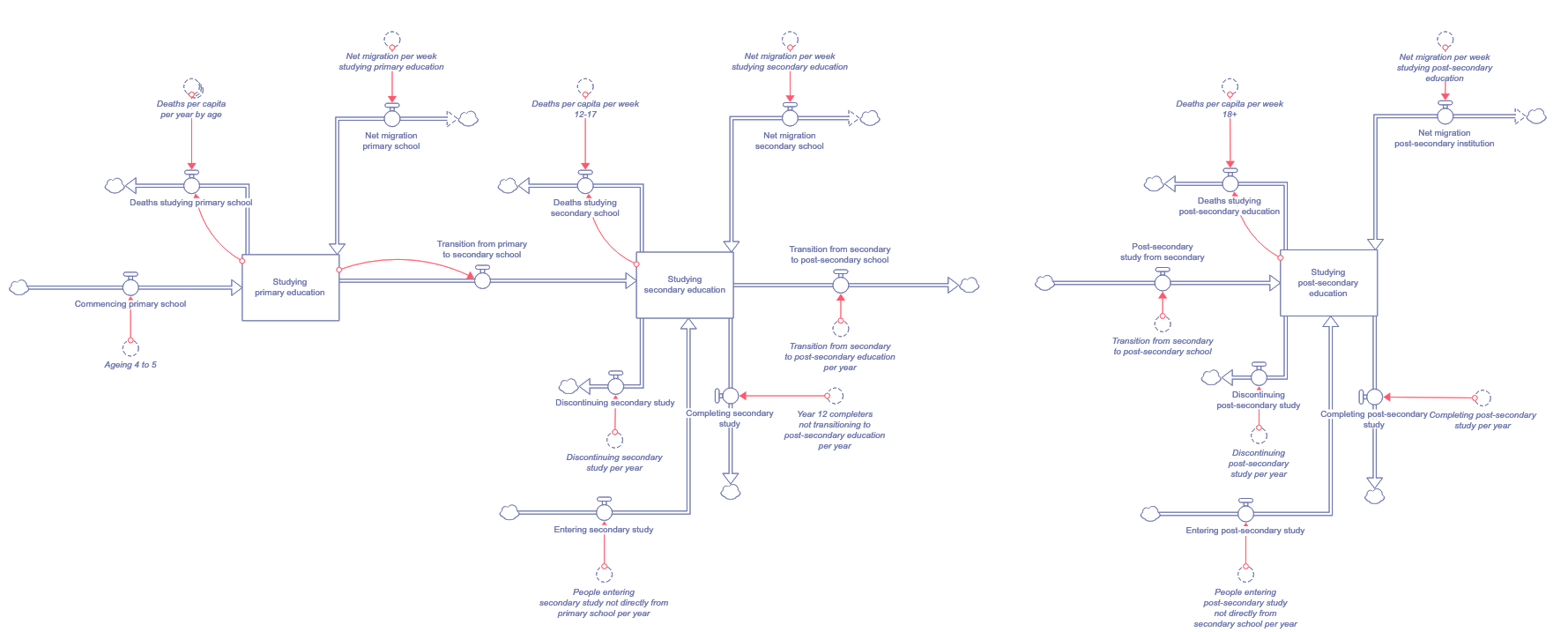


**Figure S3.** Structure of the education (students) sector.

**
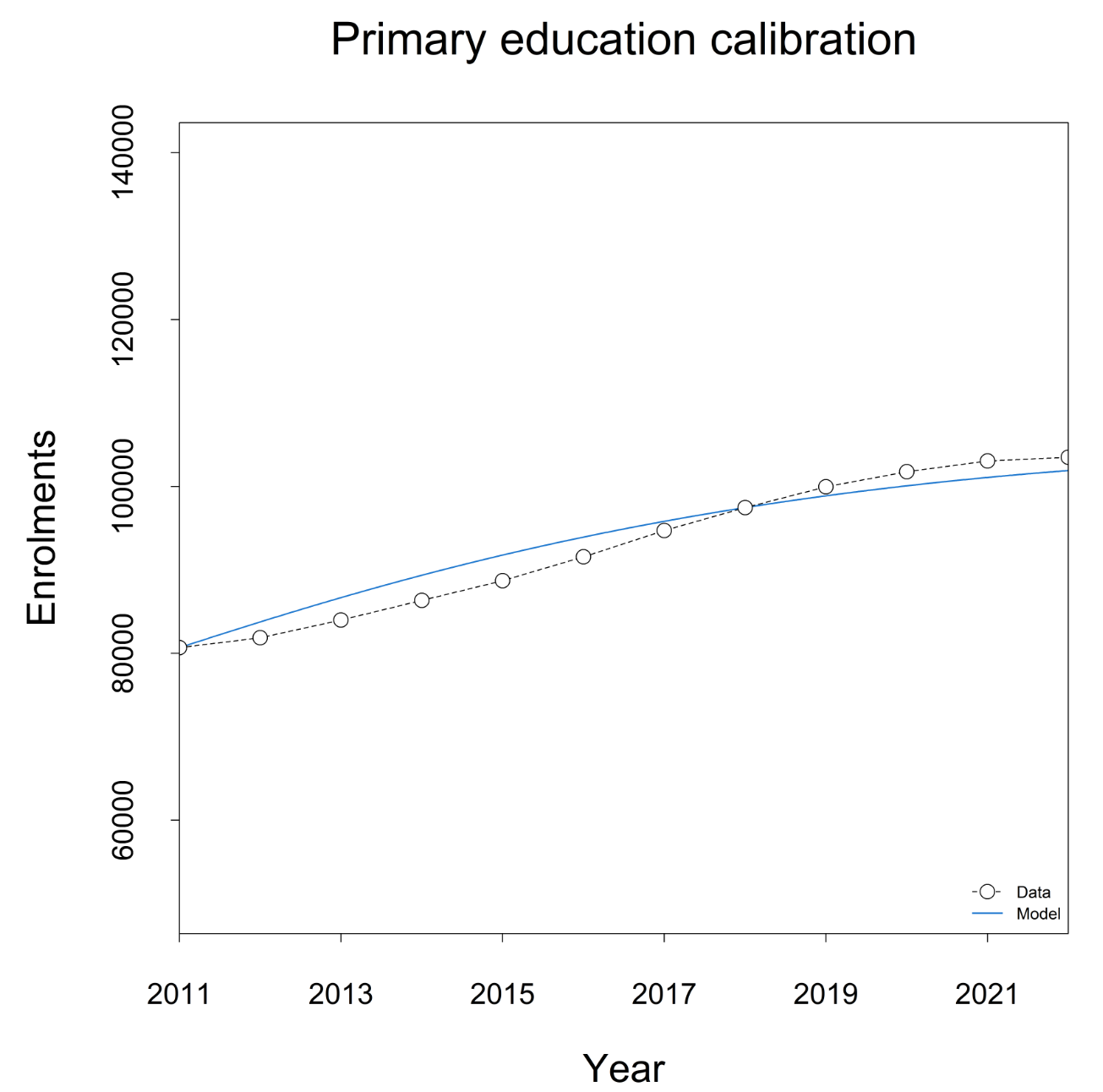
** **
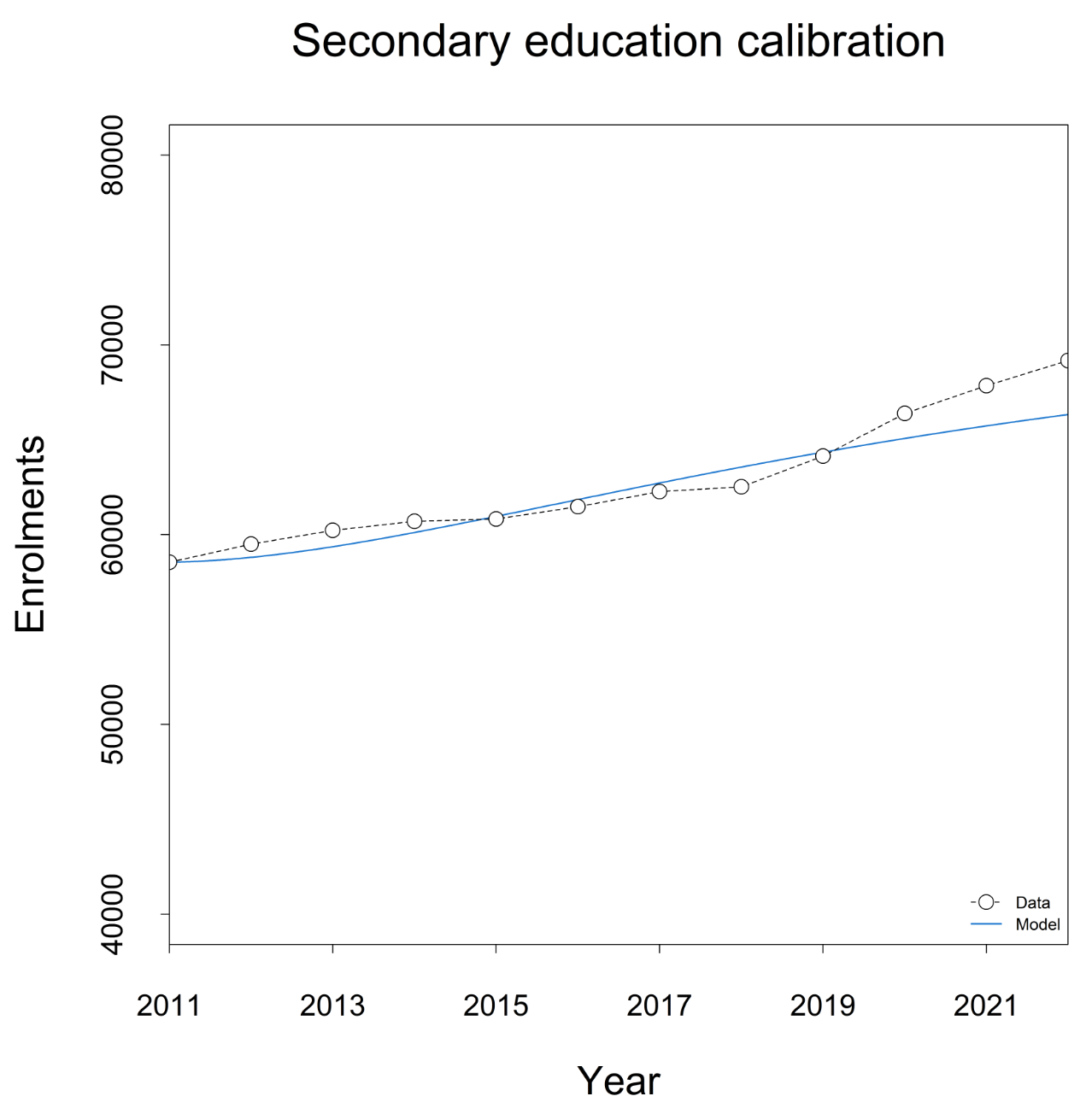
**
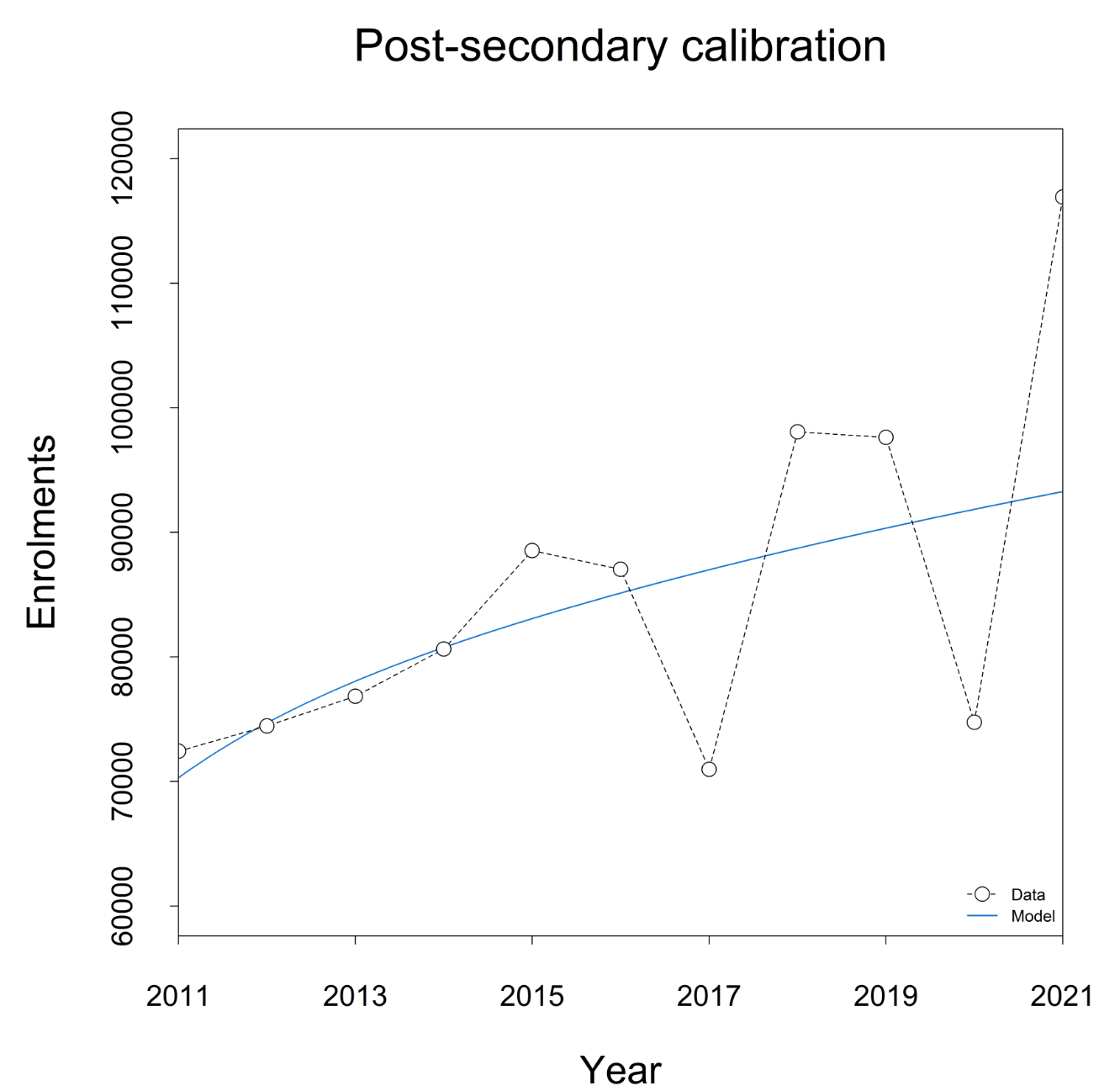


**Figure S4.** Calibration plots from the education (students) sector.

## **Education (highest level of qualification)**

The education (highest level of qualification) sector models holders of different qualifications in WSPHN. The stocks correspond to people aged 15-24 years and 25 years and older, and by their highest level of qualification. More specifically, the stocks correspond to people whose highest level of qualification is Year 12 or equivalent completion, and to people whose highest level of qualification is Certificate III or above. People not in any of these two qualification stocks correspond to people whose highest level of qualification is below Year 12 or equivalent. Each stock has a mortality outflow [15] and a net migration biflow. People who complete secondary education then flow into the “Secondary qualification only” stocks. People who then complete their first post-secondary qualification flow into the “Post-secondary qualification” stocks. People also follow the ageing chain from 15-24 years to 25 years and older. This sector is calibrated using qualifications, education and work statistics from the ABS [14].


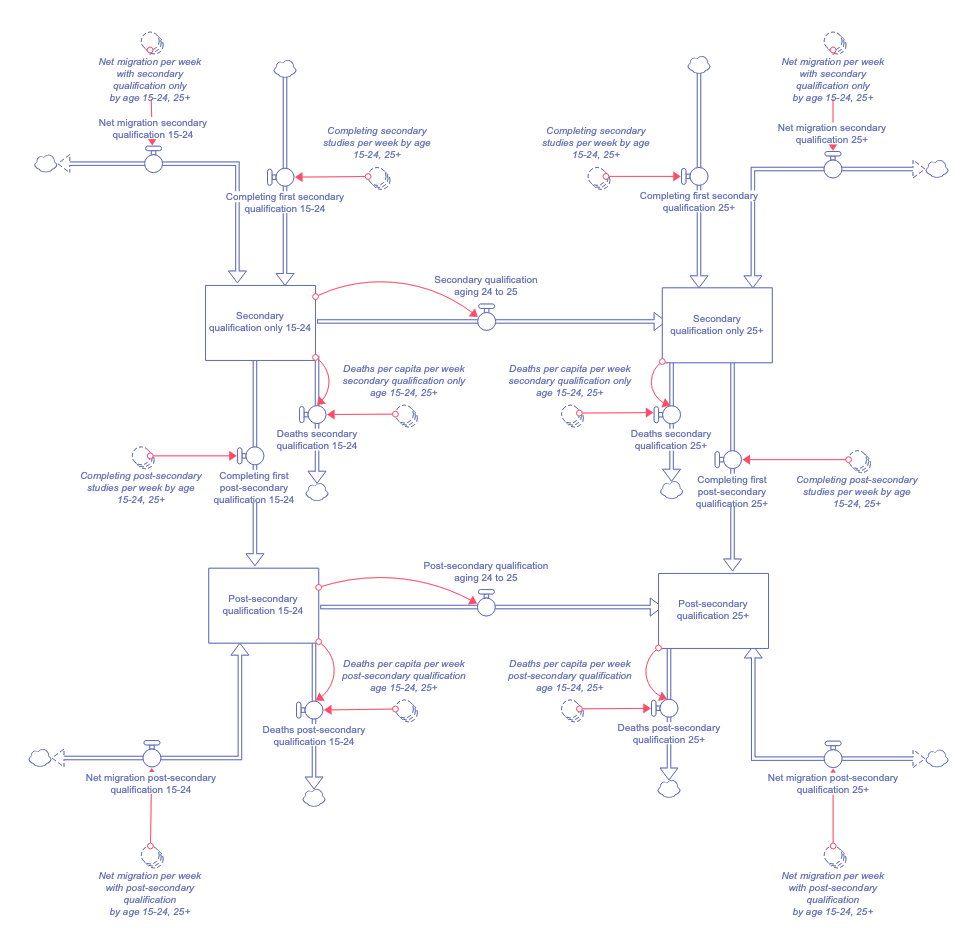


**Figure S5.** Structure of the education (highest level of qualification) sector.


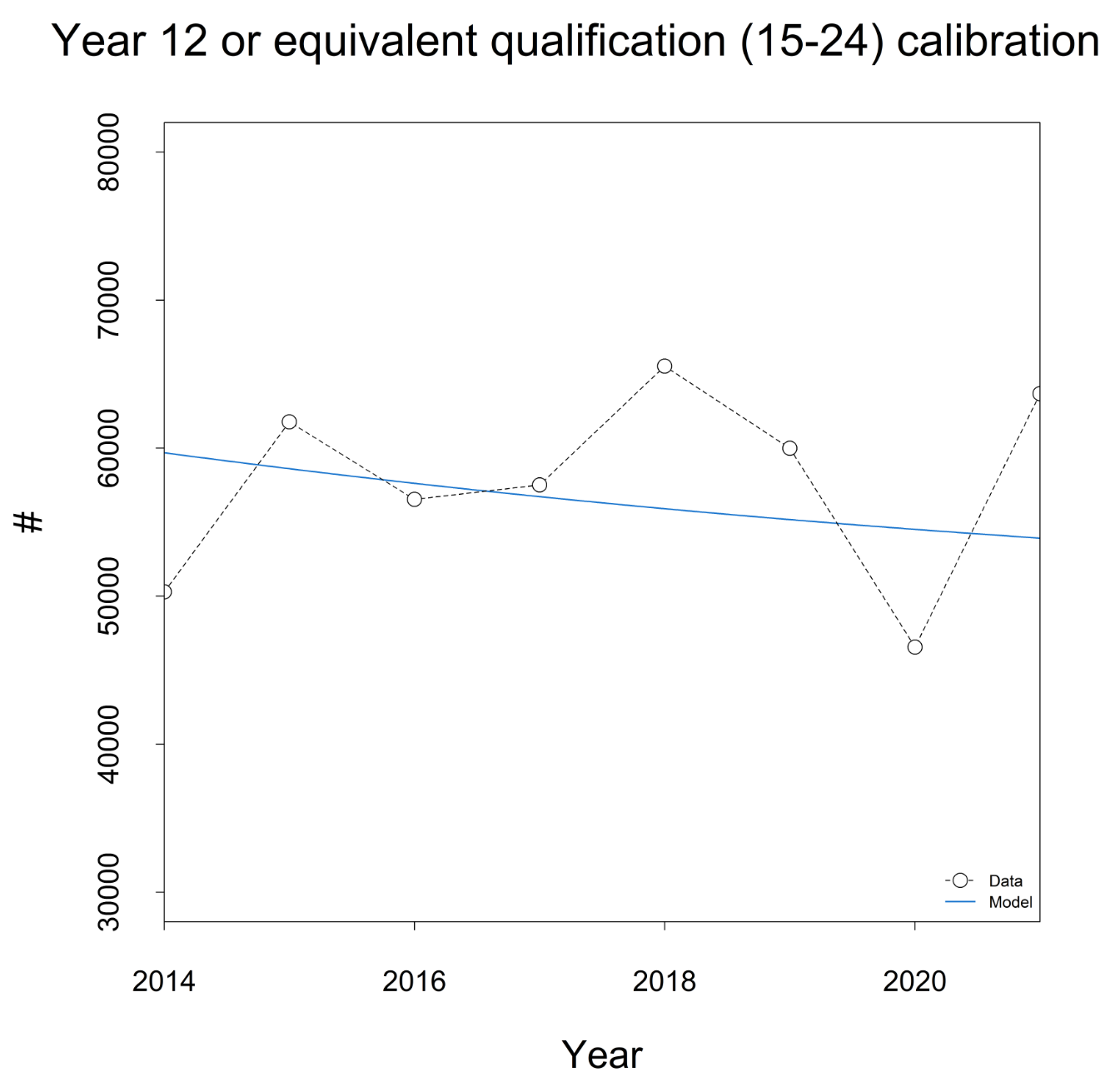

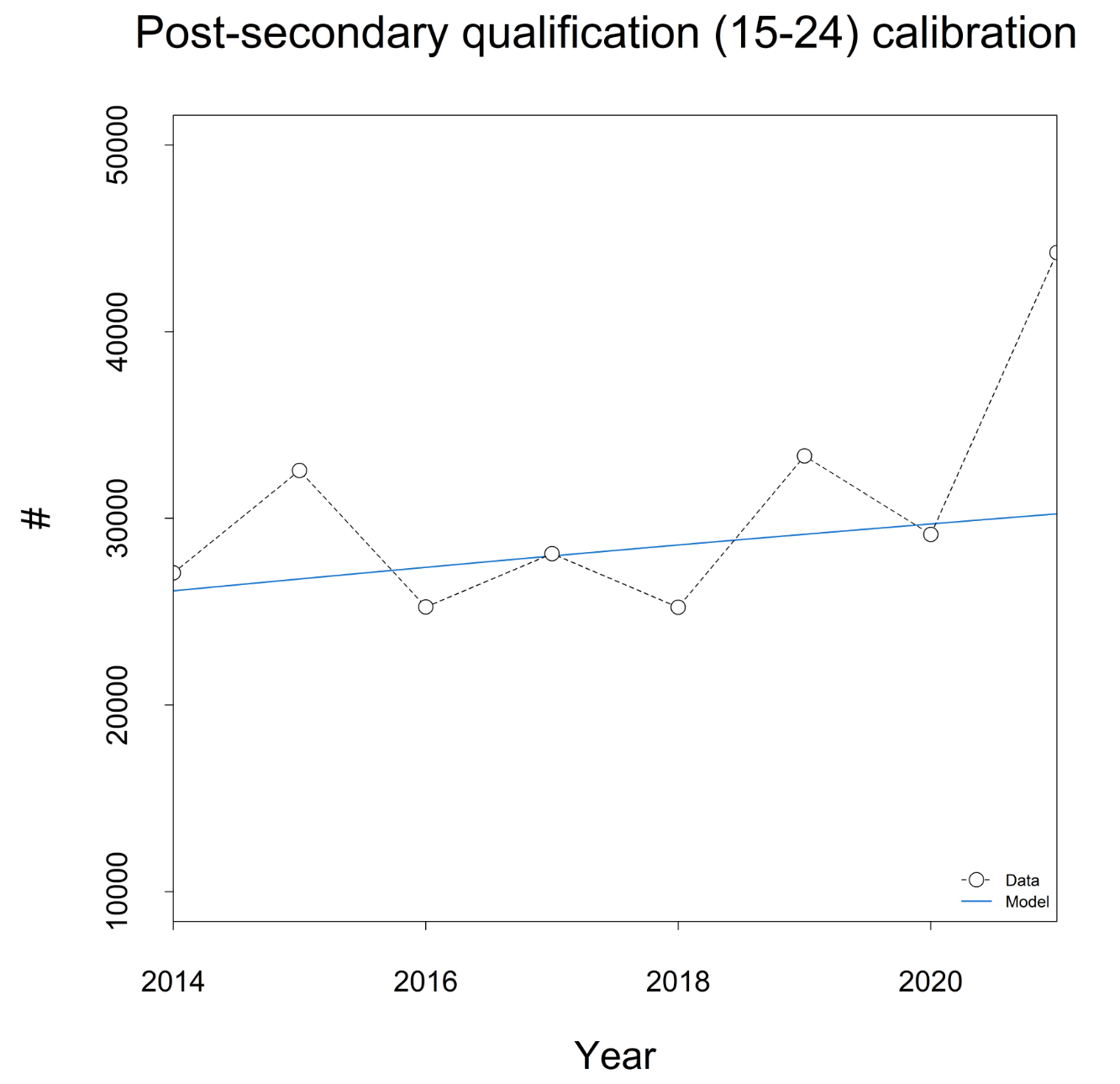


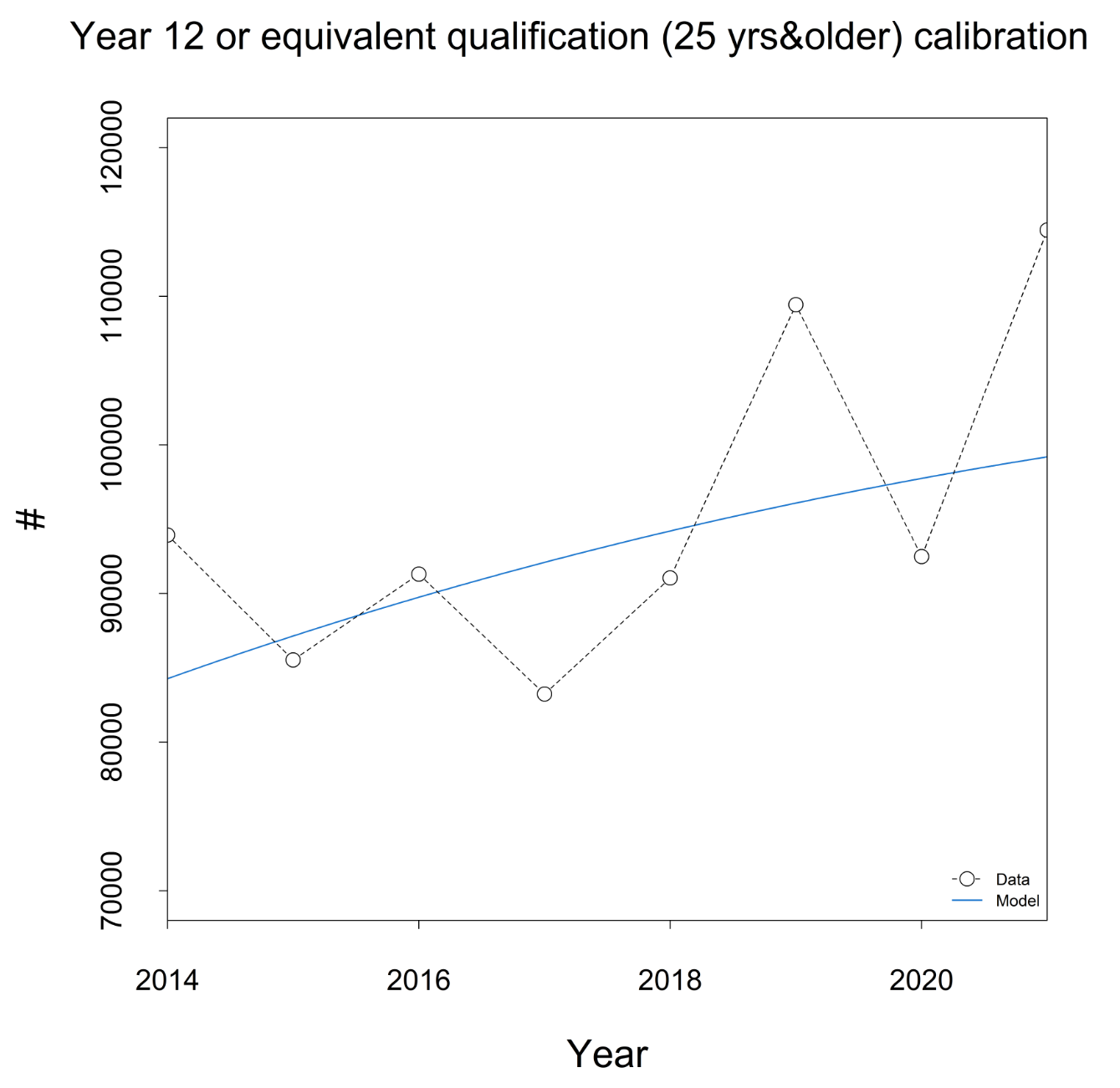

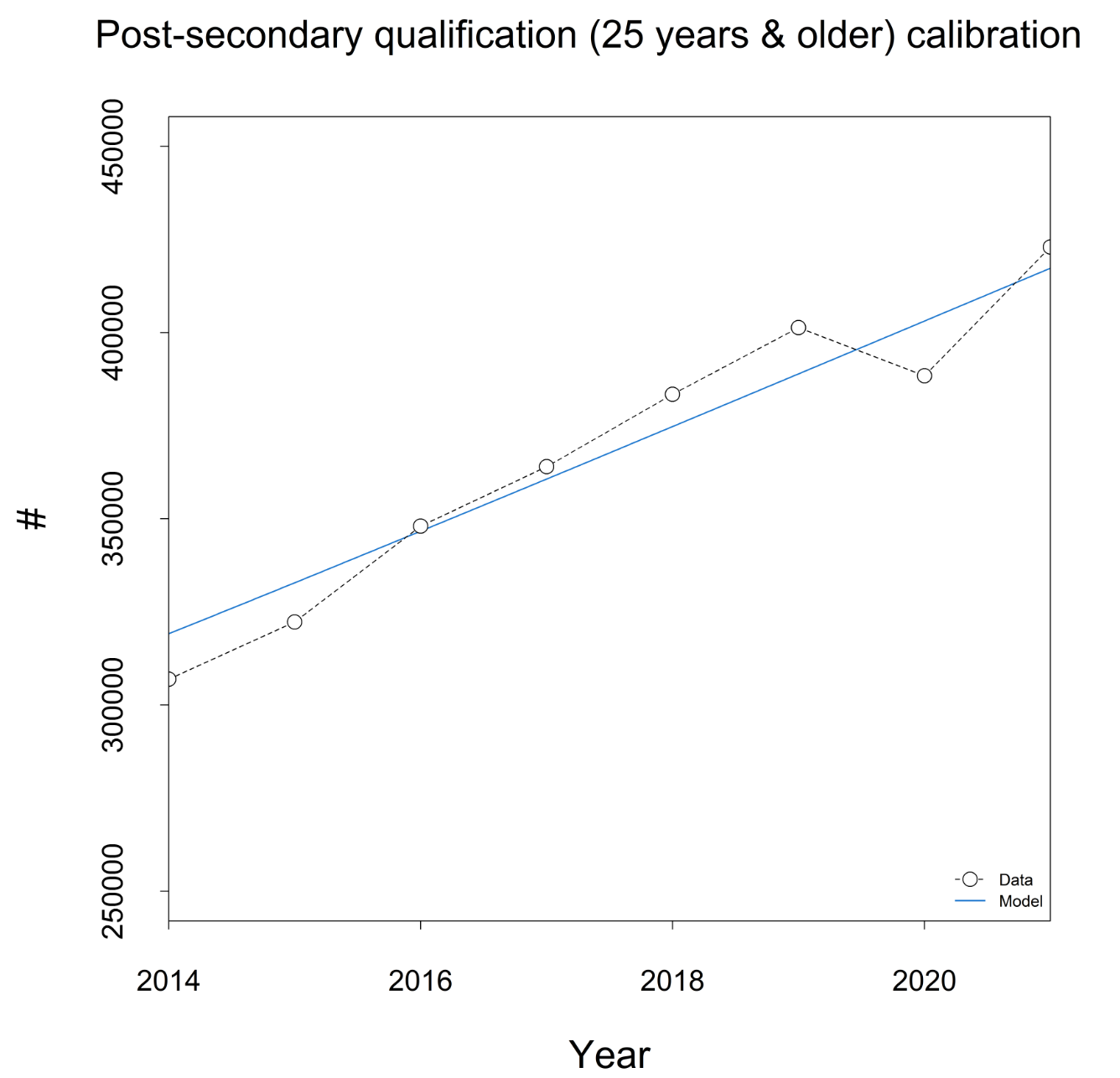


**Figure S6.** Calibration plots from the education (highest level of qualification) sector.

## **Labour force**

The labour force sector models the employment statuses and flow between these statuses of the WSPHN resident population. The stocks correspond to people aged 15-24 years and 25 years and older, and by their labour force status. More specifically, people can be either sufficiently employed, underemployed or unemployed. People who are not in either of these stocks are deemed to be not in the labour force (NILF) (e.g., retirees). Each stock has a mortality outflow [16] and a net migration biflow. People can transition between these four labour force statuses with the exception for the NILF population who must transition into unemployment prior to transitioning into employment to reflect people actively looking for work prior to being employed. The rates of transition between employed and unemployed, and between NILF and unemployed are dependent on age and levels of psychological distress / disorder [17–19] and highest levels of qualifications [14]. The rates of transition between sufficiently employed and underemployed are dependent on age and highest level of qualifications [20]. This sector is calibrated using labour force statistics from the ABS [14,21,22].

**Figure S7.** Structure of the labour force sector.


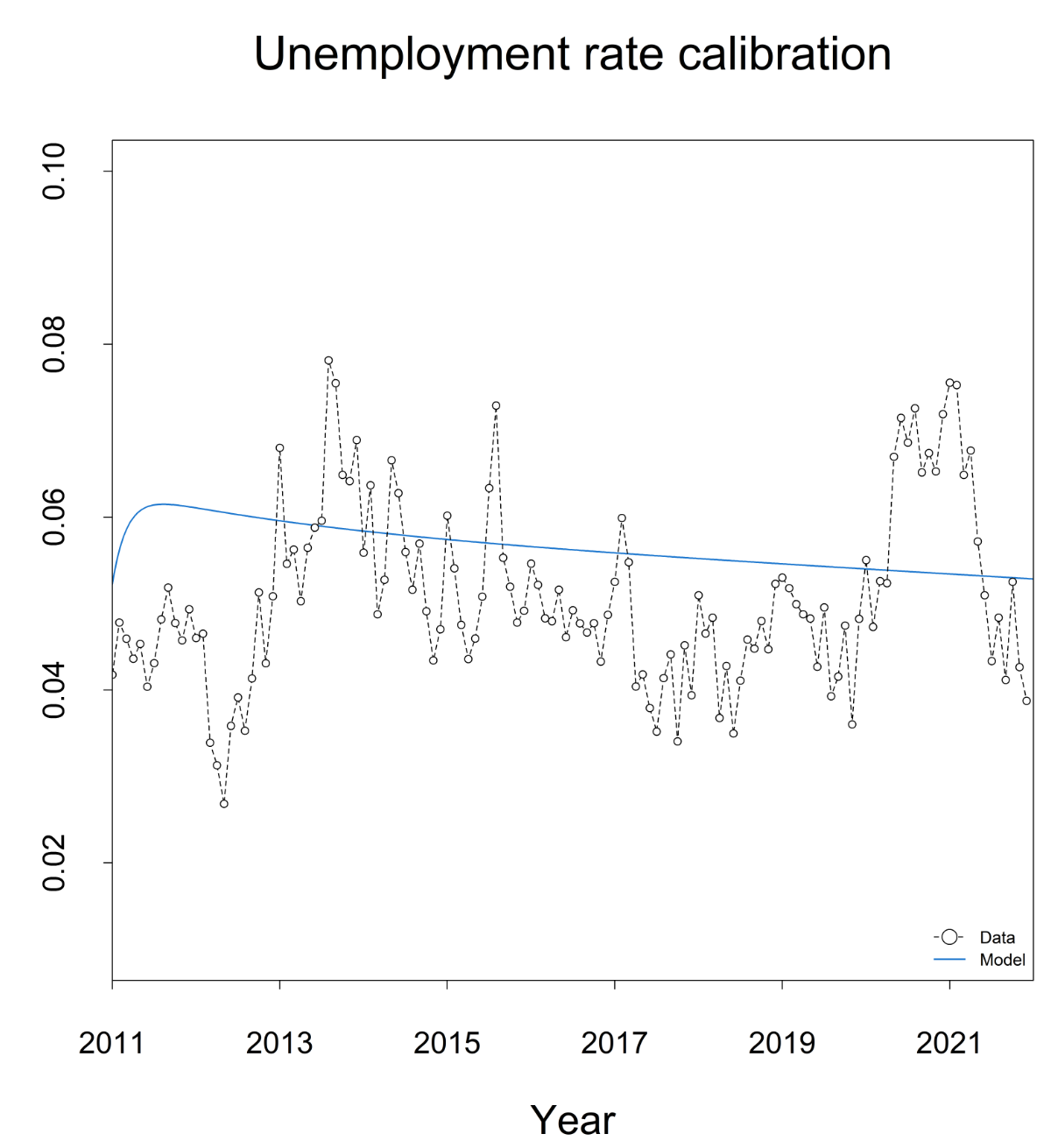

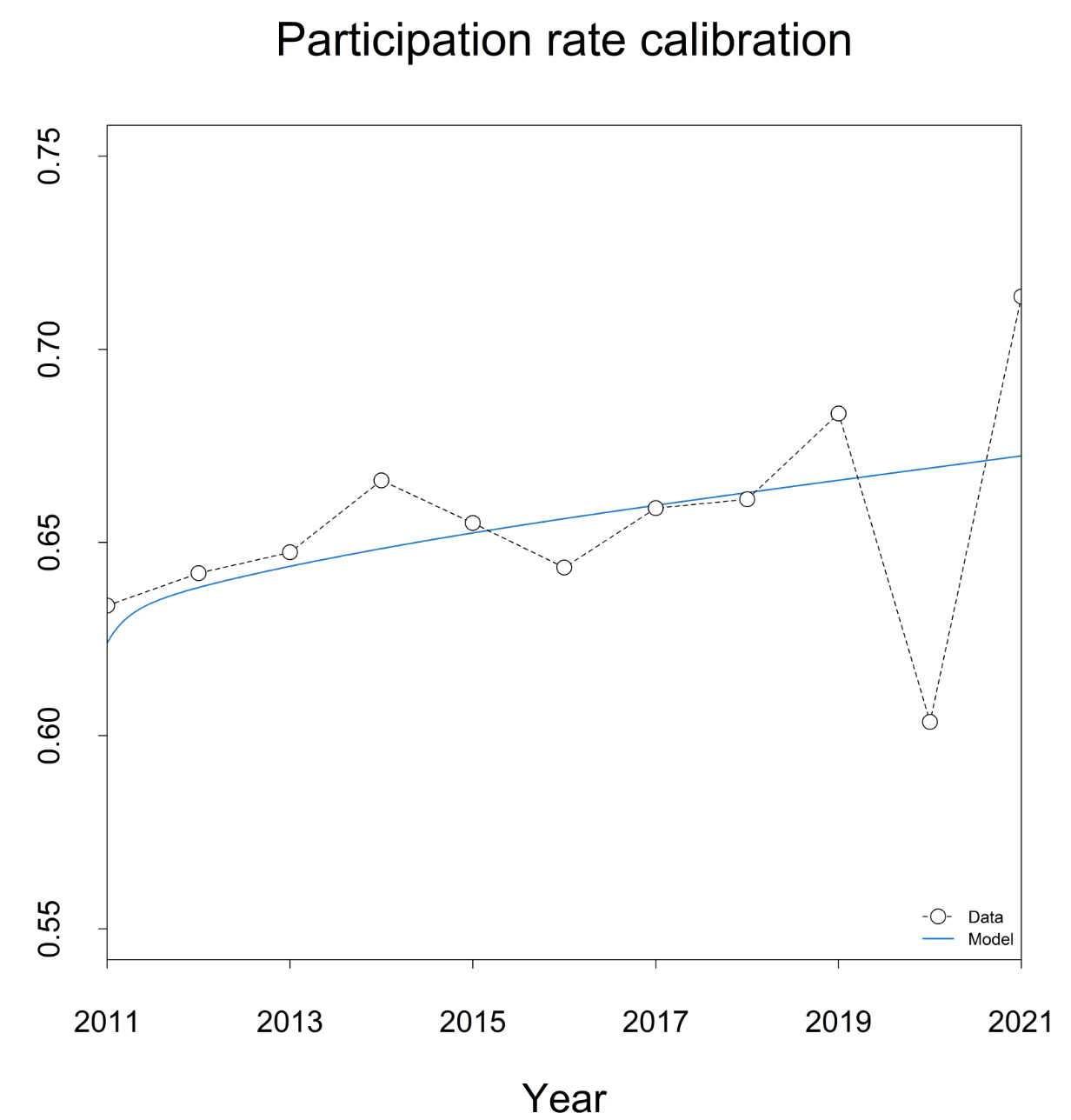

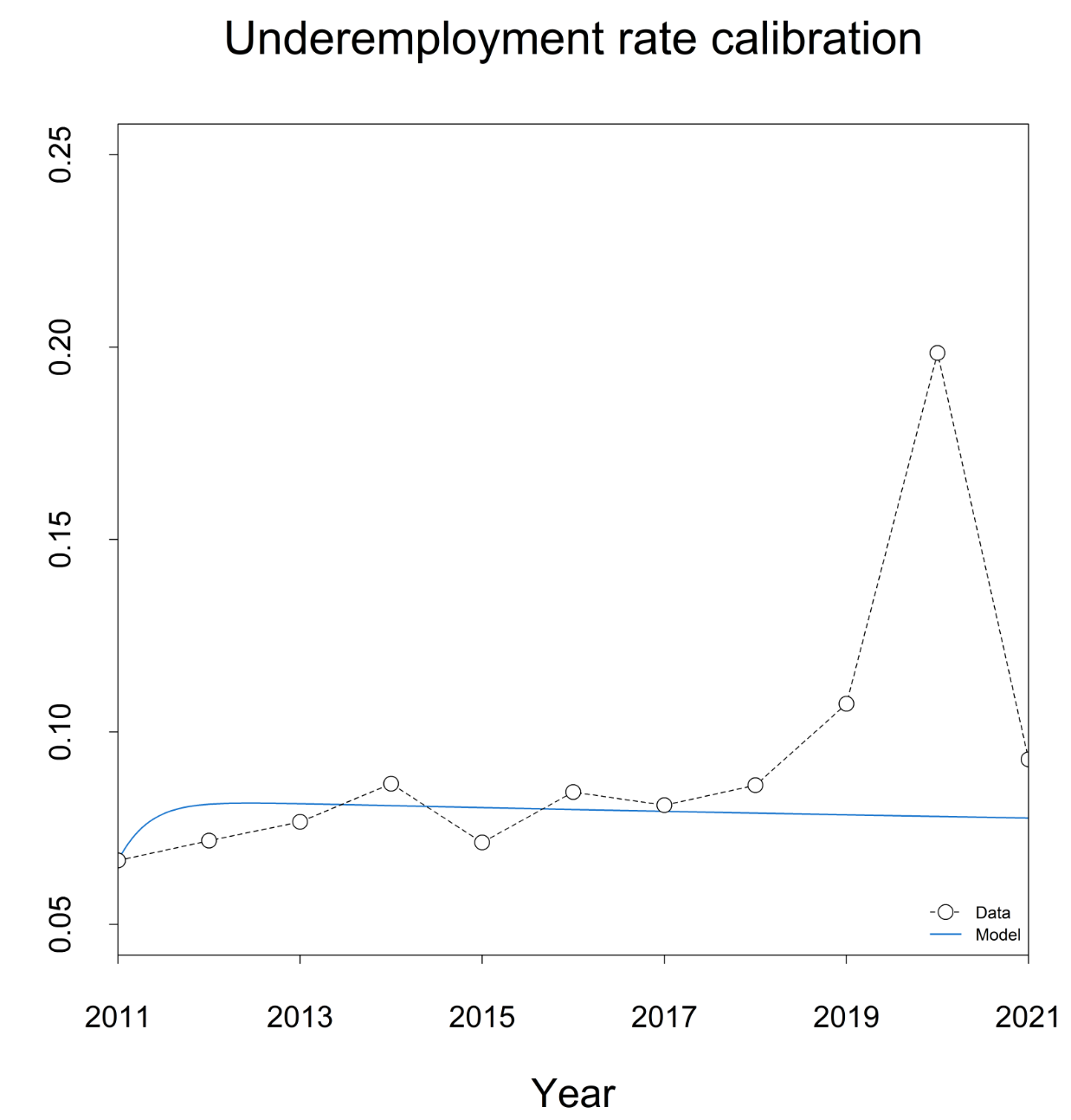


**Figure S8.** Calibration plots from the labour force sector.

## **Not in Employment, Education or Training (NEET)**

This sector models residents of WSPHN aged 15-24 years who are not in education, employment or training (NEET). This sector uses model outputs from the labour force and education (students) sectors to calculate the numbers of young people who are NILF and not currently studying. This sector is calibrated using education and work statistics from the ABS [14].


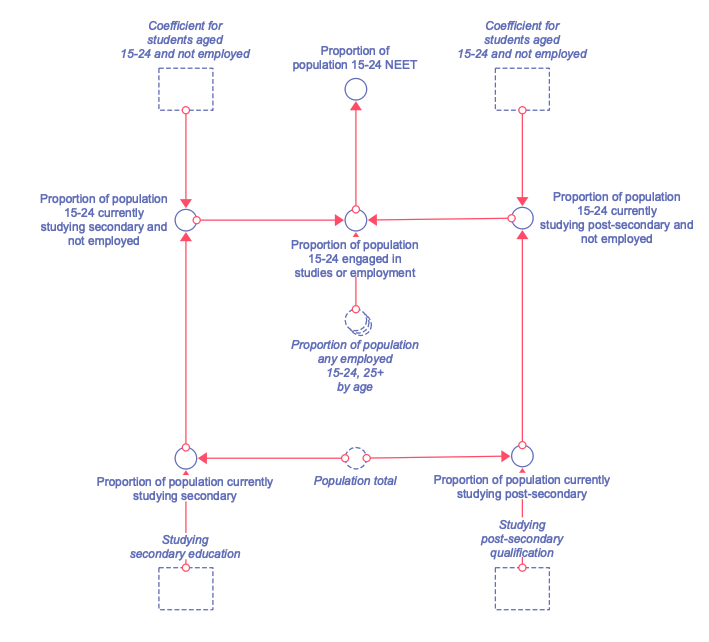


**Figure S9.** Structure of the NEET sector.

**
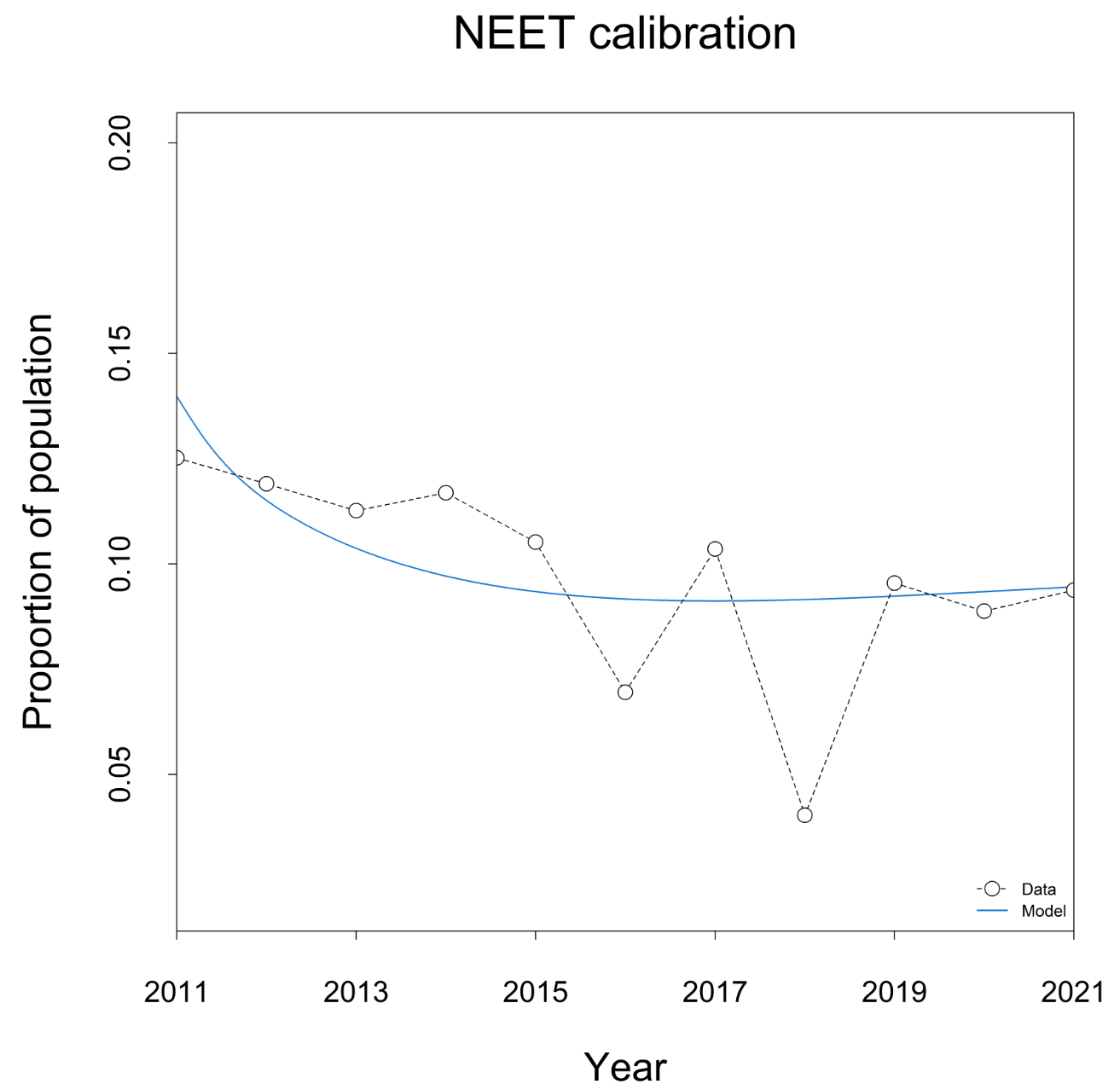
**

**Figure S10.** Calibration plot from the NEET sector.

## **Psychological distress / disorder**

This sector models the prevalence of psychological distress and 12-month psychological disorder in people aged 12-14 years, 15-17 years, 18-24 years, and 25 years and older in WSPHN. These stocks model the population with low psychological distress according to the Kessler Psychological Distress Scale (K10) [23], the population with moderate to very high psychological distress who do not meet the criteria for a 12-month psychological disorder, and the population with moderate to very high psychological distress who meet the criteria for a 12-month psychological disorder. Transition rates between these three levels of psychological distress / disorder are dependent on age, rates of homelessness [24], unemployment [25], underemployment [26,27], substance misuse [25,28], engagement and disengagement with the mental health services system, the levels of social cohesion, rate of family and domestic violence [29,30], rate of involvement in a serious legal proceeding [31] and the rate of childhood exposure to difficulty [32]. Each stock has a mortality outflow [33] and a net migration biflow, and the population ages following an ageing chain across each level of psychological distress / disorder. This sector is calibrated using psychological distress prevalence data as measured by the K10 from the NSW Centre for Epidemiology and Evinence [17,18], the ABS’ National Health Survey [34], and the Young Minds Matter Survey [35]. The prevalence estimates for 12-month psychological disorder were modelled from data from the ABS’ National Study of Mental Health and Wellbeing [25]. Calibration for 12-14-year-olds and 15-17-year-olds used data and modelled estimates inferred using the the Young Minds Matter Survey [35], the NSW Centre for Epidemiology and Evinence [17,18], the ABS’ National Health Survey [34].

**Figure S11.** Structure of the psychological distress / disorder sector.

**
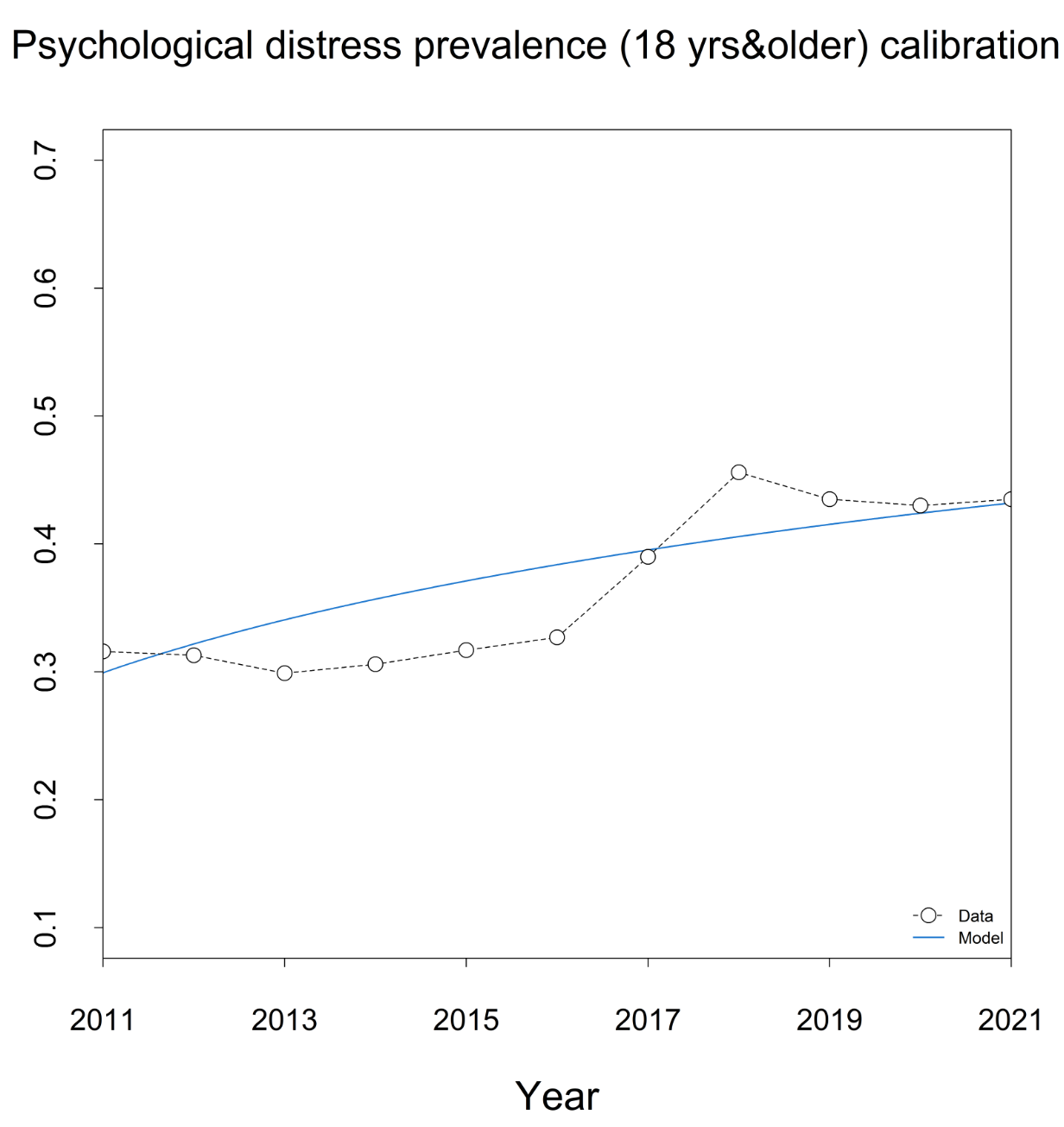
**

**
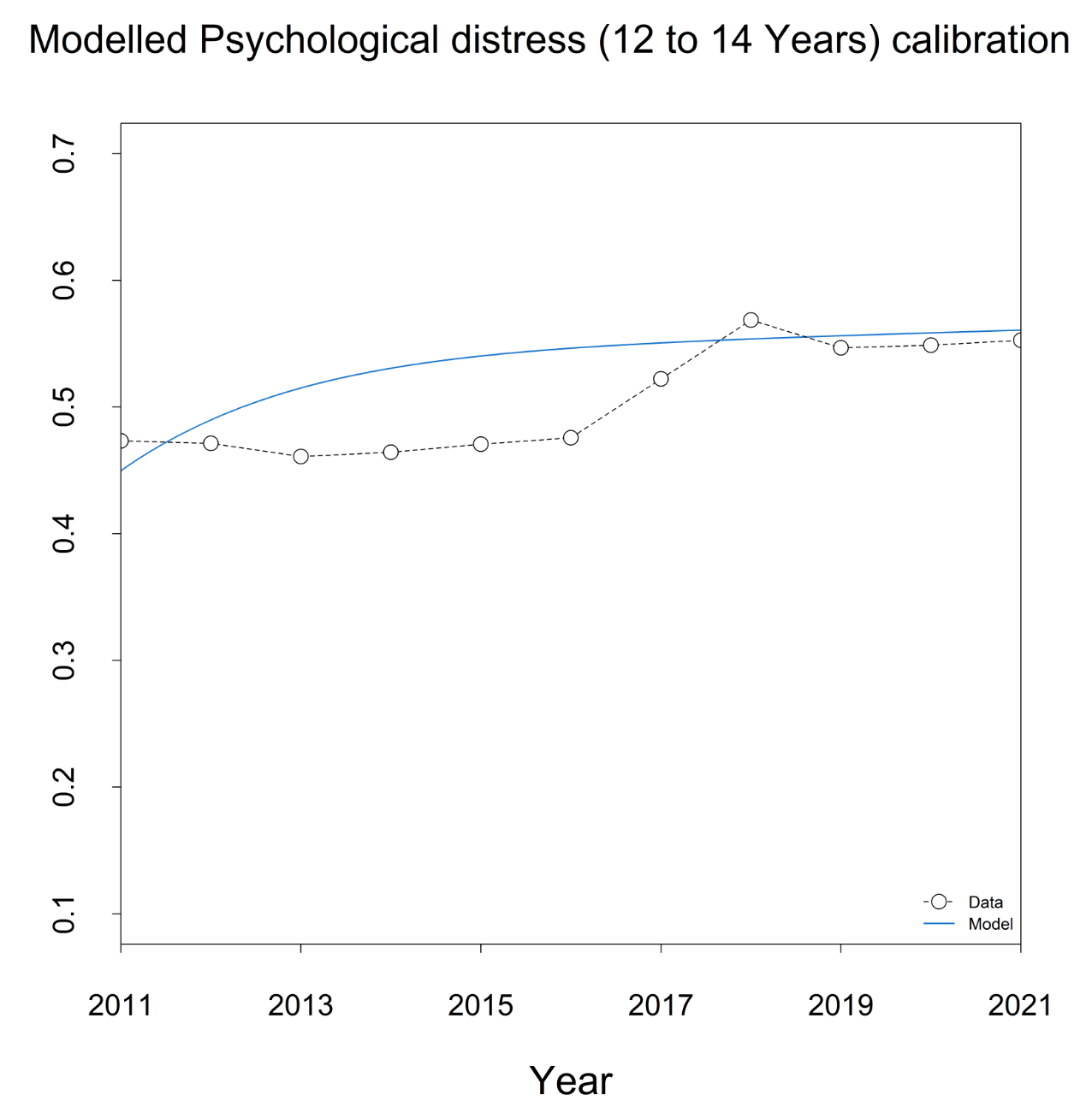
**
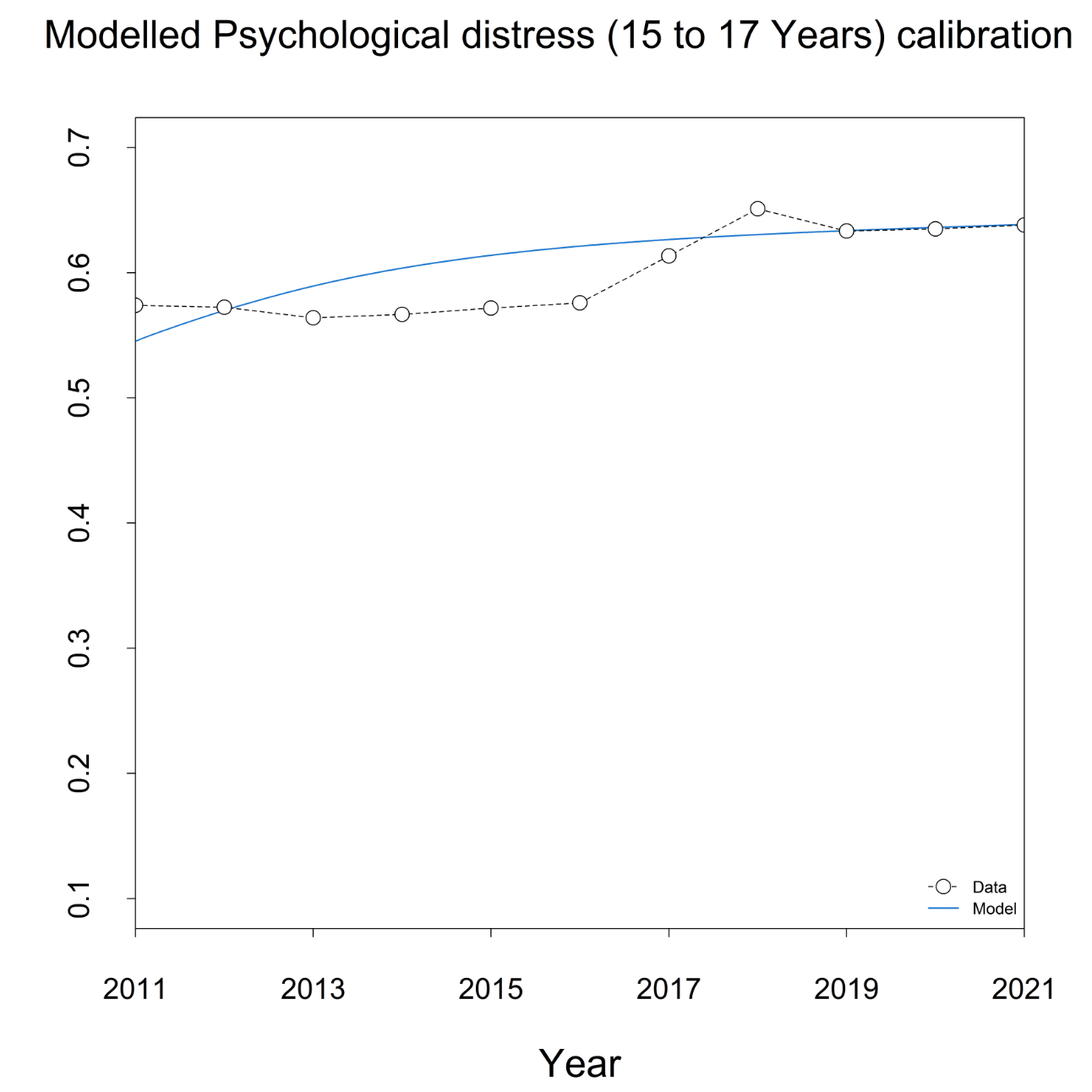

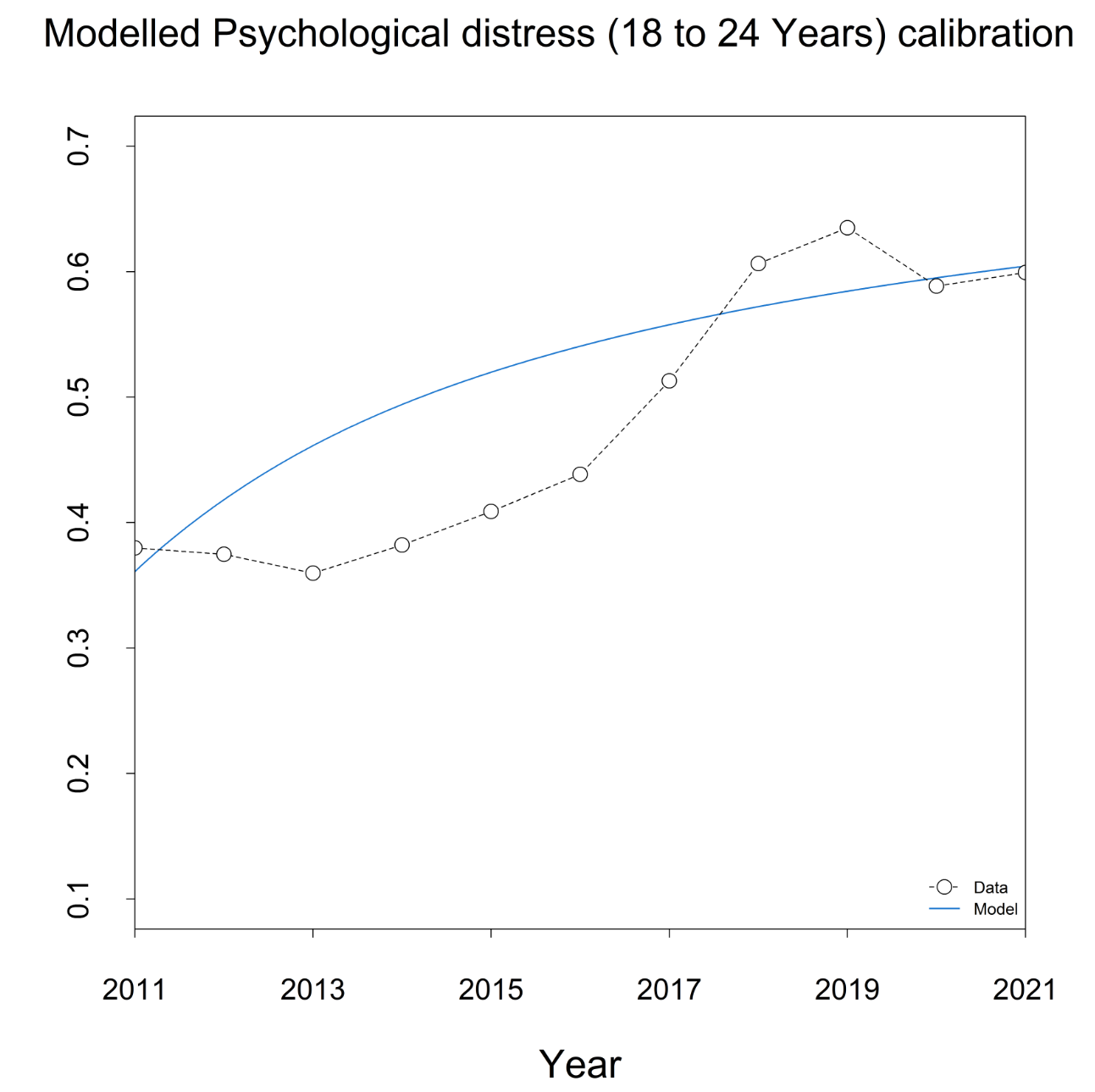

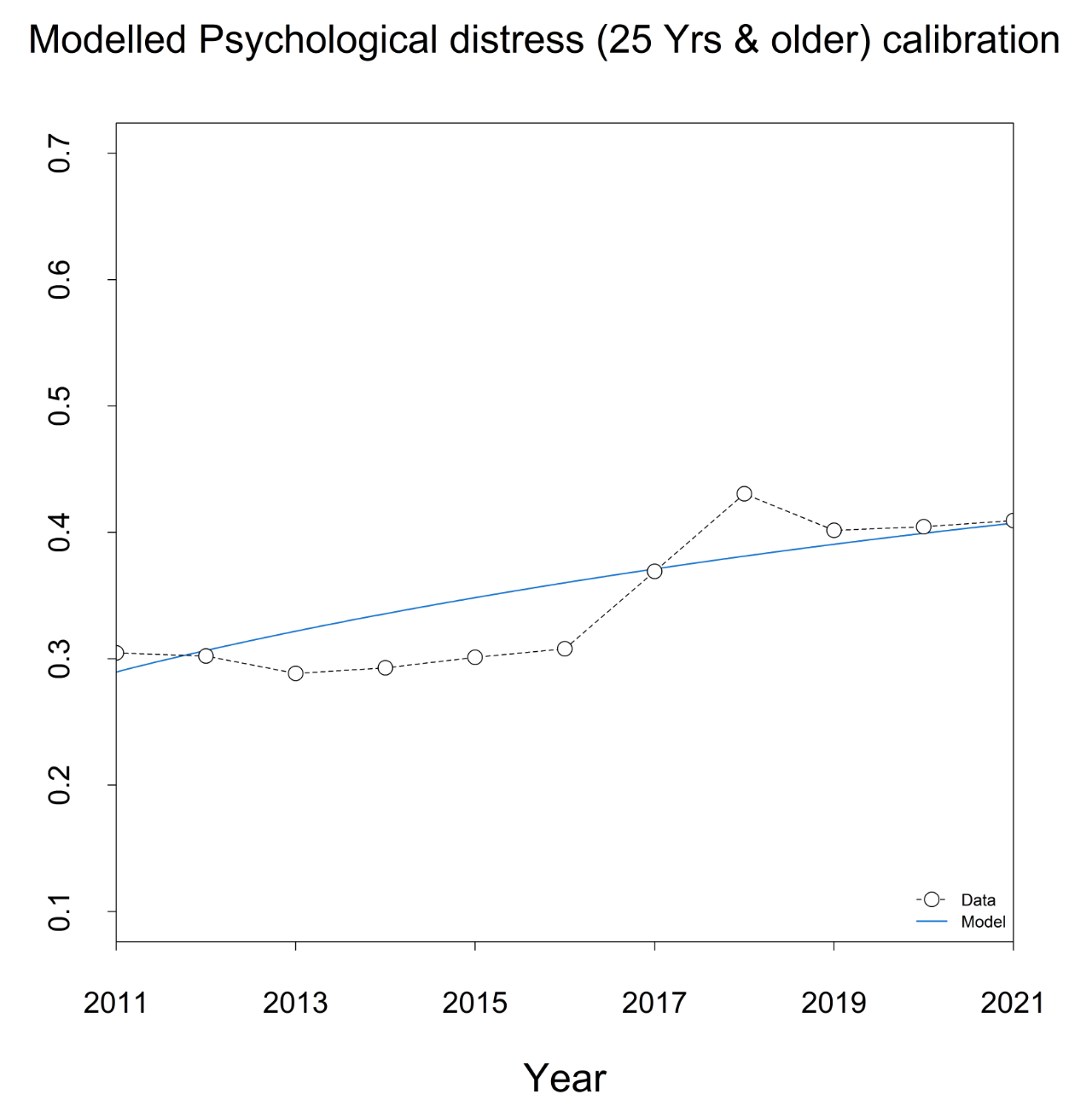


**Figure S12.** Calibration plots from the psychological distress / disorder sector.

## **Strengths and Difficulties**

This sector models the prevalence of adverse early life exposures as indicated by scores on the Strengths and Difficulties Questionnaire (SDQ) for children aged 0-4 years and 5-11 years. These stocks model the population with “Close to average”, “Slightly raised” and “High” SDQ scores. Children are assumed to be born with close to average levels of SDQ and hence flow into the “Close to average SDQ” stock for 0-4-year-olds. People can flow between close to average and slightly raised levels of SDQ, and between slightly raised to high levels of SDQ with rates dependent on age and rates of engagement and disengagement with the mental health services systems and the levels of social cohesion. Each stock has a mortality outflow and a net migration biflow, and the population ages following an ageing chain across each level of SDQ stocks. This sector is calibrated using SDQ data from the Longitudinal Study of Australian Children [36]. Calibration for 0-4-year-olds used modelled estimates inferred using data for 5-11-year-olds. Please note that, as part of the user agreement between the authors and the Longitudinal Study of Australian Children, SDQ data at the PHN level of geographic granularity cannot be shown. As such, calibration plots for the Strengths and Difficulties sector will not be shown here.


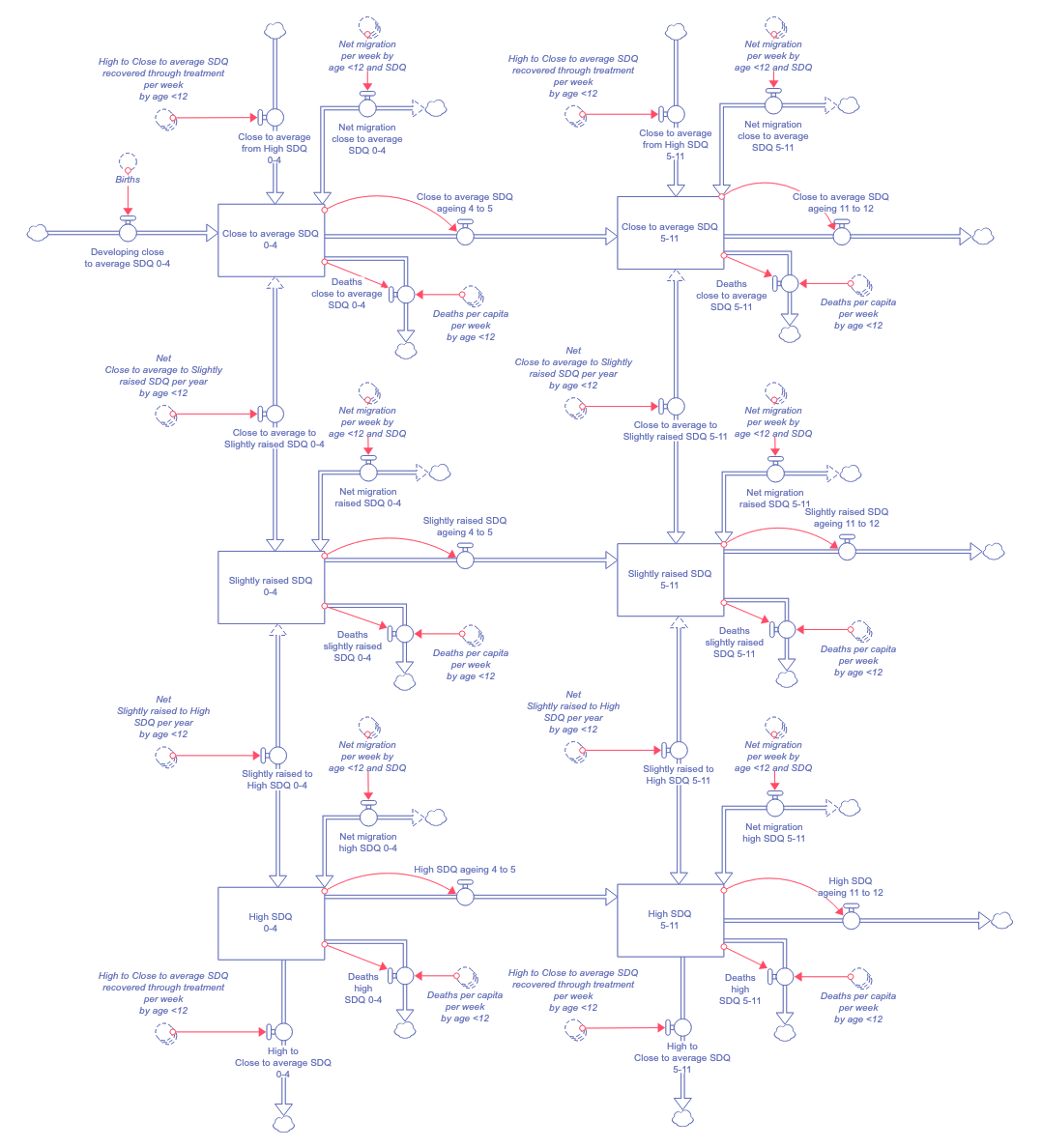


**Figure S13.** Structure of the Strengths and Difficulties sector.

## **Homelessness**

This sector consists of six stocks modelling people experiencing homelessness aged 0-4 years, 5-11 years, 12-14 years, 15-17 years, 18-24 years, and 25 years and older. Each stock has a mortality outflow [37] and a net migration biflow. People aged 15 and older enter homelessness at rates which are dependent on age, levels of psychological distress / disorder, unemployment rates, and substance misuse rates [38]. For people under 15 years of age, rates of entering homelessness are dependent on age. People exit homelessness at rates dependent on the mean duration of homelessness [39]. People age into older stocks following the ageing chain.

This sector was calibrated using homelessness statistics from the ABS [24].


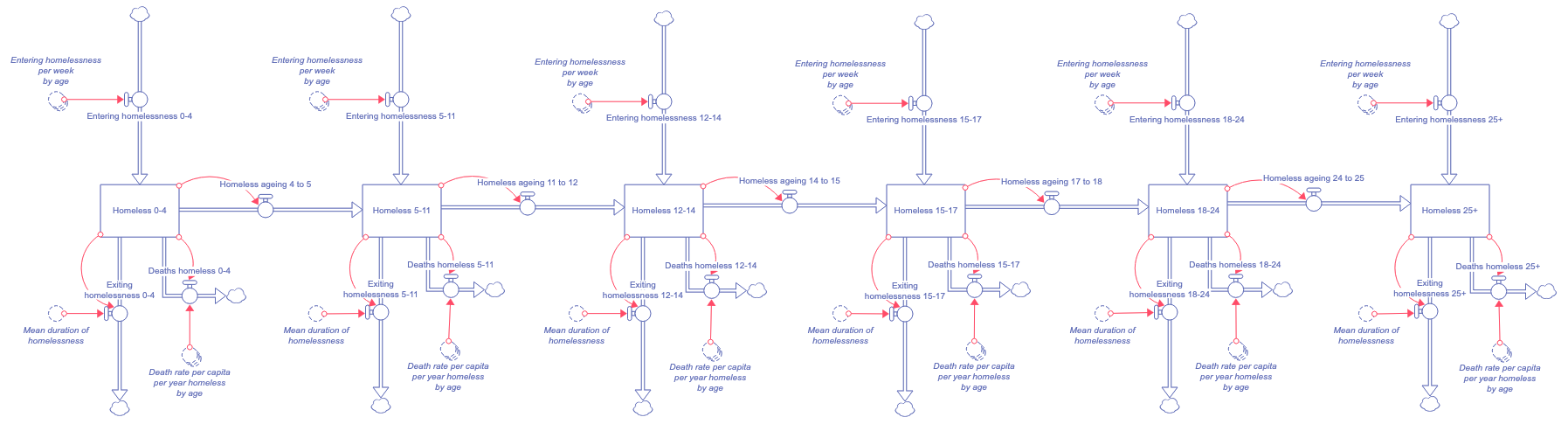


**Figure S14.** Structure of the homelessness sector.


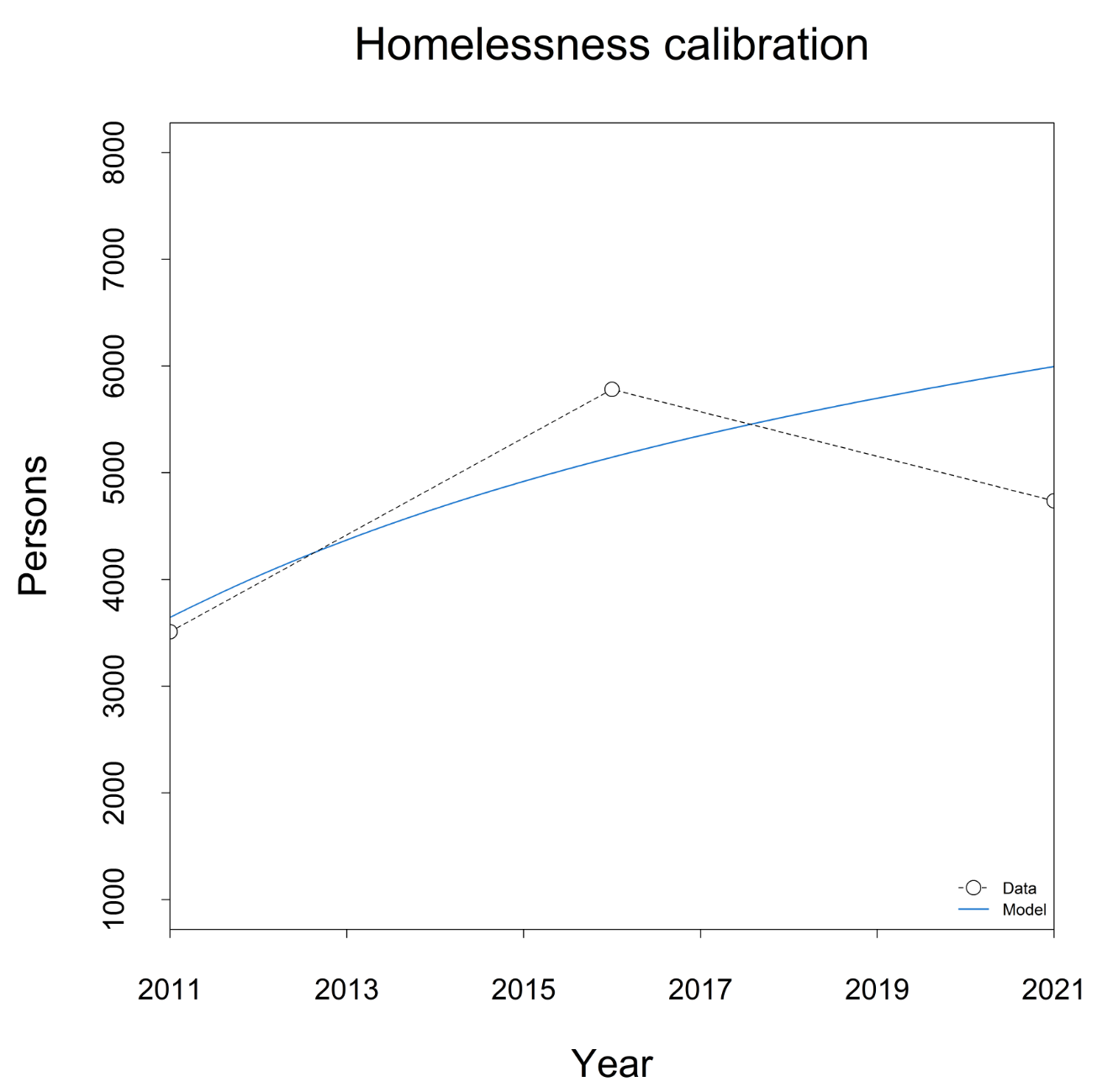


**Figure S15.** Calibration plot from the homelessness sector.

## **Family & domestic violence (FDV)**

This sector models the rate of family and domestic violence for people aged 0-4 years, 5-11 years, 12-14 years, 15-17 years, 18-24 years, and 25 years and older. It considers the impact of secondary school completion and unemployment rates [40,41] on FDV for different age groups. This sector was calibrated using the data for victims of family and domestic violence related offences from the ABS [42].


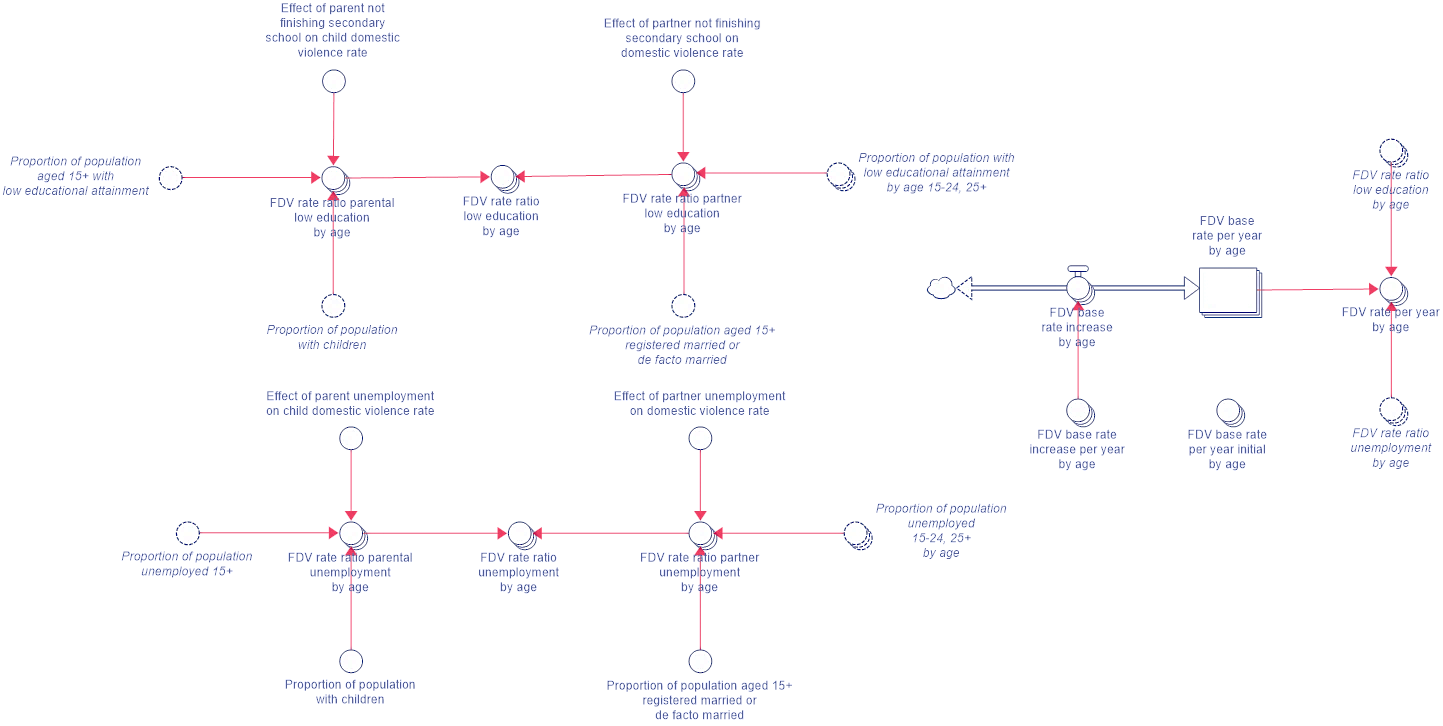


**Figure S16.** Structure of the FDV sector.


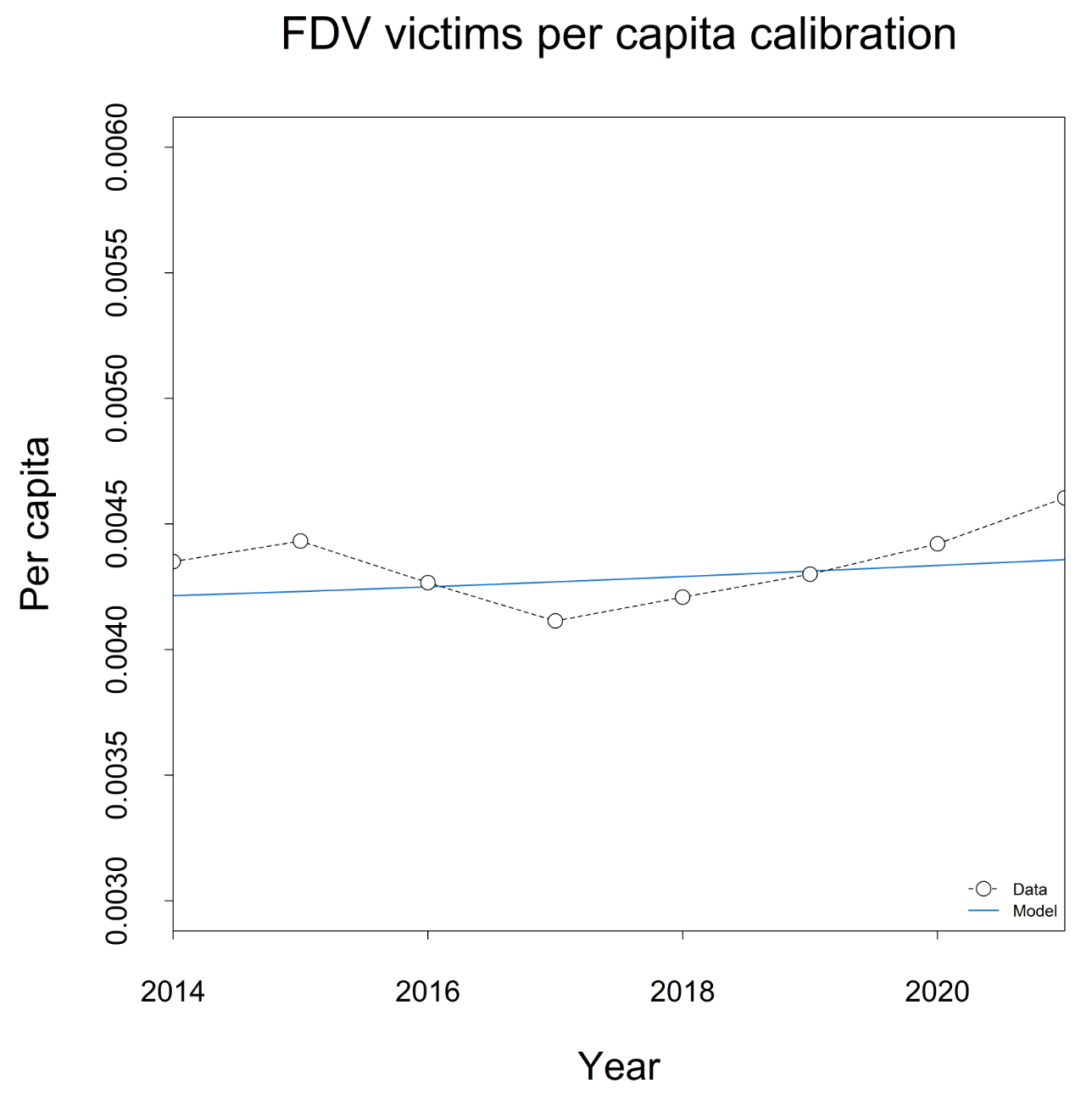


**Figure S17.** Calibration plot from the FDV sector.

## **Youth justice**

This sector models the number of offenders aged 12-14 years, 15-17 years, 18-24 years, and 25 years and older in the WSPHN catchment area. It considers the impact of proximate crime risk factors (i.e., mental health disorder [43–45] and Substance misuse [46]) and distant crime risk factors (i.e., psychological distress [44,47], homelessness [48], family and domestic violence [49,50], and NEET [51]). It models the flow of offenders into different pathways in the justice system (i.e., non-court action (warning or caution), youth justice conference, or court) calibrated based on the BOSCAR statistics and ABS data [42,52–54].

Accordingly, being involved in a serious legal proceeding for the first time is considered as a risk factor for distress onset in the psychological distress sector.^31^

**Figure S18.** Structure of the youth justice sector.


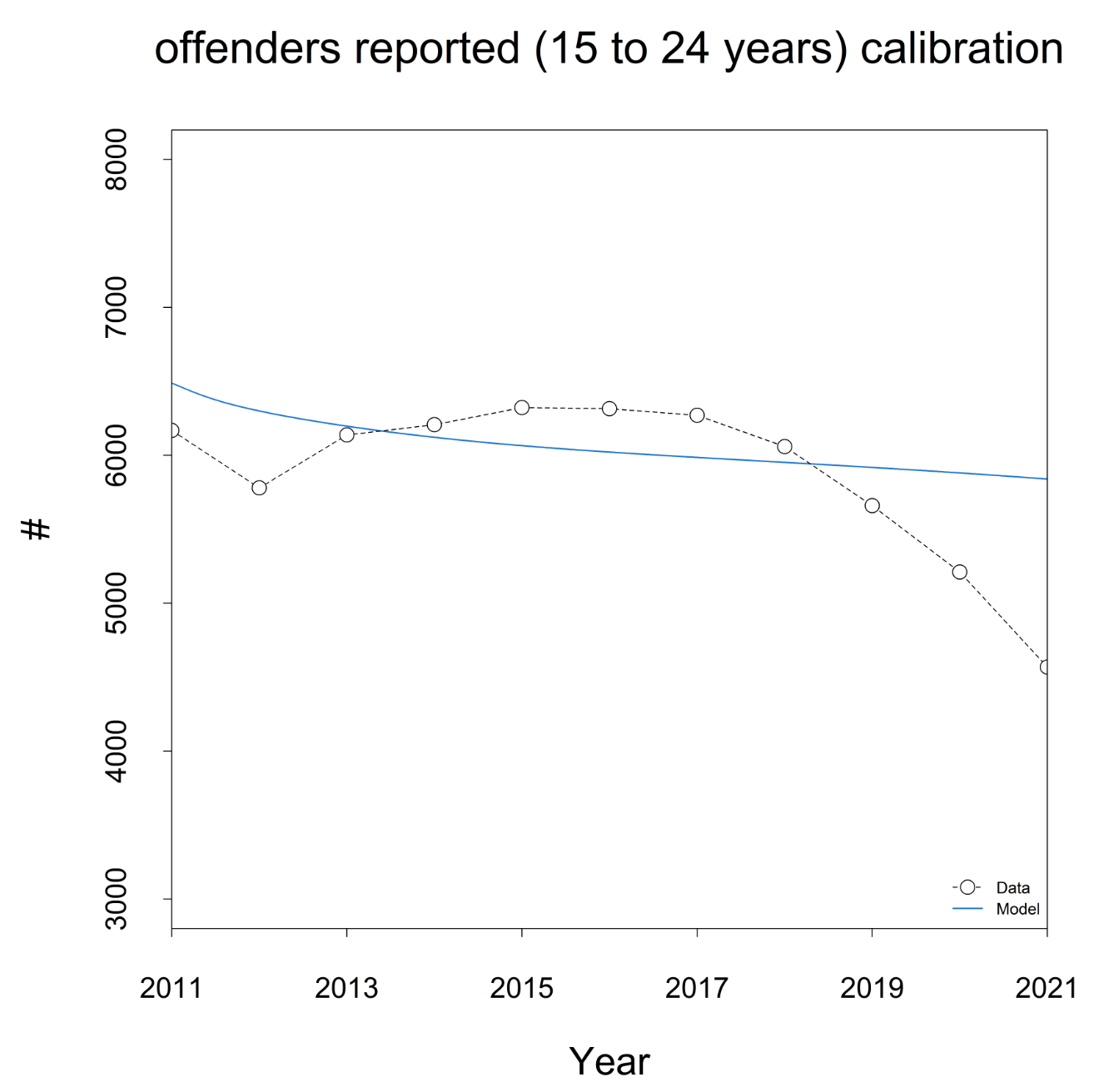


**Figure S19.** Calibration plot from the youth justice sector.

## **Substance misuse (substance misuse disorder)**

This sector models the prevalence of substance misuse in the WSPHN resident population. The stocks correspond to people aged 15-24 years and 25 years and older who meet the criteria for 12-month substance use disorder. Each stock has a mortality outflow [55] and a net migration biflow, and the population ages following an ageing chain. Each stock has a disorder onset and recovery biflow which reflect the rates of onset of and the non-treatment based recovery from substance misuse disorder. The onset rates are dependent on age, prevalence of psychological distress / disorder [56], homelessness [57] and NEET [25,58]. Each stock also has a recovery through treatment outflow representing people who recover from substance misuse disorder through treatment with services. This sector is calibrated using national 12-month substance use disorder data from the ABS’ National Study of Mental Health and Wellbeing [25].


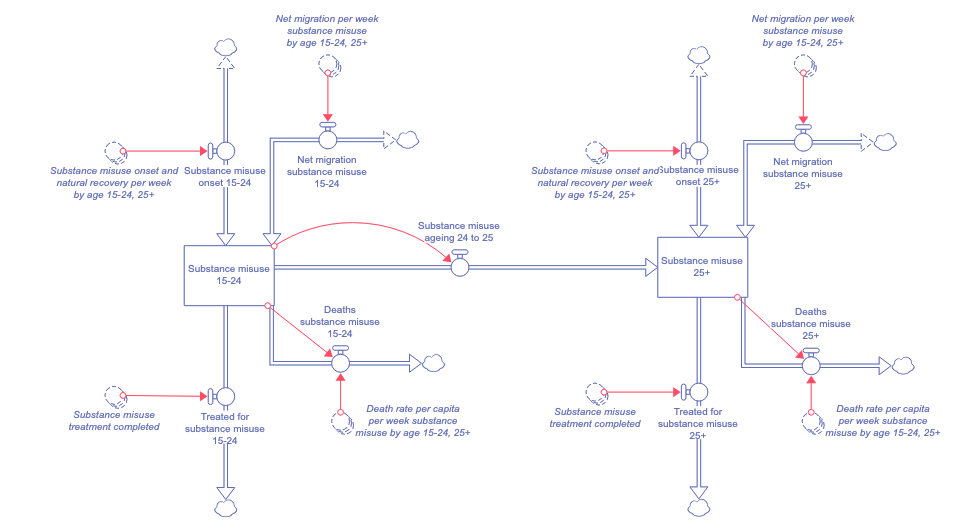


**Figure S20.** Structure of the substance misuse (substance misuse disorder) sector.


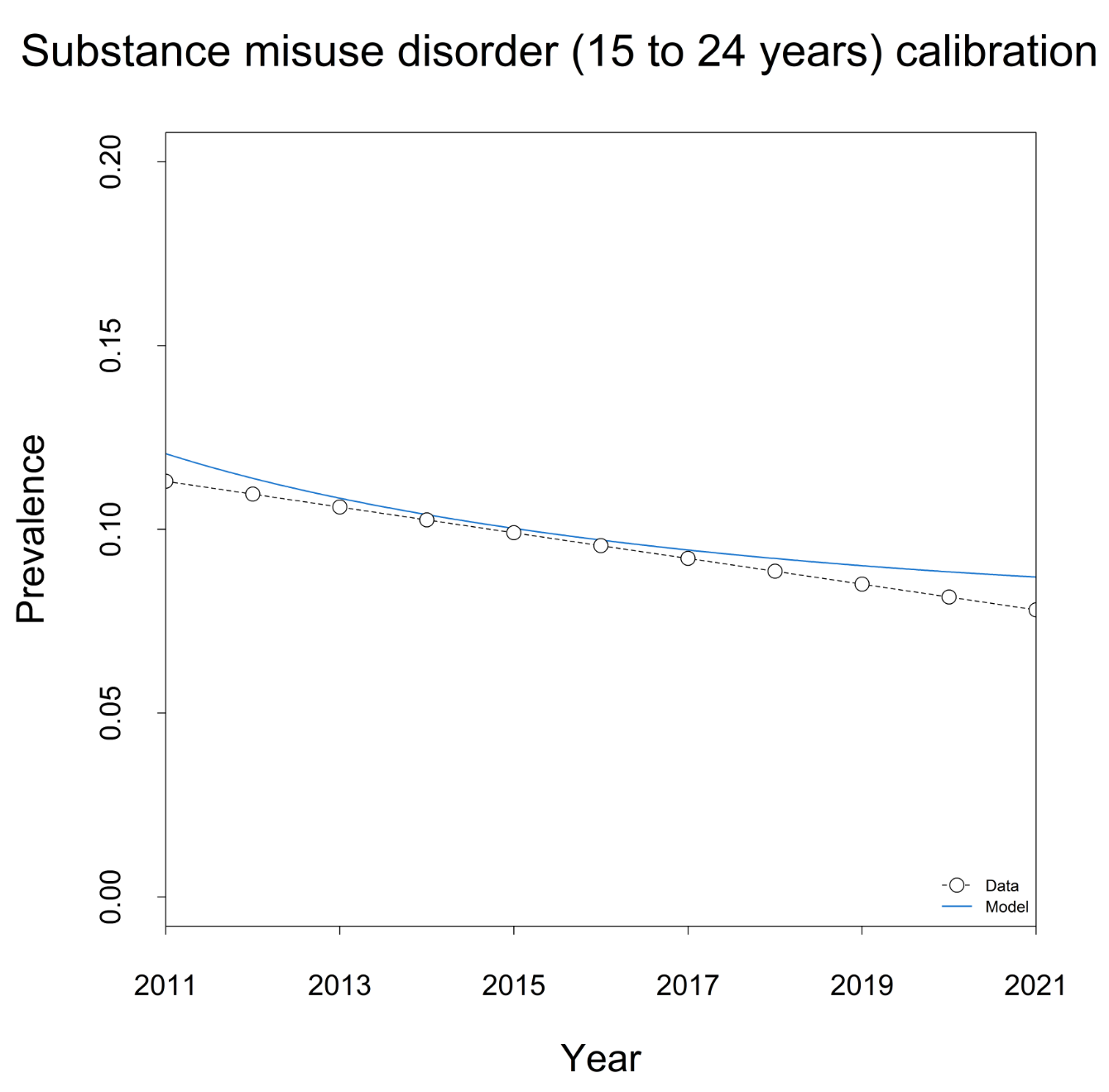

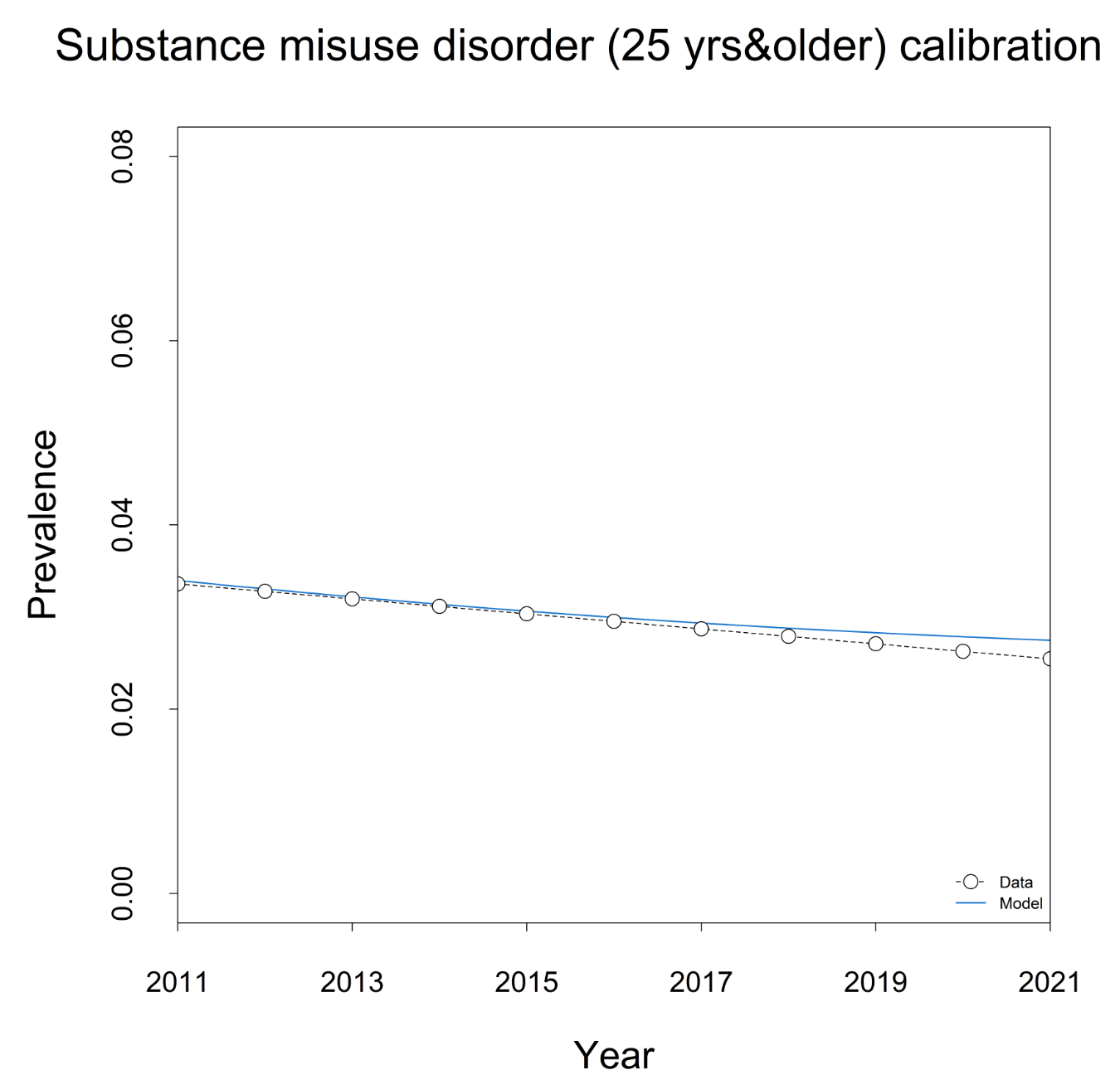


**Figure S21.** Calibration plots from the substance misuse (substance misuse disorder) sector.

## **Substance misuse (substance misuse closed treatment episodes)**

This sector models the flow of people engaging with substance misuse services. People with flow into the substance misuse services waiting stock, representing people on the waitlist for services prior to commencing substance misuse treatment [59]. From the waiting stock, people flow out if they recover without treatment required, if they disengage with services (due to, for example, excessive wait times), through death or, if there are sufficient capacity, through commencing treatment with substance misuse services. From the treatment stock, people flow out if they disengage with services (due to, for example, dissatisfaction with services provided), through death or through the completion of treatment. The remaining flows model ageing, distress / disorder transitions and mortality [55]. This sector was calibrated with substance misuse services data from the AIHW [59].


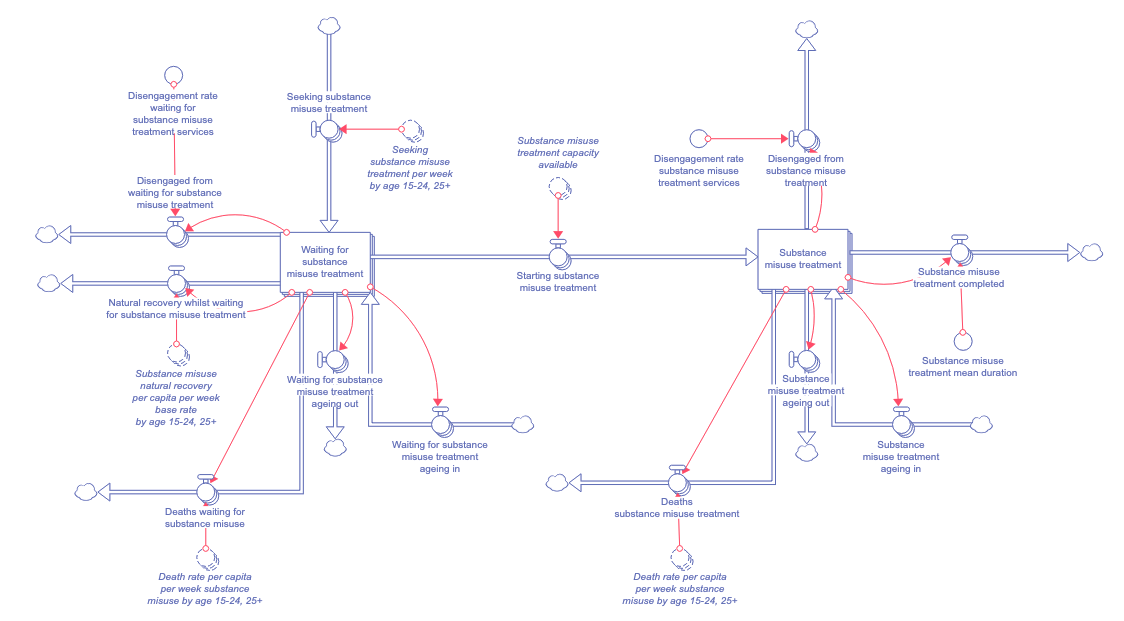


**Figure S22.** Structure of the substance misuse (closed treatment episodes) sector.


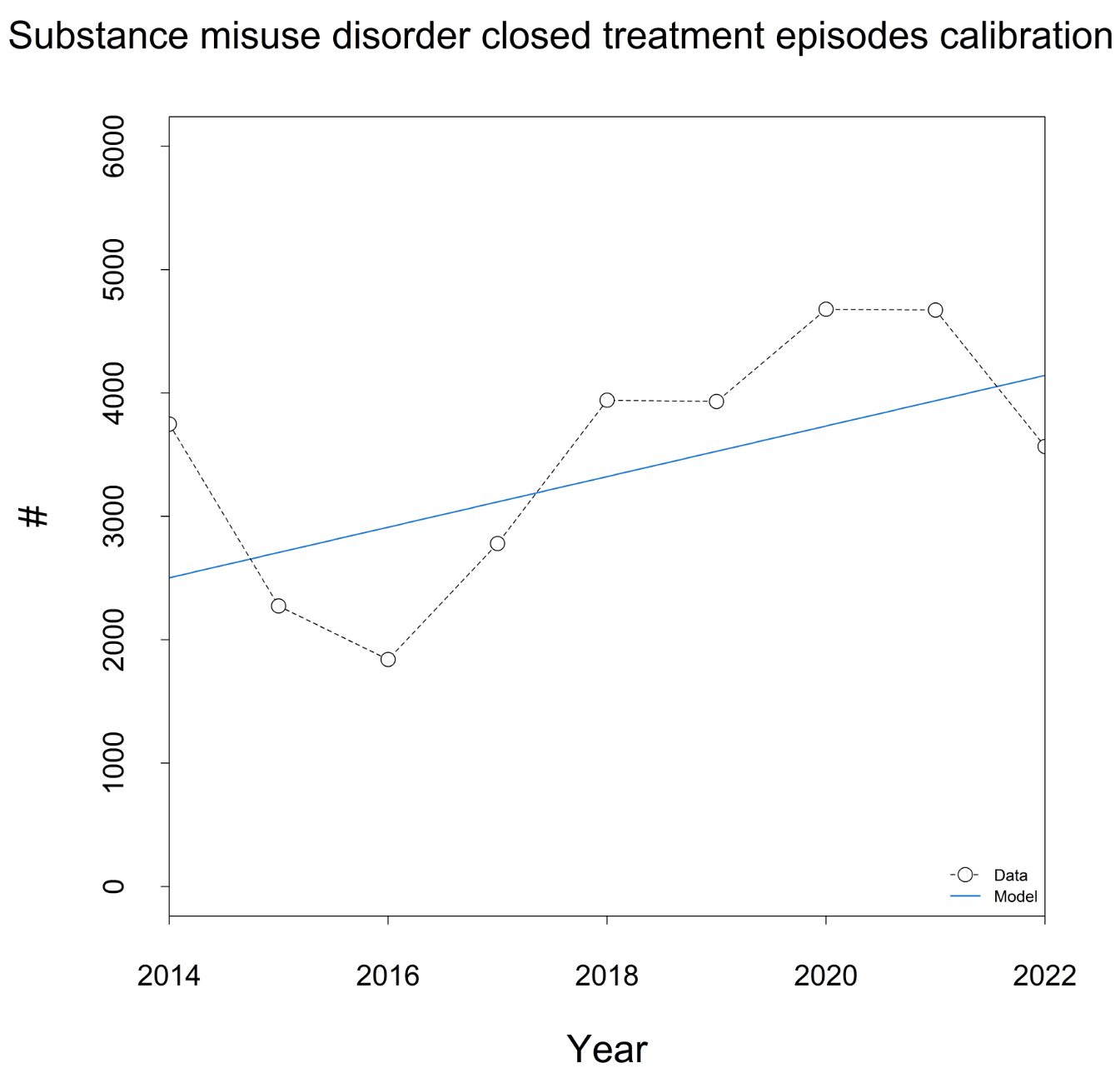


**Figure S23.** Calibration plots from the substance misuse (closed treatment episodes) sector.

## **Suicidal behaviours**

This sector models suicide deaths and attempts in the WSPHN population. The “Suicidal behaviour” stock represents people currently exhibiting suicidal behaviours. People can flow into this stock with an index suicide attempt and people can flow out of this stock depending on whether the attempt was fatal or non-fatal. People who had a non-fatal suicide attempt then flow into the “Recent suicidal behaviour” stock and either remain in this stock for 12 months, representing the duration of which people are at higher risk of exhibiting further suicidal behaviours, or flow back into the “Suicidal behaviours” stock if they have a repeat suicide attempt. The rates of suicide attempts are dependent on age, prevalence of psychological distress / disorder [60] and prevalence of substance misuse disorder [61]. The remaining flows model ageing, distress / disorder transitions and mortality excluding suicide deaths. This sector was calibrated using suicide deaths statistics from the AIHW [62] and HealthStats [63] and intentional self-harm hospitalisations statistics provided by HealthStats [64].

Please note that we calibrated non-fatal suicide attempts with intentional self-harm hospitalisations data. We acknowledge that these data do not fully capture the number of non-fatal suicide attempts (for example, those events not resulting in hospitalisation) and that these data may not accurately record the intention of the event.


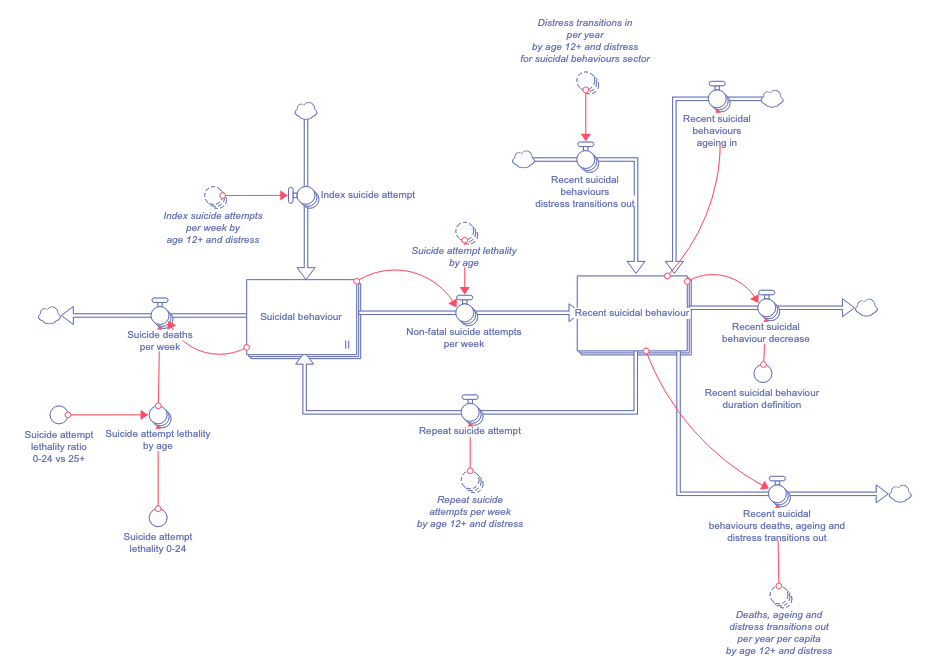


**Figure S24.** Structure of the suicidal behaviours sector.


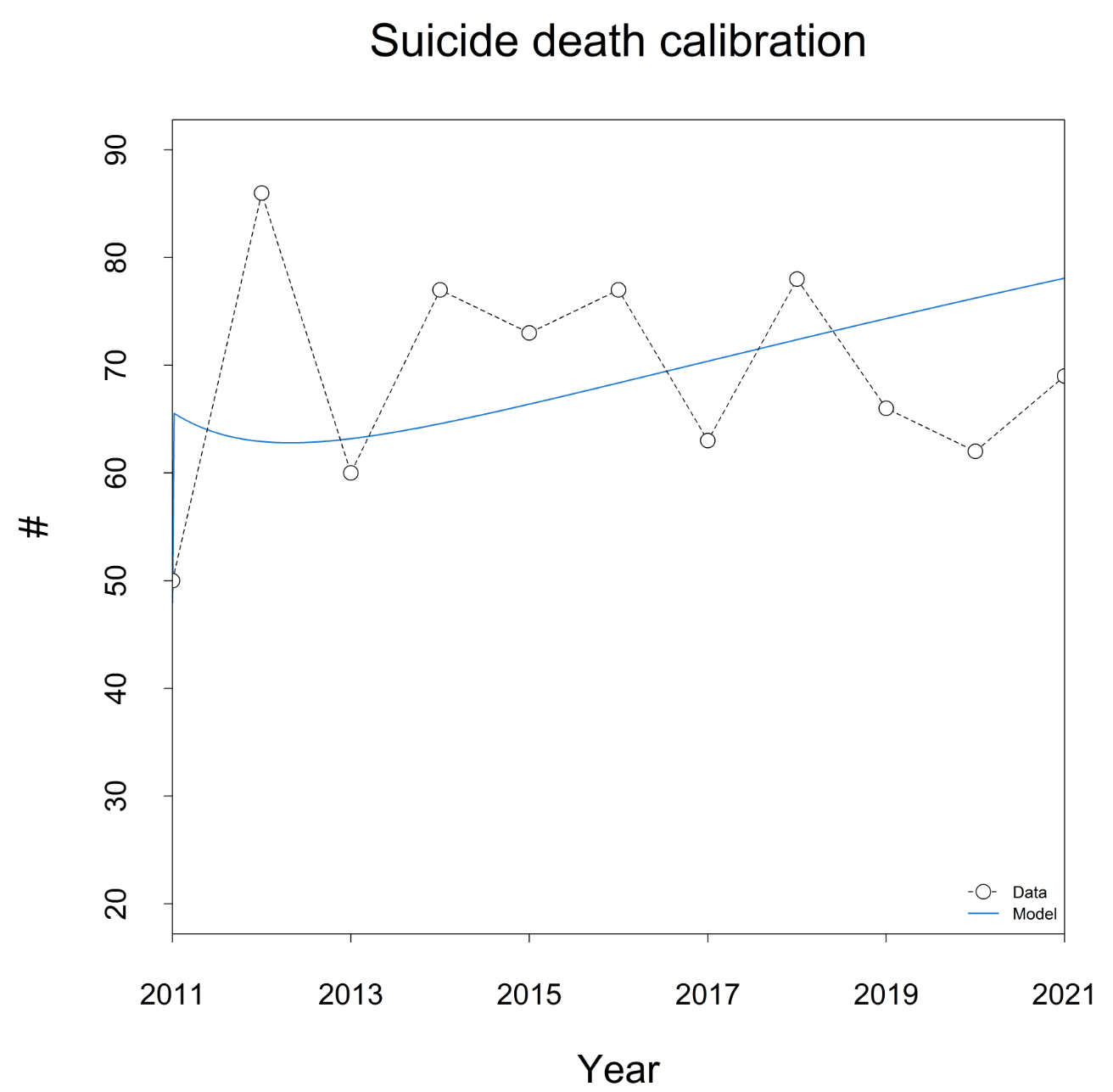

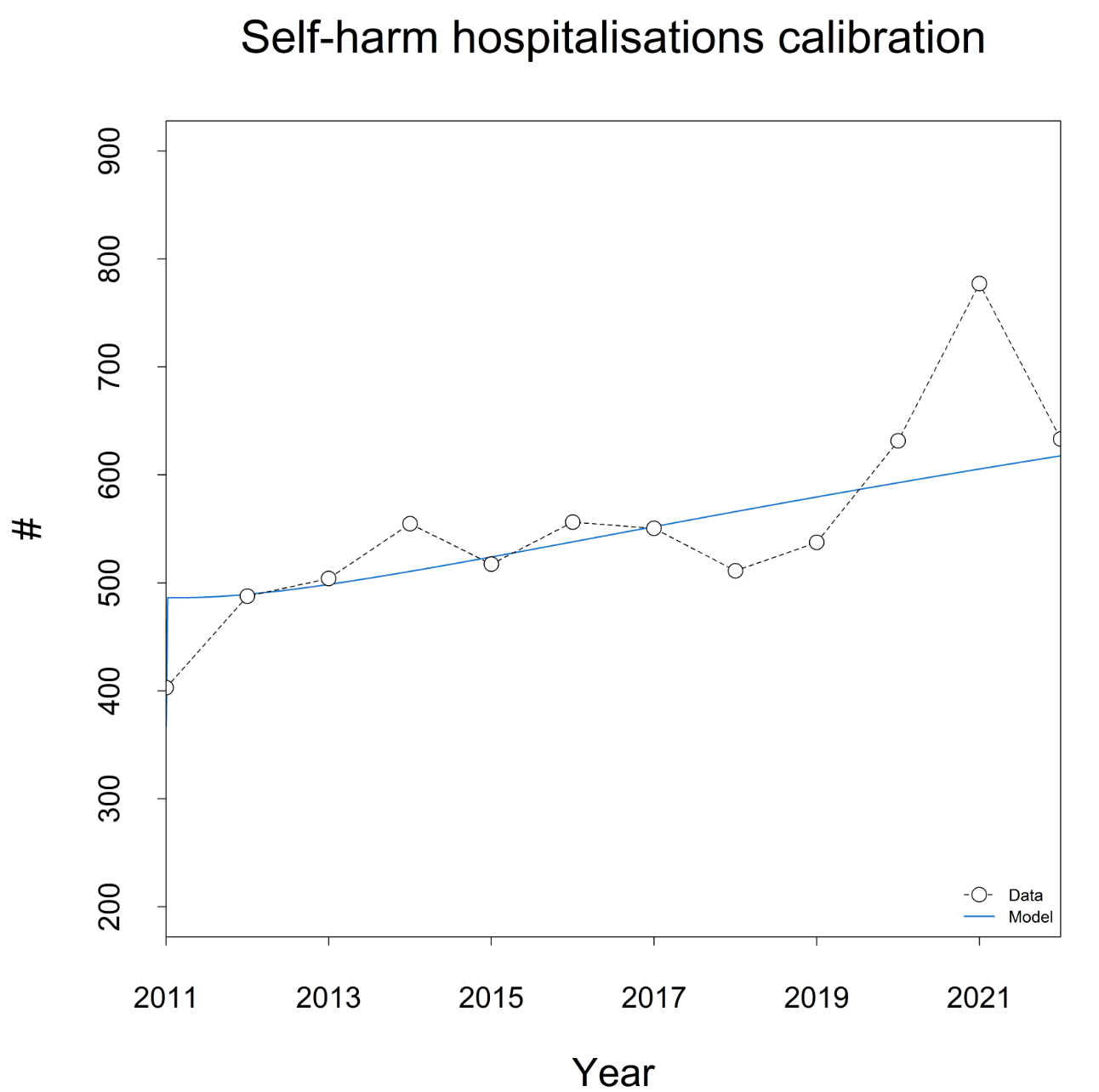


**Figure S25.** Structure of the suicidal behaviours sector.

## **Social cohesion**

This sector models the level of social cohesion in the population. Social cohesion consists of five domains: belonging, worth, social justice, participation and acceptance and rejection. Social cohesion is a stock and flow structure allowing the level of social cohesion to change at a constant rate per year. This sector was calibrated with the Scanlon-Monash Index of Social Cohesion data [65].


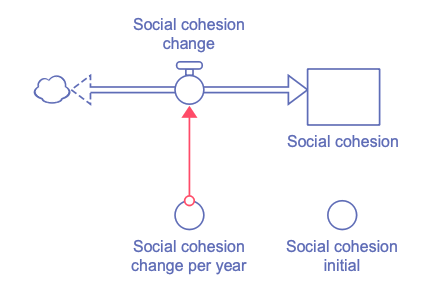


**Figure S26.** Structure of the social cohesion sector.

**
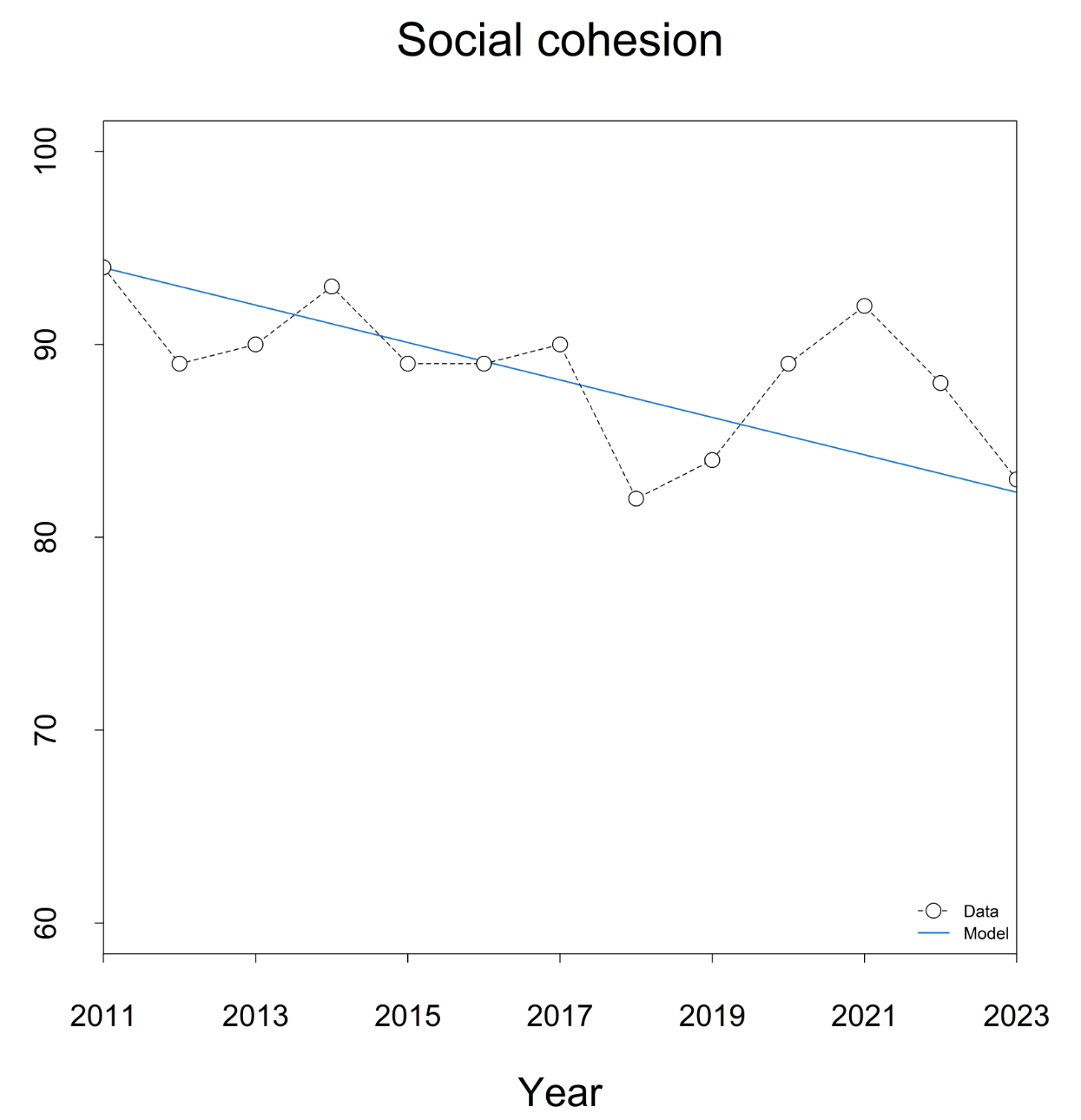
**

**Figure S27.** Calibration plot the social cohesion sector.

## **General Practitioner (GP) services**

This sector models the flow of people engaging with a GP for their mental health. People flow into waiting stock “Waiting for GP”, representing people on the waitlist for GP mental health services. The two inflows into the waiting stock are people in psychological distress who perceive a need for service and commence help-seeking with their GP, and people who have been referred to their GP post-discharge from a mental health related hospitalisation. From the waiting stock, people flow out if they disengage with services (due to, for example, excessive wait times), they present to an emergency department (due to, for example, high levels of distress) or they commence their consult with the GP, if services capacity allows. From the service stock “GP”, people flow out if they disengage with services (due to, for example, dissatisfaction with services provided), they are referred to other mental health services (e.g. online mental health services) or if their consult is completed without further referrals. The remaining flows model ageing, distress / disorder transitions and mortality. This sector was calibrated with Medicare-subsidised GP mental health services data from the AIHW [66] and from data provided by WSPHN [67].


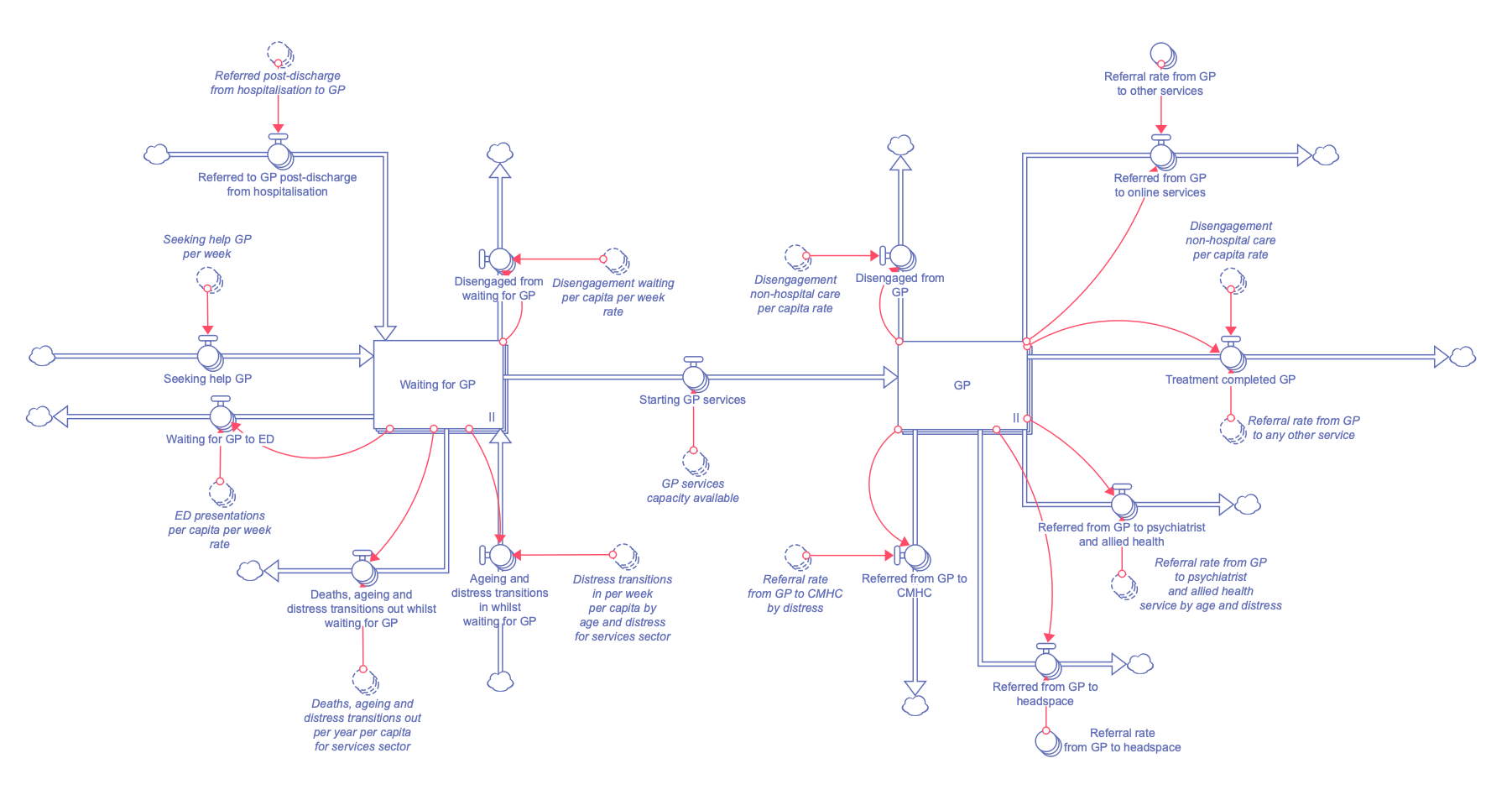


**Figure S28.** Structure of the GP sector.


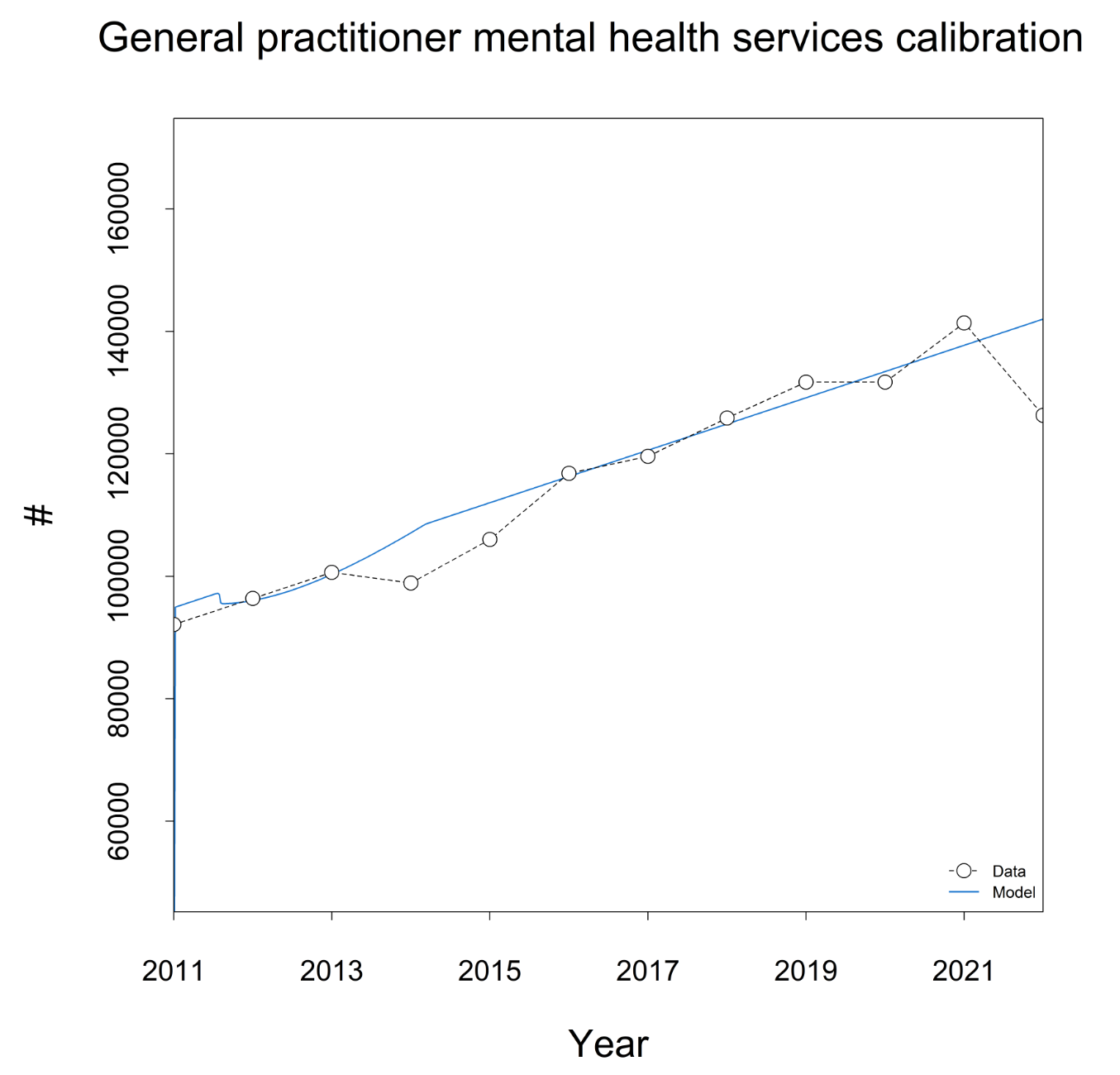


**Figure S29.** Calibration plot from the GP sector.

## **Specialist services**

This sector models the flow of people engaging with specialist services (psychiatrists and allied mental health professionals such as psychologists, social worker and occupational therapists). People flow into the waiting stock “Waiting for psychiatrist and allied health”, representing people on the waitlist for services. The inflows into the waiting stock are people referred by their GP, people referred post-discharge from a mental health related hospitalisation and people with follow-up appointments. From the waiting stock, people flow out if they disengage with services (due to, for example, excessive wait times), they present to an emergency department (due to, for example, high levels of distress) or they commence their consult with specialised services, if services capacity allows. From the service stock “Psychiatrist and allied health”, people flow out if they disengage with services (due to, for example, dissatisfaction with services provided), they are referred to inpatient psychiatric care or if their consult is completed without further referrals. The remaining flows model ageing, distress / disorder transitions and mortality. This sector was calibrated with Medicare-subsidised Psychiatrists and Allied Mental Health mental health services data from the AIHW [66] and from data provided by WSPHN [67].


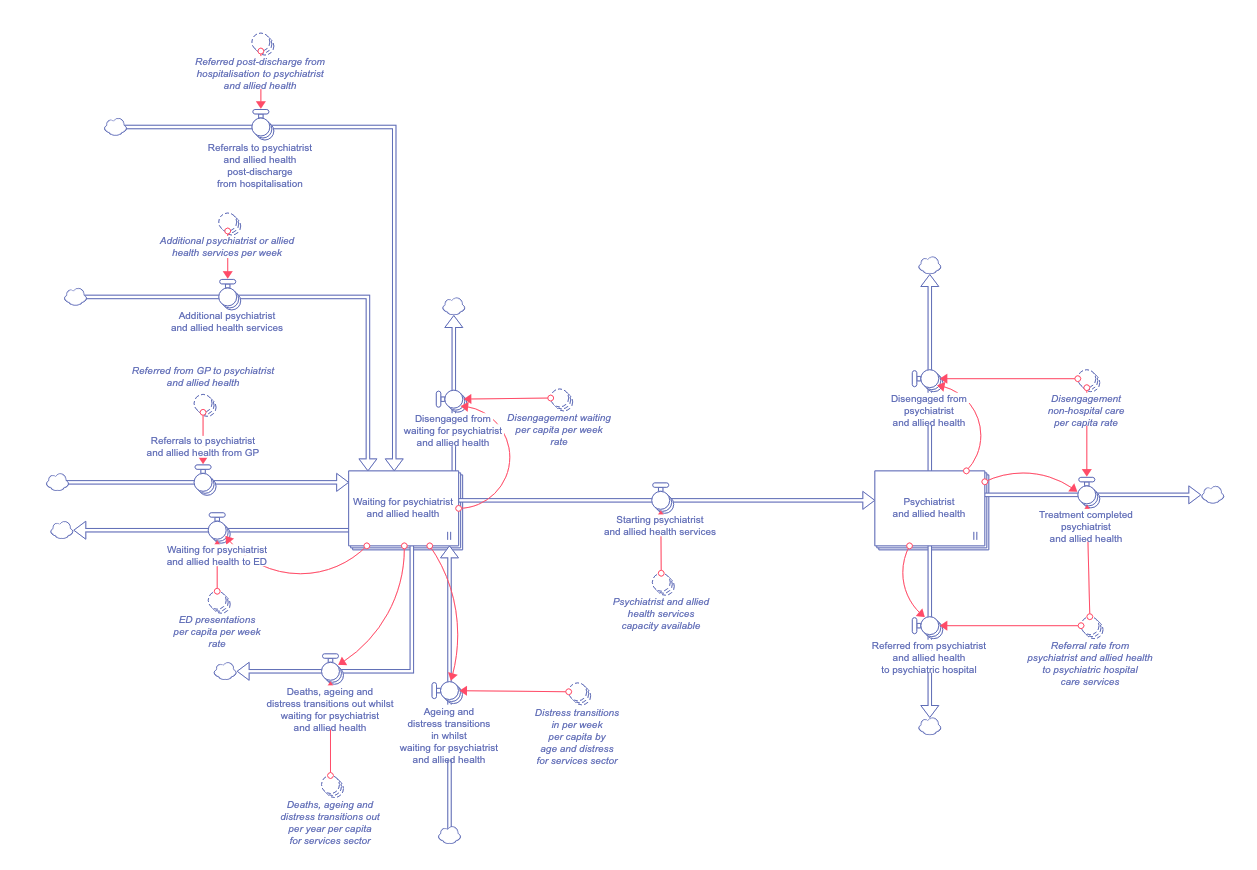


**Figure S30.** Structure of the specialist services sector.

**
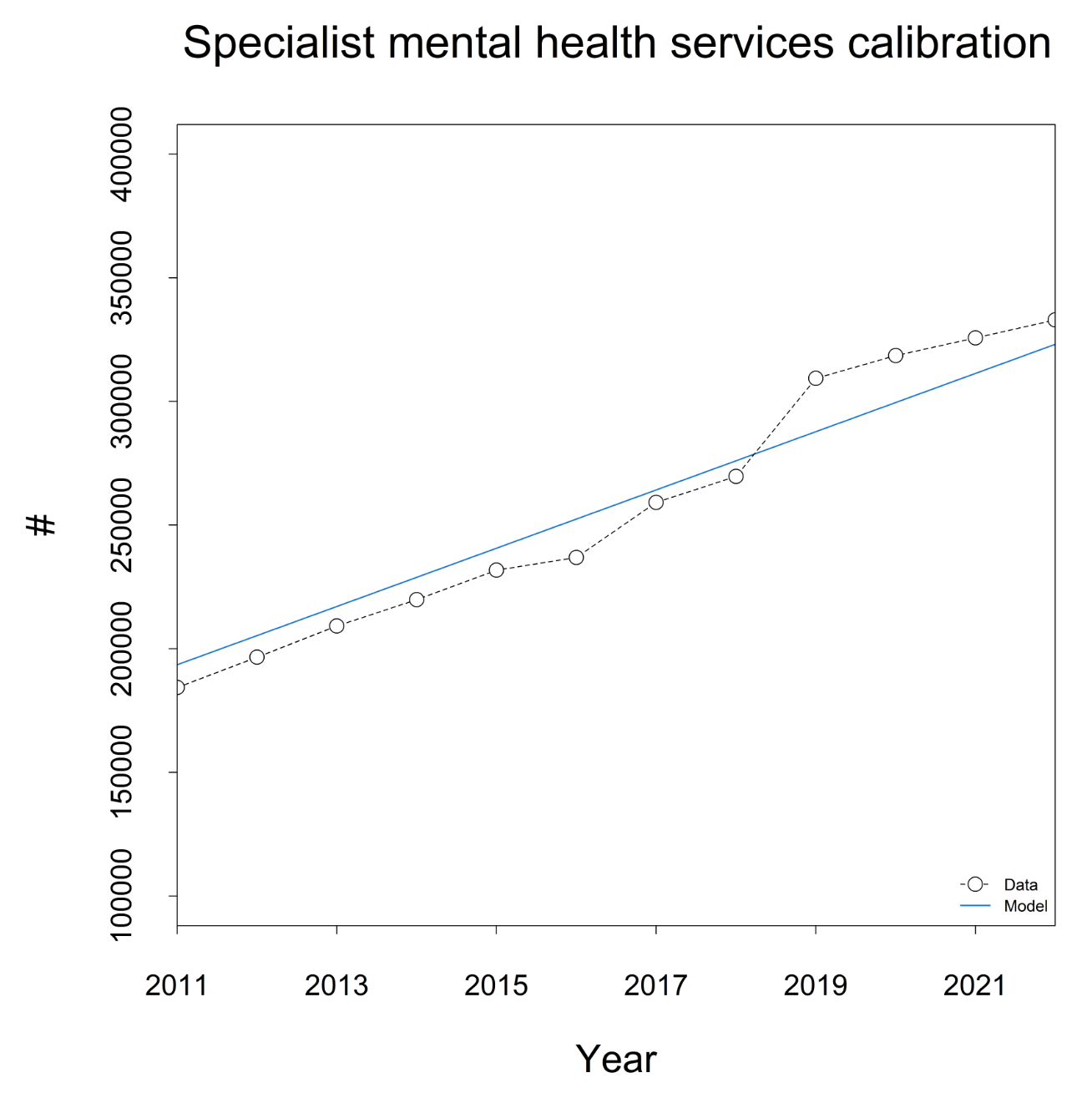
**

**Figure S31.** Calibration plot from the specialist services sector.

## **Community mental health care services**

This sub-sector models the flow of people engaging with child and youth mental health services and community mental health care (CMHC). People flow into the waiting stock “Waiting for CMHC”, representing people on the waitlist for services. The inflows into the waiting stock are people referred by their GP, people referred post-discharge from a mental health related hospitalisation, people referred post-discharge from a mental health related emergency department presentation and people with follow-up appointments. From the waiting stock, people flow out if they disengage with services (due to, for example, excessive wait times), they present to an emergency department (due to, for example, high levels of distress) or they commence their consult with the CYMHS/CMHC, if services capacity allows. From the service stock “CMHC”, people flow out if they disengage with services (due to, for example, dissatisfaction with services provided), or if their consult is completed. The remaining flows model ageing, distress / disorder transitions and mortality. This sector was calibrated with service contacts data from the AIHW [68].


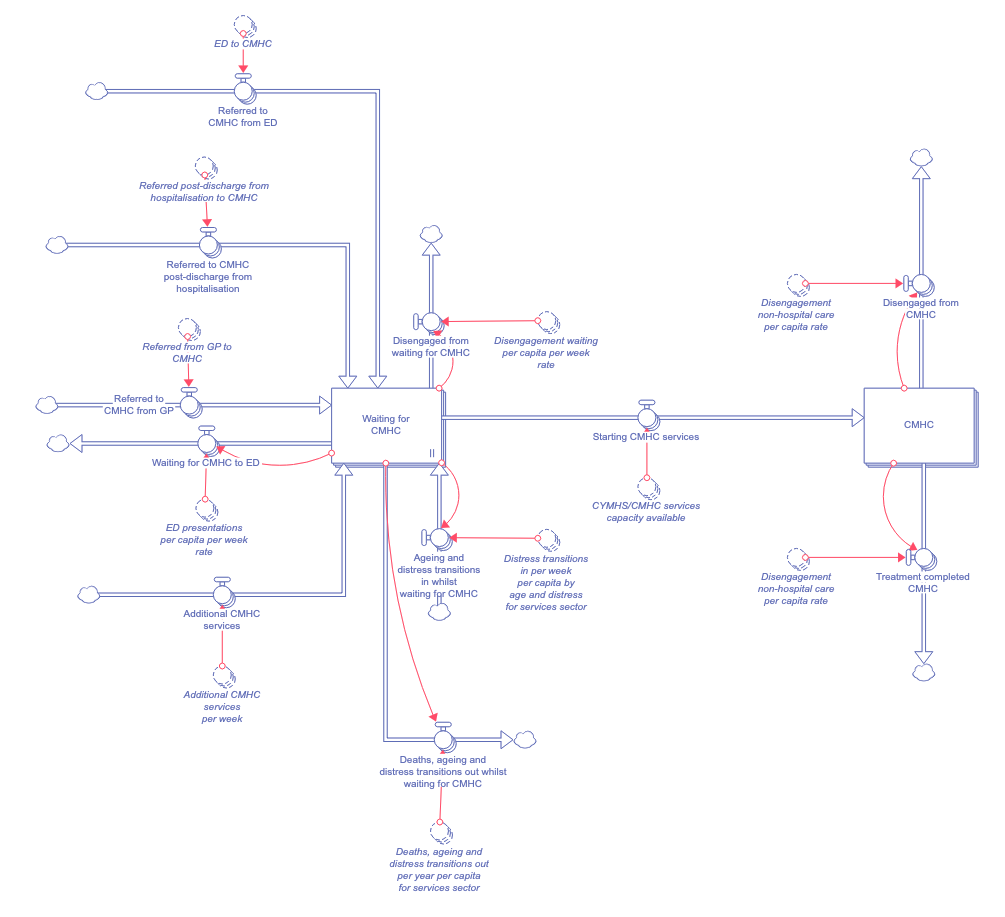


**Figure S32.** Structure of the community mental health care services sector.


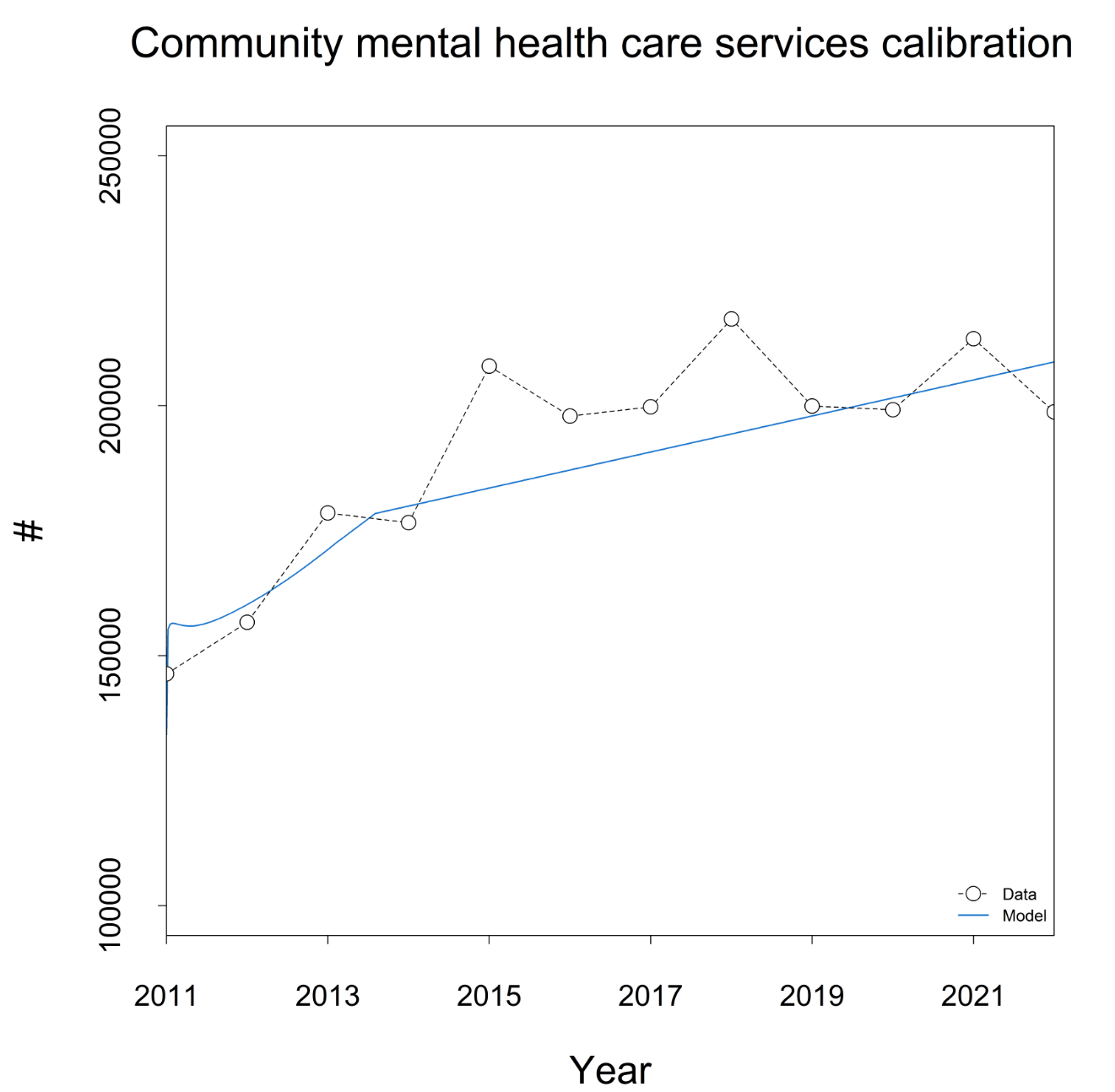


**Figure S33.** Calibration plot from the community mental health care services sector.

## ***headspace* services**

This sector models the flow of young people engaging with youth mental health service provider, headspace. Only people aged 12 and older flow into headspace stocks. People flow into the waiting stock “Waiting for headspace”, representing people on the waitlist for services. The two inflows into the waiting stock are people in psychological distress who perceive a need for service and commence help-seeking with headspace, and people who have been referred by their GP. The first inflow reflects people self-referring to headspace, people referred to headspace by family or friends and people with follow-up appointments. From the waiting stock, people flow out if they disengage with services (due to, for example, excessive wait times), they present to an emergency department (due to, for example, high levels of distress) or they commence their consult with headspace, if services capacity allows. From the service stock “headspace”, people flow out if they disengage with services (due to, for example, dissatisfaction with services provided), or if their consult is completed without further referrals. The remaining flows model ageing, distress / disorder transitions and mortality. This sector was calibrated with occasions of service data provided by WSPHN.


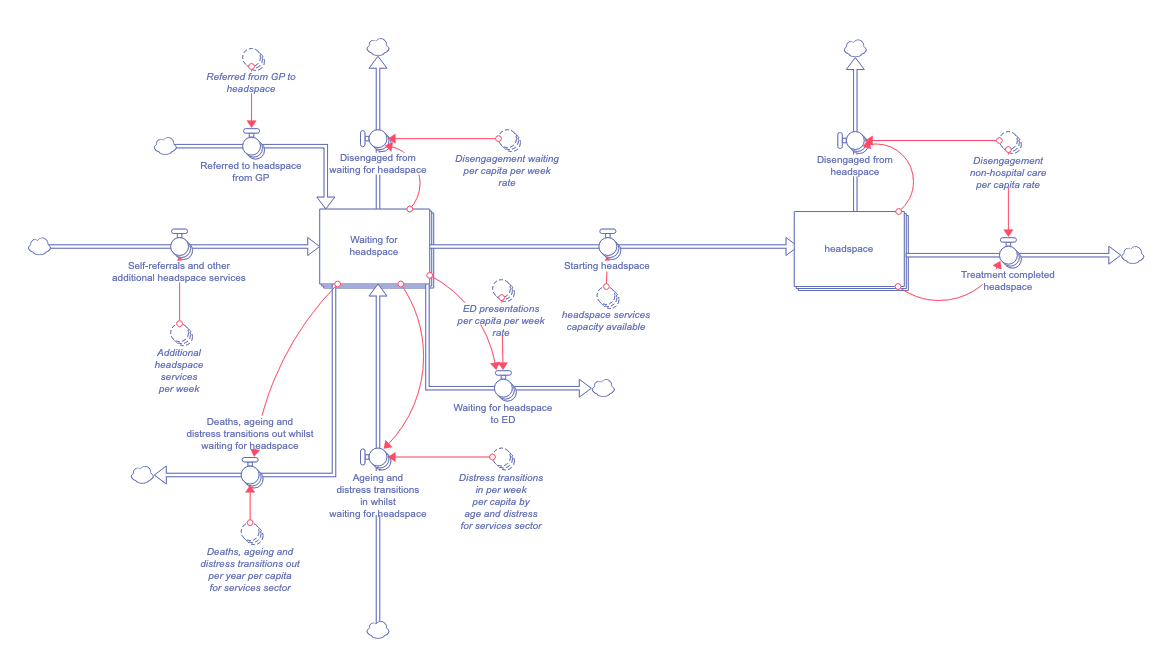


**Figure S34.** Structure of the headspace sector.


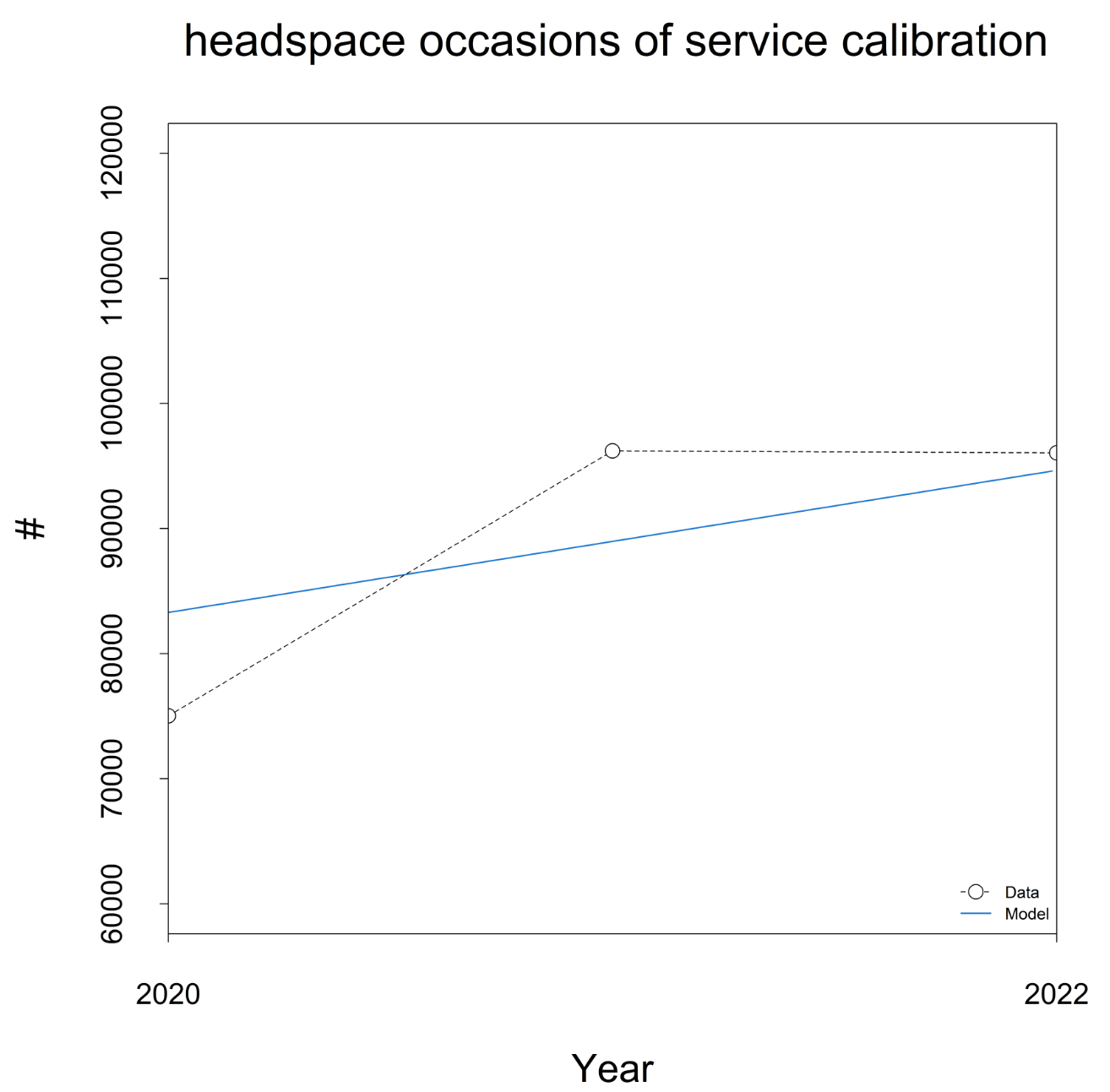


**Figure S35.** Calibration plot from the headspace sector.

## **Online mental health services**

This sector models the flow of people engaging online mental health services. The two inflows into the stock are people in psychological distress who perceive a need for service and commence help-seeking with online services, and people who have been referred by their GP. People flow out when their online course of treatment is completed. The remaining flows model ageing, distress / disorder transitions and mortality.


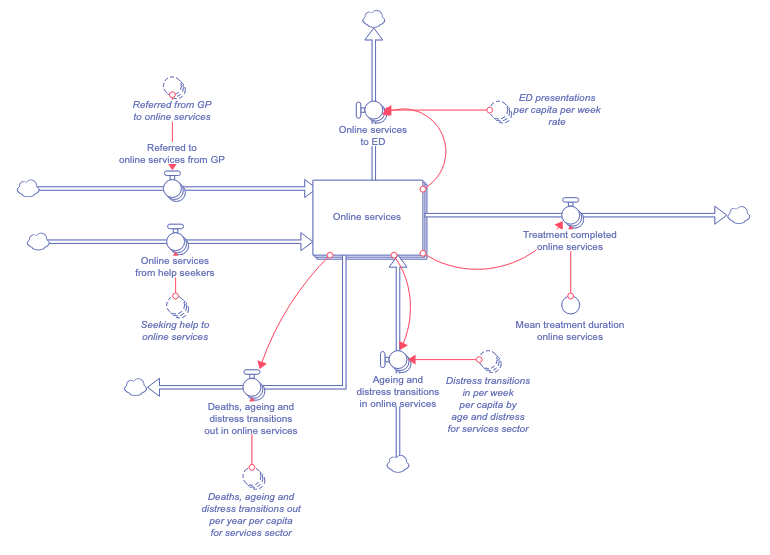


**Figure S36.** Structure of the online mental health services sector.

## **Mental health-related emergency department (ED) presentations**

This sector models people presenting to the ED for mental health related presentations. People that flow into the stock are people who are distressed and are currently help-seeking with other services (e.g., whilst waiting for a consult with a psychologist), are not currently help-seeking (e.g., people whose family or friends take them to ED) and people who are disengaged from services. From the stock, people flow out if they are admitted into either psychiatric admitted care or non-specialised admitted care, discharged and referred to CMHC, or discharged without further referrals. This sector was calibrated with mental health related ED presentations data provided by HealthStats [69].

­
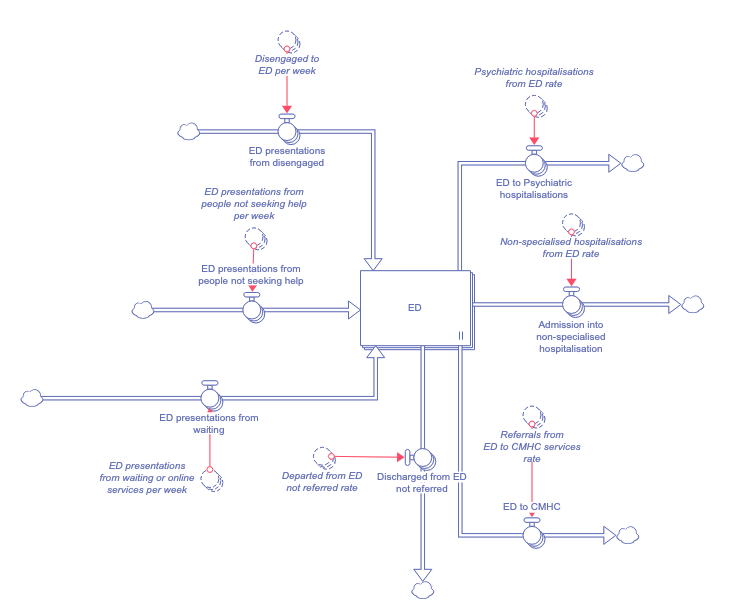


**Figure S37.** Structure of the mental health related ED presentations sector.


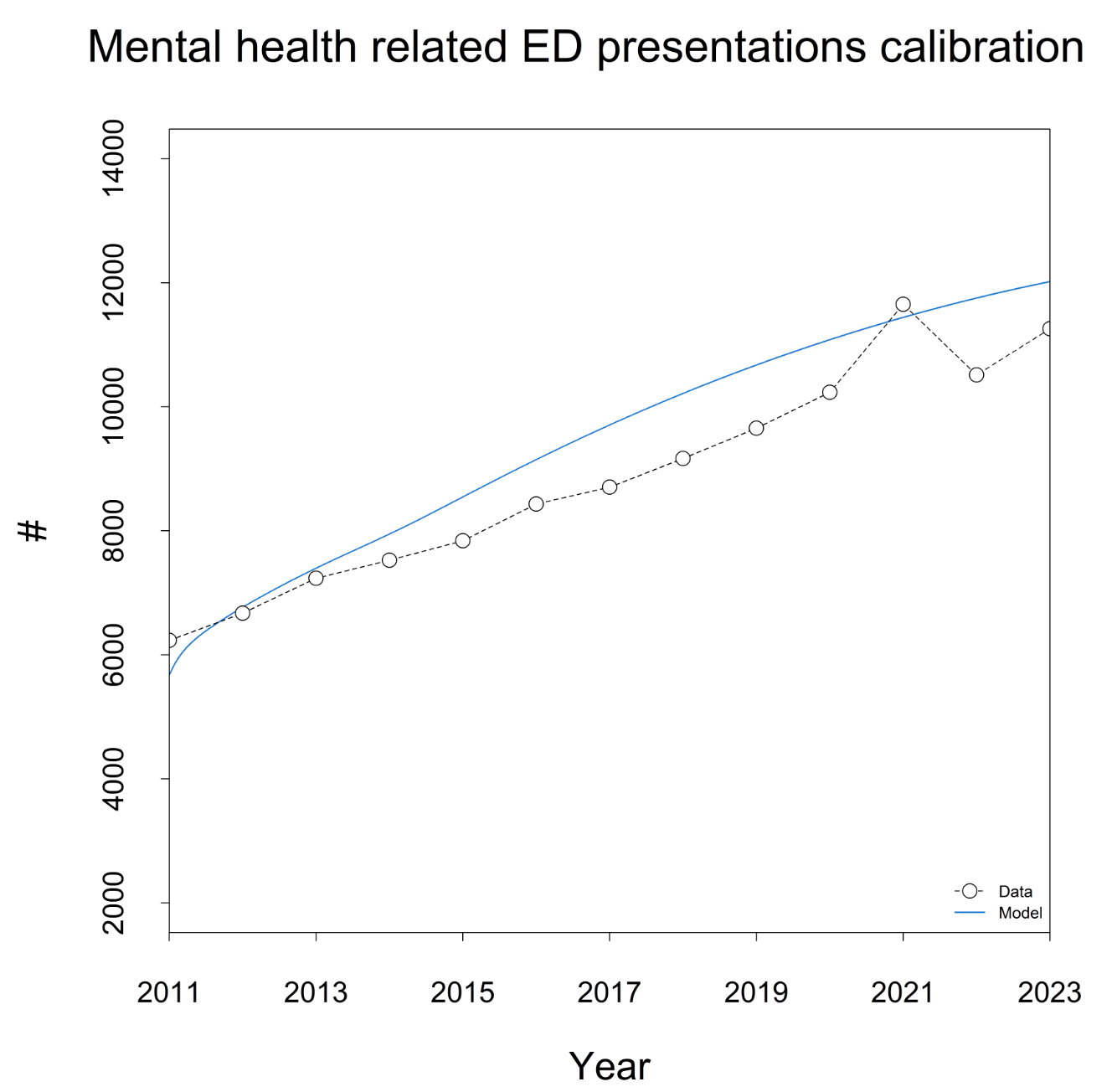


**Figure S38.** Calibration plot from the mental health related ED presentations sector.

## **Psychiatric admitted care**

This sector models the flow of people into psychiatric admitted care. People flow into the waiting stock “Waiting for psychiatric hospitalisation”, representing people on the waitlist for services. The inflow into the waiting stock are people referred by their specialist. From the waiting stock, people flow out if they disengage with services (due to, for example, excessive wait times), they present to an emergency department (due to, for example, high levels of distress) or they commence their psychiatric admitted care, if services capacity allows. People can directly flow into the service stock if the admission is from the ED. From the service stock “Psychiatric hospitalisation”, people flow out once they are discharged. The remaining flows model ageing, distress / disorder transitions and mortality. This sector was calibrated with episodes of admitted care data provided by HealthStats [70].


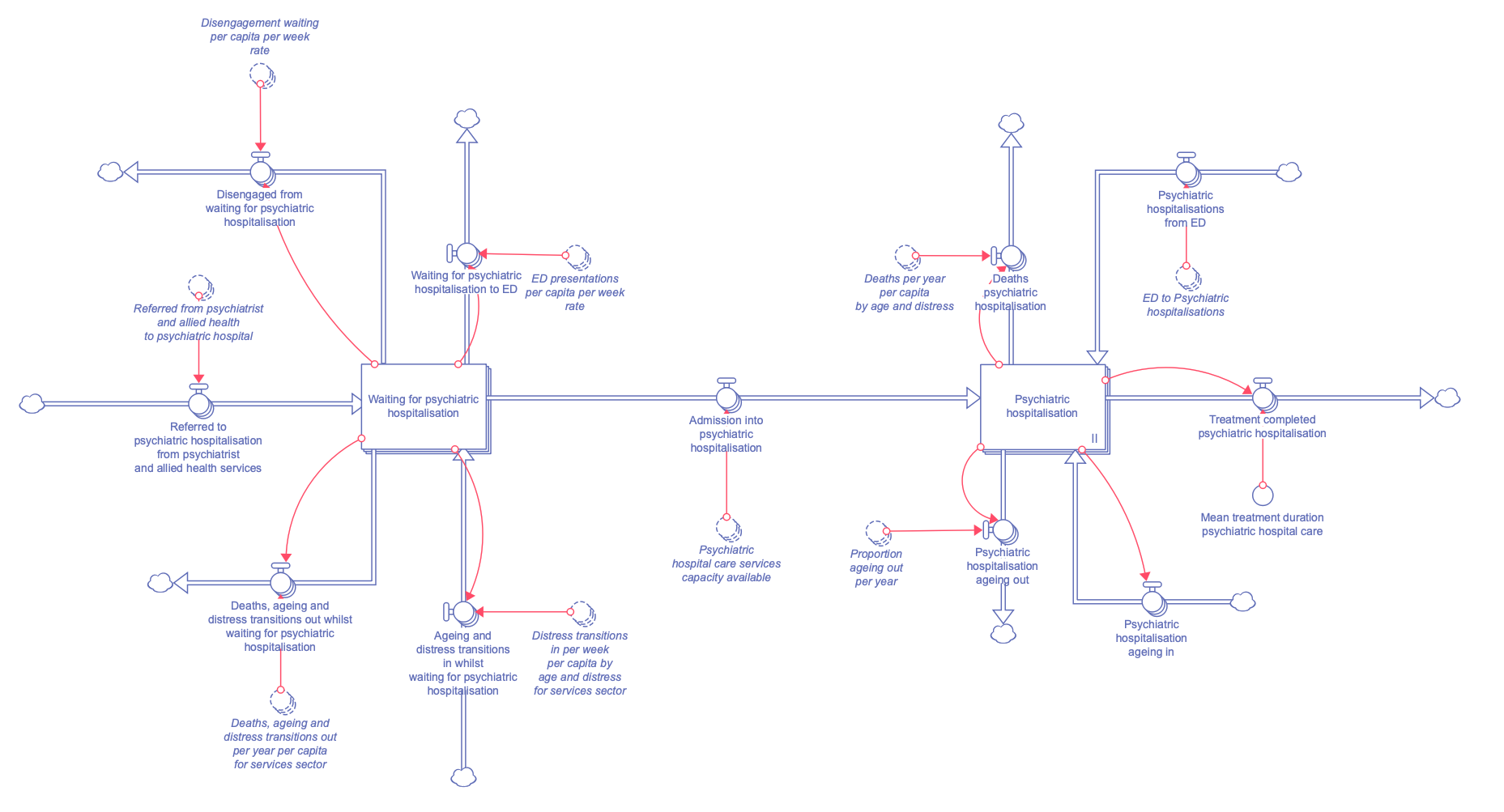


**Figure S39.** Structure of the psychiatric admitted care sector.


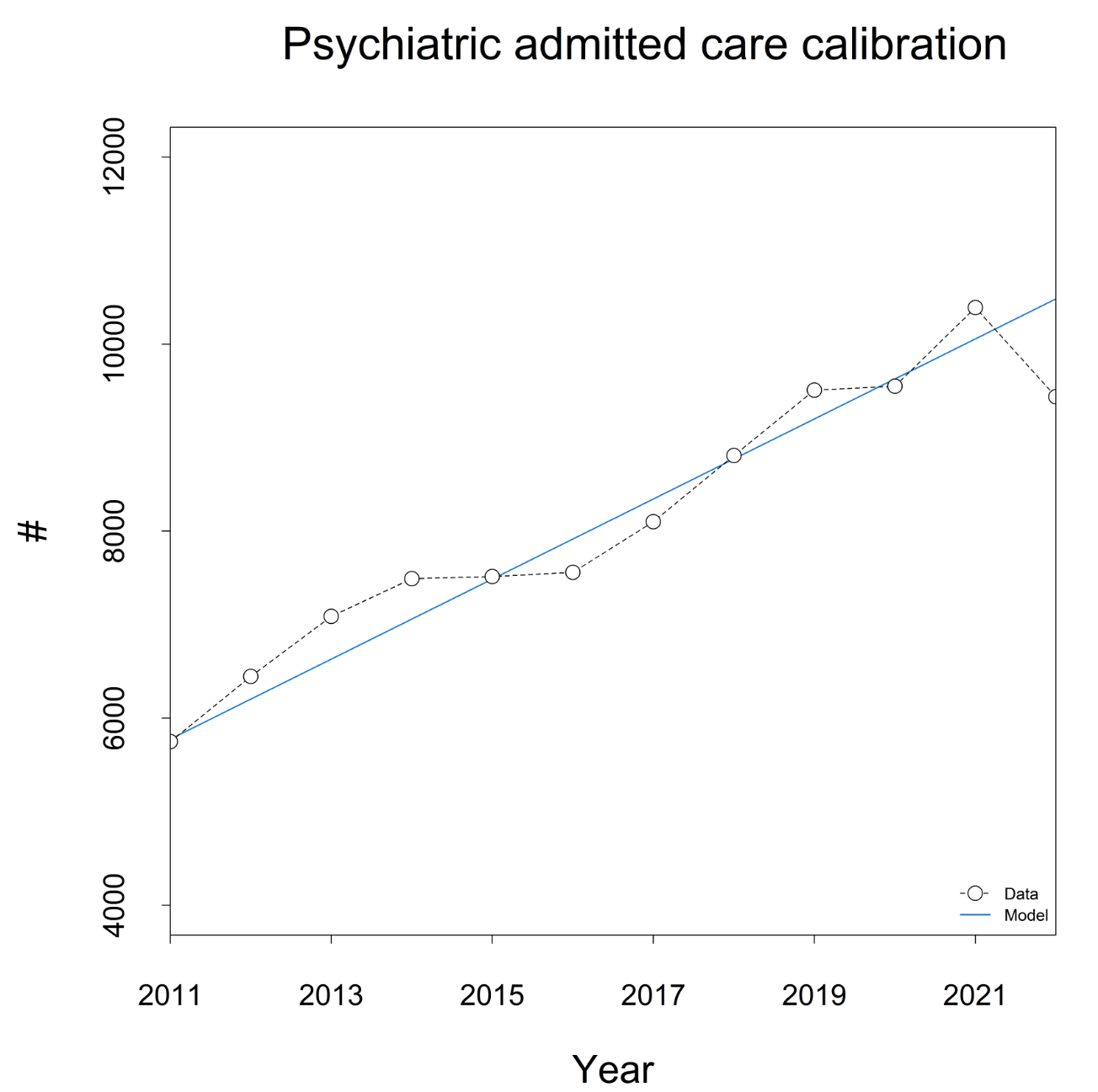


**Figure S40.** Calibration from the psychiatric admitted care sector.

## **Non-specialised admitted care**

This sector models the flow of people into non-specialised, mental health related admitted care. People that flow into the stock are people who are admitted from ED and any other additional admissions. From the service stock, people flow out once they are discharged. The remaining flows model ageing, distress / disorder transitions and mortality. This sector was calibrated with episodes of admitted care data provided by HealthStats [70].


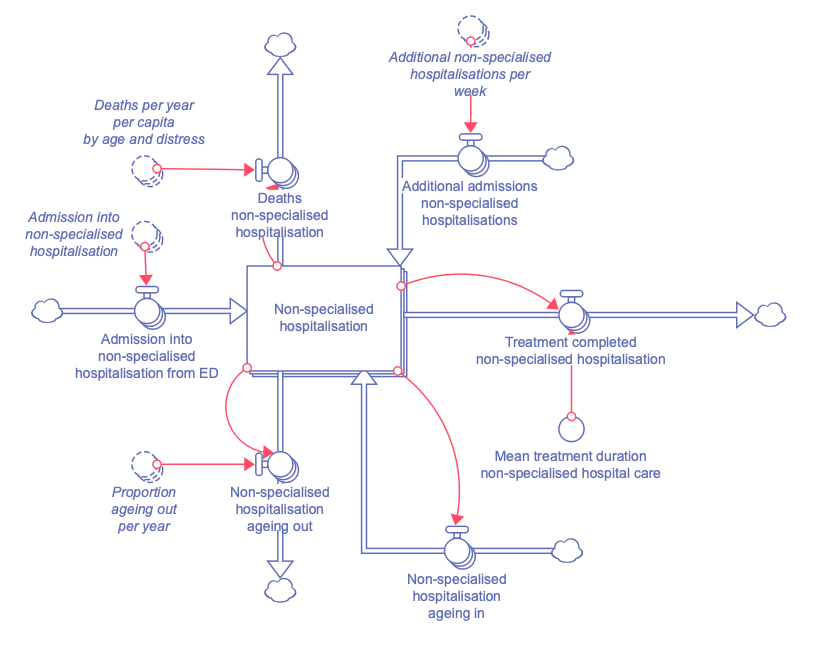


**Figure S41.** Structure of the non-specialised admitted care sector.


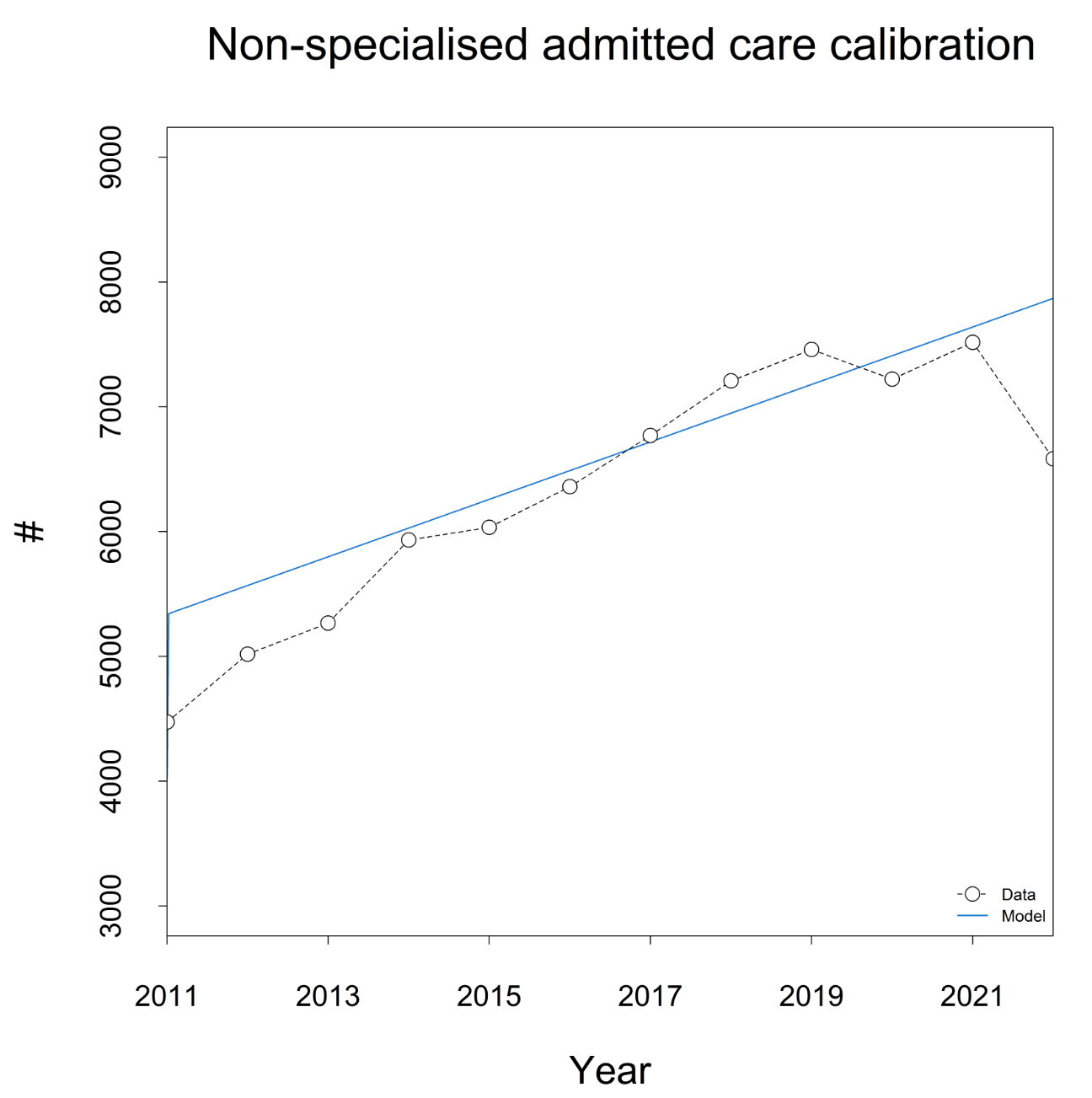


**Figure S42.** Calibration plot from the non-specialised admitted care sector.

## **Help-seeking and disengagement**

This models the flow of people in distress who perceive a need for service and are either seeking help or are disengaged from services. People in distress may develop a perceive need for service and flow into the ”Seeking help” stock. The other inflows are people who present to ED without a perceived need for service (e.g., people whose family or friends take them to ED) and people who were disengaged then re-engaging with services. People who present to ED without a perceived need for service will flow out of the “Seeking help” stock once they are discharged. The flow from “Seeking help” to “Disengaged” models people who disengage from services whilst waiting for services or as a result of poor quality care. Whilst disengaged, people develop psychological disorders at a higher rate than the total population’s per-capita rate. The remaining flows model ageing, distress / disorder transitions and mortality. This sector was calibrated with perceived need for service data from ABS’ National Survey of Mental Health and Wellbeing [71] and the AIHW’s Mental health perfomance indicators [72].


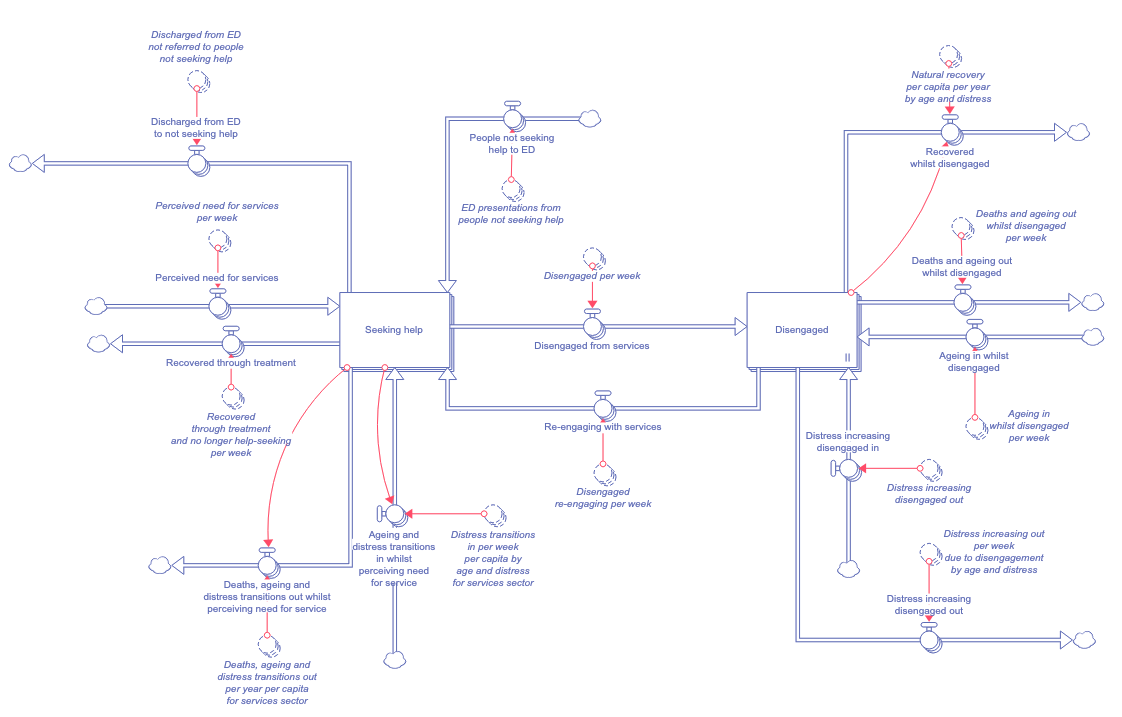


**Figure S43.** Structure of seeking-help and disengaged.

# **Modelling approach for services capacity growth rate**

To reflect a slowing growth rate in services capacity in recent years compared to the longer term historic trend (see Figure 1 in the main paper), multipliers have been applied to annual growth rates for forward projections (i.e., from January 2025). These mulipliers can be modified on the user interface.

**Table S1.** Services capacity growth rate.

| **Service** | **Growth rate** |
| --- | --- |
| General practitioner mental health services | Based on Medicare-subsidised services data published by AIHW for the period 2011 to 2022 [66], the maximum number of GP mental health services that can be delivered per week has been increasing at an annual rate of 61.3 additional services per week (equal to 3.5% average yearly growth). This assumes that services were operating at maximum capacity over this period.  The default value of 0.1 (equal to 0.35% average yearly growth) for the future growth rate multiplier is derived from the latest growth in the available data for recent 3-5 years [66] and represents a decrease in the annual growth rate from January 2025. |
| Specialist mental health services | Based on Medicare-subsidised services data published by AIHW for the period 2011 to 2022 [66], the maximum number of psychiatry and allied mental health services that can be delivered per week has been increasing at an annual rate of 251 additional services per week (equal to 7.1% average yearly growth). This assumes that services were operating at maximum capacity over this period.  The default value for the future growth rate multiplier (equal to 0.45 which gives 3.2% average yearly growth) was derived from Medicare-subsidised services data for recent 3-5 years [66] and represents a decrease in the annual growth rate from January 2025 onwards. |
| Child and youth mental health services | Based on Community Mental Health Care Services published by AIHW for the period 2014 to 2018 [68], the maximum number of child and youth mental health services that can be delivered per week has been increasing at an annual rate of 49.4 additional services per week (equal to 15.6% average yearly growth). This assumes that services were operating at maximum capacity over this period.  The default value for the future growth rate multiplier (0.3 which is equal to 4.7% average yearly growth) was derived from Community Mental Health Care Services data published by AIHW for recent years [68] and represents a decrease in the annual growth rate. |
| Community mental health care services | Based on Community Mental Health Care Services published by AIHW for the period 2014 to 2018 [68], the maximum number of community mental health care services that can be delivered per week has been increasing at an annual rate of 146 additional services per week (equal to 5.9% average yearly growth). This assumes that services were operating at maximum capacity over this period.  The default value for the future growth rate multiplier (0.05 which is equal to 0.29% average yearly growth) was derived from Community Mental Health Care Services data published by AIHW for recent years [68] and represents a decrease in the annual growth rate. |
| headspace | Based on headspace occasions of service data provided by WSPHN for the period 2020 to 2022, the maximum number of headspace services that can be delivered per week has been increasing at an annual rate of 172.3 additional services per week (equal to 34.9% average yearly growth). This assumes that services were operating at maximum capacity over this period.  The default value for the future growth rate multiplier (0.95 which is equal to 33.2% average yearly growth) was derived from headspace occasions of service data provided by WSPHN for recent years and represents a decrease in the annual growth rate. |
| Psychiatric admitted care | Based on episodes of admitted patient care provided by NSW HealthStats [70,73] for the period 2011 to 2022, the maximum number of episodes of admitted patient care that can be delivered per week has been increasing at an annual rate of 5.5 additional episodes per week (equal to 3.95% average yearly growth). This assumes that services were operating at maximum capacity over this period.  The default value for the future growth rate multiplier (0.34 which is equal to 1.34% average yearly growth) was derived from episodes of admitted patient care provided by NSW HealthStats [70,73] for recent 3-5 years and represents a decrease in the annual growth rate. |
| Substance misuse treatment services | Based on alcohol and other drug closed treatment episodes published by AIHW [59] for the period 2013-14 to 2021-2022, the maximum number of closed treatment episodes that can be delivered per week has been increasing at an annual rate of 2.5 additional episodes per week (equal to 7.6% average yearly growth). This assumes that services were operating at maximum capacity over this period.  The default value for the future growth rate multiplier (0.73 which is equal to 5.6% average yearly growth) was derived from alcohol and other drug closed treatment episodes published by AIHW [59] for recent 3-5 years and represents a decrease in the annual growth rate. |

# **Numerical inputs**

**Table S2.** Numerical inputs.

| **Variable Name** | **Stratification / Value** | | | **Notes** |  |
| --- | --- | --- | --- | --- | --- |
| **Population** | | | | |  |
| Population initial by age | 0–4 years | | 64530 | Centre for Epidemiology and Evidence [3] |  |
|  | 5–11 years | | 80084.2 |  |  |
|  | 12-14 years | | 33481.8 |  |  |
|  | 15-17 years | | 33832.8 |  |  |
|  | 18-24 years | | 86571.2 |  |  |
|  | 25 years and older | | 547353 |  |  |
| Birth rate per year initial | 0.01715073423 | | | The initial values sourced from the Centre for Epidemiology and Evidence [4], was calibrated using constrained optimisation. |  |
| Birth rate increase per year | -0.00028417411 | | |  |  |
| Deaths per capita per year initial | 0–4 years | | 0.00079964524 | The initial values sourced from the Centre for Epidemiology and Evidence [2], was calibrated using constrained optimisation. |  |
|  | 5–11 years | | 0.00007092512 |  |  |
|  | 12-14 years | | 0.00009777343 |  |  |
|  | 15-17 years | | 0.00029255689 |  |  |
|  | 18-24 years | | 0.00030103047 |  |  |
|  | 25 years and older | | 0.00828052478 |  |  |
| Deaths per capita increase per year | 0–4 years | | -0.00002181788 |  |  |
|  | 5–11 years | | -0.00000117865 |  |  |
|  | 12-14 years | | -0.00000137787 |  |  |
|  | 15-17 years | | -0.00000276186 |  |  |
|  | 18-24 years | | -0.00000428724 |  |  |
|  | 25 years and older | | -0.00007728685 |  |  |
| Overseas arrival by age initial | 0–4 years | | 1135.742838 | The initial values sourced from the Australian Bureau of Statistics [8], was calibrated using constrained optimisation. |  |
|  | 5–11 years | | 1142.658741 |  |  |
|  | 12-14 years | | 667.004177 |  |  |
|  | 15-17 years | | 4174.660387 |  |  |
|  | 18-24 years | | 7295.150298 |  |  |
|  | 25 years and older | | 16432.17987 |  |  |
| Overseas arrival increase per year | 0–4 years | | 1.244834 |  |  |
|  | 5–11 years | | 0.684263 |  |  |
|  | 12-14 years | | 15.731185 |  |  |
|  | 15-17 years | | 0 |  |  |
|  | 18-24 years | | 0 |  |  |
|  | 25 years and older | | 0 |  |  |
| Interstate arrival by age initial | 0–4 years | | 8669.689554 | The initial values sourced from the Australian Bureau of Statistics [6], was calibrated using constrained optimisation. |  |
|  | 5–11 years | | 5245.088207 |  |  |
|  | 12-14 years | | 4915.331988 |  |  |
|  | 15-17 years | | 5082.715596 |  |  |
|  | 18-24 years | | 14695.407338 |  |  |
|  | 25 years and older | | 40516.424751 |  |  |
| Interstate arrival increase per year | 0–4 years | | 0.17495 |  |  |
|  | 5–11 years | | 0.391312 |  |  |
|  | 12-14 years | | 1.652906 |  |  |
|  | 15-17 years | | 119.510858 |  |  |
|  | 18-24 years | | 176.060281 |  |  |
|  | 25 years and older | | 2579.668435 |  |  |
| Per capita overseas departure rate by age initial | 0–4 years | | 0.012623 | The initial values sourced from the Australian Bureau of Statistics [8], was calibrated using constrained optimisation. |  |
|  | 5–11 years | | 0.00608 |  |  |
|  | 12-14 years | | 0.010591 |  |  |
|  | 15-17 years | | 0.017261 |  |  |
|  | 18-24 years | | 0.038085 |  |  |
|  | 25 years and older | | 0.014119 |  |  |
| Per capita overseas departure rate by age increase per year | 0–4 years | | 0.00000360698 |  |  |
|  | 5–11 years | | 0.00001090211 |  |  |
|  | 12-14 years | | 0.00002254343 |  |  |
|  | 15-17 years | | 0.00007802670 |  |  |
|  | 18-24 years | | 0 |  |  |
|  | 25 years and older | | 0 |  |  |
| Per capita interstate departure rate by age initial | 0–4 years | | 0.118972 | The initial values sourced from the Australian Bureau of Statistics [6], was calibrated using constrained optimisation. |  |
|  | 5–11 years | | 0.050981 |  |  |
|  | 12-14 years | | 0.174219 |  |  |
|  | 15-17 years | | 0.242705 |  |  |
|  | 18-24 years | | 0.177192 |  |  |
|  | 25 years and older | | 0.076628 |  |  |
| Per capita interstate departure rate by age increase per year | 0–4 years | | 0.00007879851 |  |  |
|  | 5–11 years | | 0.00000000000 |  |  |
|  | 12-14 years | | 0.00002246297 |  |  |
|  | 15-17 years | | 0.00048343893 |  |  |
|  | 18-24 years | | 0.00000092931 |  |  |
|  | 25 years and older | | 0.00238804905 |  |  |
| **Education - Students** | | | | |  |
| Studying primary education initial | 80680.0 | | | Australian Curriculum Assessment and Reporting Authority [11] |  |
| Studying post-secondary education initial | 58551.0 | | |  |  |
| Studying post-secondary education initial | 70298.8 | | | Estimated with constrained optimisation |  |
| Proportion of population not currently studying entering secondary education per year | 0.00000140889 | | | Estimated with constrained optimisation |  |
| Proportion of population not currently studying entering post-secondary education per year | 0.04369369037 | | | Estimated with constrained optimisation |  |
| Discontinuing secondary study base rate | 0.03853925721 | | | Estimated with constrained optimisation |  |
| Discontinuing post-secondary study base rate | 0.07377998564 | | | Estimated with constrained optimisation |  |
| Completing secondary study rate | 0.13592572251 | | | Estimated with constrained optimisation |  |
| Completing post-secondary study rate | 0.40726356288 | | | Estimated with constrained optimisation |  |
| Proportion of secondary study completers transitioning to post-secondary education | 0.578 | | | Estimated with constrained optimisation |  |
| Prevalence of moderate to very high psychological distress ratio secondary students vs population | 1 | | | Assumes that the prevalence of psychological distress in secondary students is the same as population prevalence for 12-17 year olds. |  |
| Effect of moderate to very high distress on discontinuation of secondary education | 1.99 | | | Butterworth and Leach [9] |  |
| Prevalence of moderate to very high psychological distress ratio post-secondary students vs population | 1.383624 | | | Australian Bureau of Statistics [34] |  |
| Effect of psychological distress on discontinuation of post-secondary education | 1.1 | | | Lee et al [10] |  |
| **Education – Highest qualifications** | | | | |  |
| Proportion of population with secondary qualification only initial by age 15-24, 25+ | 15-24 years | | 0.524843 | Estimated with constrained optimisation |  |
|  | 25 years and older | | 0.134588 |  |  |
| Proportion of population with post-secondary qualification initial by age 15-24, 25+ | 15-24 years | | 0.200518 | Estimated with constrained optimisation |  |
|  | 25 years and older | | 0.511273 |  |  |
| Proportion completing first post-secondary qualification by age 15-24, 25+ | 15-24 years | | 0.669846 | Estimated with constrained optimisation |  |
|  | 25 years and older | | 0.209593 |  |  |
| Proportion of secondary school graduates who are aged 15-24 | 0.68763 | | | Estimated with constrained optimisation |  |
| Proportion of post-secondary graduates who are aged 15-24 | 0.058042 | | | Estimated with constrained optimisation |  |
| Death rate ratio post-secondary qualification vs low education | 0.3623188 | | | Welsh et al [15] |  |
| Death rate ratio secondary qualification only vs low education | 0.635 | | | Welsh et al [15] |  |
| Proportion of population with secondary qualification only ratio Australia vs WSPHN | 15-24 years | | 0.930905 | The base value obtained from the Australian Bureau of Statistics [74] and calibrated with constrained optimisation. |  |
|  | 25 years and older | | 1.082437 |  |  |
| Proportion of population with post-secondary qualification ratio Australia vs WSPHN | 15-24 years | | 1.212353 |  |  |
|  | 25 years and older | | 1.069558 |  |  |
| **Labour force** | | | | |  |
| Proportion of population 15-24, 25+ sufficiently employed proportion initial | 15-24 years | | 0.491568 | Estimated with constrained optimisation |  |
|  | 25 years and older | | 0.565302 |  |  |
| Proportion of population 15-24, 25+ underemployed proportion initial | 15-24 years | | 0.066089 | Estimated with constrained optimisation |  |
|  | 25 years and older | | 0.033498 |  |  |
| Proportion of population 15-24, 25+ unemployed proportion initial | 15-24 years | | 0.072357 | Estimated with constrained optimisation |  |
|  | 25 years and older | | 0.023882 |  |  |
| Sufficiently employed to unemployed per capita per year base rate by age 15-24, 25+ | 15-24 years | | 0.050947 | Estimated with constrained optimisation |  |
|  | 25 years and older | | 0.023686 |  |  |
| Unemployed to sufficiently employed per capita per year base rate by age 15-24, 25+ | 15-24 years | | 0.768632 | Estimated with constrained optimisation |  |
|  | 25 years and older | | 1.039768 |  |  |
| Underemployed to unemployed per capita per year base rate by age 15-24, 25+ | 15-24 years | | 0.275080 | Estimated with constrained optimisation |  |
|  | 25 years and older | | 0.153372 |  |  |
| Unemployed to underemployed per capita per year base rate by age 15-24, 25+ | 15-24 years | | 0.832586 | Estimated with constrained optimisation |  |
|  | 25 years and older | | 0.623461 |  |  |
| Sufficiently employed to underemployed per capita per year base rate by age 15-24, 25+ | 15-24 years | | 0.381244 | Estimated with constrained optimisation |  |
|  | 25 years and older | | 0.203852 |  |  |
| Underemployed to sufficiently employed per capita per year base rate by age 15-24, 25+ | 15-24 years | | 1.323538 | Estimated with constrained optimisation |  |
|  | 25 years and older | | 1.965370 |  |  |
| Unemployed to NILF per capita per year base rate by age 15-24, 25+ | 15-24 years | | 3.351604 | Estimated with constrained optimisation |  |
|  | 25 years and older | | 2.966020 |  |  |
| NILF to Unemployed per capita per year base rate by age 15-24, 25+ | 15-24 years | | 0.975693 | Estimated with constrained optimisation |  |
|  | 25 years and older | | 0.292677 |  |  |
| Sufficiently employed to NILF per capita per year base rate by age 15-24, 25+ | 15-24 years | | 0.059822 | Estimated with constrained optimisation |  |
|  | 25 years and older | | 0.076708 |  |  |
| Underemployed to NILF per capita per year base rate by age 15-24, 25+ | 15-24 years | | 1.003573 | Estimated with constrained optimisation |  |
|  | 25 years and older | | 0.748079 |  |  |
| Proportion of population sufficiently employed ratio: rest of Australia vs NSW | 1.697679 | | | Australian Bureau of Statistics [22] |  |
| Proportion of population underemployed ratio: rest of Australia vs NSW | 0.842160 | | | Australian Bureau of Statistics [22] |  |
| Proportion of population unemployed ratio: rest of Australia vs WSPHN | 0.960593 | | | Australian Bureau of Statistics [74] |  |
| Death rate ratio: unemployed vs employed | 1.22 | | | Sorlie and Rogot [16] |  |
| Effect of post-secondary qualification on underemployment to sufficiently employed rate | 1.477396 | | | The initial value of 1.407043821, sourced from Wilkins [75], was calibrated using constrained optimisation. |  |
| Post-secondary qualification probability ratio underemployed vs population | 0.905970 | | | The initial value of 0.86282872, sourced from Wilkins [75], was calibrated using constrained optimisation. |  |
| Effect of moderate to very high distress on employment | 0.881643 | | | The initial value of 0.8396596, sourced from Frijters et al [19], was calibrated using constrained optimisation. |  |
| Moderate distress prevalence ratio unemployed by age 15-24, 25+ | 15-24 years | | 1.329397 | The initial value of 1.399365, sourced from the Australian Bureau of Statistics [76], was calibrated using constrained optimisation. |  |
|  | 25 years and older | | 1.649402 | The initial value of 1.735702, sourced from the Australian Bureau of Statistics [76], was calibrated using constrained optimisation. |  |
| Moderate distress prevalence ratio participation by age 15-24, 25+ | 15-24 years | | 1.039404 | The initial value of 0.994071, sourced from the Australian Bureau of Statistics [76], was calibrated using constrained optimisation. |  |
|  | 25 years and older | | 0.963598 | The initial value of 0.917712, sourced from the Australian Bureau of Statistics [76], was calibrated using constrained optimisation. |  |
| Effect of post-secondary qualification on participation | 1.364018 | | | The initial value of 1.435808, sourced from the Australian Bureau of Statistics [14], was calibrated using constrained optimisation. |  |
| Post-secondary qualifications prevalence ratio participation vs population | 1.026 | | | The initial value of 1.08, sourced from the Australian Bureau of Statistics [14], was calibrated using constrained optimisation. |  |
| Effect of post-secondary qualification on employment vs low educational attainment | 1.587616 | | | The initial value of 1.512015, sourced from the Australian Bureau of Statistics [14], was calibrated using constrained optimisation. |  |
| Post-secondary qualifications prevalence ratio unemployed vs population | 0.8631 | | | The initial value of 0.822, sourced from the Australian Bureau of Statistics [14], was calibrated using constrained optimisation. |  |
| Effect of secondary qualification only on participation | 1.217237 | | | The initial value of 1.281302, sourced from the Australian Bureau of Statistics [14], was calibrated using constrained optimisation. |  |
| Secondary qualifications only prevalence ratio participation vs population | 0.917274 | | | The initial value of 0.964, sourced from the Australian Bureau of Statistics [14], was calibrated using constrained optimisation. |  |
| Effect of secondary qualification only on employment vs low educational attainment | 1.370686 | | | The initial value of 1.305415, sourced from the Australian Bureau of Statistics [14], was calibrated using constrained optimisation. |  |
| Secondary qualifications only prevalence ratio unemployed vs population | 1.3545 | | | The initial value of 1.29, sourced from the Australian Bureau of Statistics [14], was calibrated using constrained optimisation. |  |
| **NEET** | | | | |  |
| Proportion of population NEET ratio 15-17 / 15-24 | 0.132655 | | | Australian Bureau of Statistics [14] |  |
| Coefficient for secondary students aged 15-24 and not employed initial | 0.414217 | | | Estimated with constrained optimisation |  |
| Coefficient for post-sec students aged 15-24 and not employed initial | 0.435501 | | | Estimated with constrained optimisation |  |
| Proportion of individuals aged 15-24 from secondary students | 0.895735 | | | The initial value of 0.995261, sourced from the Australian Bureau of Statistics [14], was calibrated using constrained optimisation. |  |
| Proportion of individuals aged 15-24 from post-sec students | 0.480362 | | | The initial value of 0.533735, sourced from the Australian Bureau of Statistics [14], was calibrated using constrained optimisation. |  |
| **Psychological distress / disorder** | | | | |  |
| Prevalence of No disorder, moderate to very high distress initial by age 12+ | 12-14 years | | 0.256387 | Estimated with constrained optimisation |  |
|  | 15-17 years | | 0.310907 |  |  |
|  | 18-24 years | | 0.205747 |  |  |
|  | 25 years and older | | 0.165037 |  |  |
| Prevalence of Disorder, moderate to very high distress initial by age 12+ | 12-14 years | | 0.193260 | Estimated with constrained optimisation |  |
|  | 15-17 years | | 0.234356 |  |  |
|  | 18-24 years | | 0.155088 |  |  |
|  | 25 years and older | | 0.124403 |  |  |
| Death rate ratio Moderate distress vs Low distress | 1.167023 | | | The initial value of 1.16, sourced from Russ et al [33], was calibrated using constrained optimisation. |  |
| Death rate ratio High distress vs Low distress | 1.383874 | | | The initial value of 1.37, sourced from Russ et al [33], was calibrated using constrained optimisation. |  |
| Prevalence of low distress ratio Australia vs WS by age 12+ | 12-14 years | | 1 | The initial value of (1,1, 1.019869995, 1.019869995) for the age groups respectively, sourced from the Centre for Epidemiology and Evidence [17], was calibrated using constrained optimisation. |  |
|  | 15-17 years | | 1 |  |  |
|  | 18-24 years | | 1.039216 |  |  |
|  | 25 years and older | | 1.013790 |  |  |
| Prevalence of disorder ratio Australia vs WS by age 12+ | 12-14 years | | 1 | The initial value of (1,1, 0.964521433, 0.964521433) for the age groups respectively, sourced from the Centre for Epidemiology and Evidence [17], was calibrated using constrained optimisation. |  |
|  | 15-17 years | | 1 |  |  |
|  | 18-24 years | | 0.938716 |  |  |
|  | 25 years and older | | 0.959665 |  |  |
| Effect of unemployment on distress | 15-24 years | | 1.455968 | The initial value of 1.448417, sourced from the Australian Bureau of Statistics [76], was calibrated using constrained optimisation. |  |
|  | 25 years and older | | 1.764232 | The initial value of 1.761353, sourced from the Australian Bureau of Statistics [76], was calibrated using constrained optimisation. |  |
| Unemployment rate ratio of low distress vs population by age 15-24, 25+ | 15-24 years | | 0.725174 | The initial value of 0.727173, sourced from the Australian Bureau of Statistics [76], was calibrated using constrained optimisation. |  |
|  | 25 years and older | | 0.671368 | The initial value of 0.671579, sourced from the Australian Bureau of Statistics [76], was calibrated using constrained optimisation. |  |
| Effect of homelessness on distress | 2.153408 | | | The initial value of 2.14, sourced from the Australian Bureau of Statistics [76], was calibrated using constrained optimisation. |  |
| Homelessness prevalence ratio of low distress vs population | 0.569590 | | | The initial value of 0.567, sourced from the Australian Bureau of Statistics [76], was calibrated using constrained optimisation. |  |
| Effect of underemployment on distress | 1.143099 | | | The initial value of 1.132448, sourced from Dooley et al [26], was calibrated using constrained optimisation. |  |
| Underemployment ratio of low distress vs population | 0.997446 | | | The initial value of 1.003201, sourced from Griffiths et al [27], was calibrated using constrained optimisation. |  |
| Effect of substance abuse on distress | 2.629305 | | | The initial value of 2.63, sourced from Marmorstein et al [28], was calibrated using constrained optimisation. |  |
| Substance misuse prevalence ratio of low distress vs population | 0.661688 | | | The initial value of 0.6595638, sourced from the Australian Bureau of Statistics [77], was calibrated using constrained optimisation. |  |
| Effect of FDV on distress | 1.575948 | | | The initial value of 1.5575, sourced from the Australian Bureau of Statistics [77], was calibrated using constrained optimisation. |  |
| FDV prevalence ratio of low distress vs population | 0.672985 | | | The initial value of 0.672138, sourced from the Australian Bureau of Statistics [77], was calibrated using constrained optimisation. |  |
| Effect of high vulnerability in early life on distress | 1.177792 | | | The initial value of 1.16, sourced from the Australian Bureau of Statistics [77], was calibrated using constrained optimisation. |  |
| High vulnerability in early life prevalence ratio of low distress vs population | 0.593812 | | | Estimated with constrained optimisation |  |
| Effect of being involved in a legal proceeding on distress | 1.869886 | | | The initial value of 1.854634, sourced from the Australian Bureau of Statistics [77], was calibrated using constrained optimisation. |  |
| Involved in a legal proceeding ratio of low distress vs population | 0.532481 | | | The initial value of 0.536124, sourced from the Australian Bureau of Statistics [77], was calibrated using constrained optimisation. |  |
| Distress recovery per year base rate by age 12+ | 12-14 years | | 0.068955 | The initial value of 0.06833333, sourced from Jokela et al [78], was calibrated using constrained optimisation. |  |
|  | 15-17 years | | 0.068635 |  |  |
|  | 18-24 years | | 0.067603 |  |  |
|  | 25 years and older | | 0.068129 |  |  |
| Disorder recovery per year base rate by age 12+ | 12-14 years | | 0.068097 |  |  |
|  | 15-17 years | | 0.067978 |  |  |
|  | 18-24 years | | 0.068411 |  |  |
|  | 25 years and older | | 0.067583 |  |  |
| Proportion of disorder recovery to no disorder, moderate to very high distress through treatment | 0.8 | | | Assumes that 80% of people with a mental disorder who recover through accessing services will recover to a state of moderate to very high psychological distress and that 20% will recover into a state of low psychological distress. |  |
| Proportion of Close to average SDQ to Low distress | 0.519595 | | | The initial value of 0.468, sourced from the Australian Institute of Family Studies [36], was calibrated using constrained optimisation. |  |
| Proportion of Close to average SDQ to Disorder, Moderate to Very high distress | 0.119296 | | | The initial value of 0.238, sourced from the Australian Institute of Family Studies [36], was calibrated using constrained optimisation. |  |
| Proportion of Slightly raised SDQ to Low distress | 0.196674 | | | The initial value of 0.182, sourced from the Australian Institute of Family Studies [36], was calibrated using constrained optimisation. |  |
| Proportion of Slightly raised SDQ to Disorder, Moderate to Very high distress | 0.767325 | | | The initial value of 0.62, sourced from the Australian Institute of Family Studies [36], was calibrated using constrained optimisation. |  |
| Proportion of High SDQ to Low distress | 0.129127 | | | The initial value of 0.112, sourced from the Australian Institute of Family Studies [36], was calibrated using constrained optimisation. |  |
| Proportion of High SDQ to Disorder, Moderate to Very high distress | 0.580884 | | | The initial value of 0.72, sourced from the Australian Institute of Family Studies [36], was calibrated using constrained optimisation. |  |
| **Strengths and Difficulties** | | | | |  |
| Proportion of High SDQ recovery to Slightly raised through treatment | 0.8 | | | Assumes that 80% of people with High levels of SDQ who recover through accessing services will recover to a state of Slightly raised SDQ and that 20% will recover into a state of Close to average SDQ |  |
| Prevalence of slightly raised SDQ initial by age <12 | 0-4 years | | 0.132000 | Estimated with constrained optimisation |  |
|  | 5-11 years | | 0.096700 |  |  |
| Prevalence of high SDQ initial by age <12 | 0-4 years | | 0.023633 |  |  |
|  | 5-11 years | | 0.094587 |  |  |
| Prevalence of slightly raised SDQ ratio rest of Australia vs WSPHN by age <12 | 0-4 years | | 0.931961 | Australian Institute of Family Studies [36] |  |
|  | 5-11 years | | 1.019318 |  |  |
| Prevalence of high SDQ ratio rest of Australia vs WSPHN by age <12 | 0-4 years | | 2.709695 |  |  |
|  | 5-11 years | | 1.021291 |  |  |
| Slightly raised to Close to average SDQ per year base rate | 0.06833333 | | | Jokela et al [78] |  |
| High to Slightly raised SDQ per year base rate | 0.06833333 | | |  |  |
| Effect of being a victim  of FDV on worsening SDQ | 4.77 | | | Gartland et al [79] |  |
| Victim of FDV prevalence ratio of close to average SDQ vs population | 0.955 | | | Estimated with constrained optimisation |  |
| Victim of FDV prevalence ratio of slightly raised SDQ vs population | 1.37 | | | Estimated with constrained optimisation |  |
| **Homelessness** | | | | |  |
| Homeless by age initial | 0–4 years | | 244.636244 | Estimated with constrained optimisation |  |
|  | 5–11 years | | 269.707421 |  |  |
|  | 12-14 years | | 98.866731 |  |  |
|  | 15-17 years | | 119.439523 |  |  |
|  | 18-24 years | | 765.984931 |  |  |
|  | 25 years and older | | 2146.458927 |  |  |
| Entering homelessness per week base rate by age | 0–4 years | | 0.000048 | Estimated with constrained optimisation |  |
|  | 5–11 years | | 0.000022 |  |  |
|  | 12-14 years | | 0.000015 |  |  |
|  | 15-17 years | | 0.000021 |  |  |
|  | 18-24 years | | 0.000051 |  |  |
|  | 25 years and older | | 0.000021 |  |  |
| Mean duration of homelessness | 183.309940 | | | The initial value of 239.8571, sourced from Ranney [39], was calibrated using constrained optimisation. |  |
| Death rate ratio homeless vs non-homeless | 1.641597 | | | The initial value of 1.6, sourced from Morrison [37], was calibrated using constrained optimisation. |  |
| Effect of unemployment on entering homelessness | 2.6 | | | Nilsson et al [38] |  |
| Effect of mental illness on entering homelessness | 1.7 | | |  |  |
| Effect of substance misuse on entering homelessness | 2.3 | | |  |  |
| **Substance misuse** | | | | |  |
| Waiting for substance misuse treatment by age 15-24, 25+ initial | 15-24 years | | 262.275686 | Estimated with constrained optimisation |  |
|  | 25 years and older | | 271.325678 |  |  |
| Substance misuse seeking treatment per capita per week rate initial | 15-24 years | | 0.001104 | Estimated with constrained optimisation |  |
|  | 25 years and older | | 0.001814 |  |  |
| Substance misuse seeking treatment per capita per week increase per year | 15-24 years | | 0.000096 | Estimated with constrained optimisation |  |
|  | 25 years and older | | 0.000216 |  |  |
| Substance misuse onset per capita per week base rate by age 15-24, 25+ | 15-24 years | | 0.000093 | Estimated with constrained optimisation |  |
|  | 25 years and older | | 0.000030 |  |  |
| Substance misuse natural recovery per capita per week base rate by age 15-24, 25+ | 15-24 years | | 0.000983 | Estimated with constrained optimisation |  |
|  | 25 years and older | | 0.002900 |  |  |
| Substance misuse treatment capacity per week initial | 36.199683 | | | Estimated with constrained optimisation |  |
| Substance misuse treatment capacity per week increase per year | 3.932949 | | | Estimated with constrained optimisation |  |
| Substance misuse treatment mean duration | 15.993141 | | | The initial value sourced from WSPHN provided data and the Australian Institute of Health and Welfare [59], was calibrated using constrained optimisation. |  |
| Prevalence of substance misuse ratio rest of Australia vs WSPHN by age 15-24, 25+ | 15-24 years | | 1.050000 | Estimated with constrained optimisation |  |
|  |  |  |  |  |  |
|  | 15-24 years | | 1.049299 |  |  |
| Death rate ratio substance misuse vs no substance misuse | 1.410792 | | | The initial value of 1.95, sourced from Roerecke and Rehm [55], was calibrated using constrained optimisation. |  |
| Effect of homelessness on substance misuse | 1.413611 | | | The initial value of 1.65, sourced from Johnson et al [57], was calibrated using constrained optimisation. |  |
| Prevalence of homelessness in the non substance misuse population vs total population | 0.676519 | | | The initial value of 0.8683555, sourced from Slade et al [80], was calibrated using constrained optimisation. |  |
| Effect of moderate to very high distress on substance misuse | 2.794132 | | | The initial value of 2.505036, sourced from the Australian Institute of Health and Welfare [56], was calibrated using constrained optimisation. |  |
| Prevalence of distress in the non substance misuse population vs total population | 1.112834 | | | The initial value of 0.9916363, sourced from the Australian Institute of Health and Welfare [56], was calibrated using constrained optimisation. |  |
| Effect of NEET substance misuse | 1.376664 | | | The initial value of 1.43, sourced from Gariépy et al [58], was calibrated using constrained optimisation. |  |
| Prevalence of NEET in the non substance misuse population vs total population | 0.863198 | | | The initial value of 1.006427, sourced from the Australian Bureau of Statistics [25], was calibrated using constrained optimisation. |  |
| Average number of closed treatment episodes per client | 1.521412 | | | The initial value of 1.7 for NSW, sourced from the Australian Institute of Health and Welfare [59], was calibrated using constrained optimisation. |  |
| Recovery rate from substance misuse treatment | 0.353564 | | | The initial value of 0.359, sourced from Manning et al [81], was calibrated using constrained optimisation. |  |
| **Suicidal behaviours** | | | | |  |
| Suicide attempt lethality | 12-14 years | | 0.034692 | Estimated with constrained optimisation |  |
|  | 15-17 years | | 0.026165 |  |  |
|  | 18-24 years | | 0.057305 |  |  |
|  | 25 years and older | | 0.144701 |  |  |
| Index suicide attempt base rate by age 12+ | 12-14 years | | 0.000004 | Estimated with constrained optimisation |  |
|  | 15-17 years | | 0.000017 |  |  |
|  | 18-24 years | | 0.000013 |  |  |
|  | 25 years and older | | 0.000008 |  |  |
| Repeat suicide attempts per week base rate | 0.003425 | | | Estimated with constrained optimisation |  |
| Suicide rate ratio substance misuse disorder vs no substance misuse disorder | 2.87 | | | The initial value of 4.1, sourced from Too et al [61], was calibrated using constrained optimisation. |  |
| Suicide attempt rate ratio by distress | No distress | | 1 (Reference) | The initial values of (1,1.41,3.57) for (No distress, Distress No Disorder, Distress Disorder), sourced from Hockey et al [60], was calibrated using constrained optimisation. |  |
|  | Distress No Disorder | | 1.128 |  |  |
|  | Distress Disorder | | 2.142 |  |  |
| **Mental health services** | | | | |  |
| Effect of disengagement on increasing psychological distress | 1.326486 | | | The initial value of 1.271517, sourced from the Australian Bureau of Statistics [77], was calibrated using constrained optimisation. |  |
| Baseline disengagement rate waiting per year | 0.288231 | | | The initial value of 0.2620284, sourced from Tyrer et al [82], was calibrated using constrained optimisation. |  |
| Baseline disengagement rate hospital care | 0.007429 | | | The initial value of 0.006896, sourced from the Centre for Epidemiology and Evidence [83], was calibrated using constrained optimisation. |  |
| Baseline disengagement rate non-hospital care | 0.043007 | | | The initial value of 0.03909747, sourced from the Australian Institute of Health and Welfare [84], was calibrated using constrained optimisation. |  |
| Effect of distress on hospitalisation | No distress | | 0 | Australian Bureau of Statistics [71] |  |
|  | Distress No Disorder | | 1 (Reference) |  |  |
|  | Distress Disorder | | 1.773723 |  |  |
| Mean treatment duration psychiatric hospital care | 3.3 | | | Australian Institute of Health and Welfare [85] |  |
| Mean treatment duration non-specialised hospital care | 0.957143 | | | Australian Institute of Health and Welfare [85] |  |
| Mean treatment duration online services | 6 | | | Christensen et al [86] |  |
| Seeking help to online services rate by age | 0–4 years | | 0.047651 | Australian Bureau of Statistics [25] |  |
|  | 5–11 years | | 0.047651 |  |  |
|  | 12-14 years | | 0.082294 |  |  |
|  | 15-17 years | | 0.082294 |  |  |
|  | 18-24 years | | 0.082294 |  |  |
|  | 25 years and older | | 0.031692 |  |  |
| Referral rate from GP to headspace | 0–4 years | | 0 | The initial value of (0,0, 0.00199, 0.00199, 0.00199, 0.00199) for the age groups respectively, sourced from the WSPHN provided data and the Australian Institute of Health and Welfare [66], was calibrated using constrained optimisation. |  |
|  | 5–11 years | | 0 |  |  |
|  | 12-14 years | | 0.002031 |  |  |
|  | 15-17 years | | 0.001900 |  |  |
|  | 18-24 years | | 0.001671 |  |  |
|  | 25 years and older | | 0.001460 |  |  |
| Referral rate from GP to other services | 0.046749 | | | The initial value sourced from the Australian Institute of Health and Welfare [87], and calibrated using constrained optimisation. |  |
| Effect of distress on help-seeking with GP | No distress | | 0 | The initial value of (0,1, 3.287037) for the distress levels respectively, sourced from the Australian Bureau of Statistics [71], was calibrated using constrained optimisation. |  |
|  | Distress No Disorder | | 0.9 |  |  |
|  | Distress Disorder | | 2.958333 |  |  |
| Proportion of post-discharge referrals to non-CMHC services to GP | 0.5 | | | Assumes half of patients not referred to CMHC services after discharge from hospital care are referred to a general practitioner. The remaining patients (i.e., those not referred to CMHC services or a general practitioner) are referred to a psychiatrist or allied mental health professional |  |
| Effect of distress on referral rate | No distress | | 0 | Australian Bureau of Statistics [71] |  |
|  | Distress No Disorder | | 1 |  |  |
|  | Distress Disorder | | 1.786096 |  |  |
| Effect of distress on seeking help psychiatrist or allied health services | No distress | | 0 | Australian Bureau of Statistics [71] |  |
|  | Distress No Disorder | | 1 |  |  |
|  | Distress Disorder | | 4.398422 |  |  |
| Baseline recovery rate psychiatric hospital care | No distress | | 0 | Thase et al [88] |  |
|  | Distress No Disorder | | 0.4241071 |  |  |
|  | Distress Disorder | | 0.3712737 |  |  |
| Baseline recovery rate headspace | 0.050295858 | | | KPMG [89] |  |
| Baseline recovery rate CMHC services | 0.02332282 | | | Australian Institute of Health and Welfare [90] |  |
| Recovery rate ratio GP services | No distress | | 0 | Cuijpers et al [91] |  |
|  | Distress No Disorder | | 1 |  |  |
|  | Distress Disorder | | 0.4626866 |  |  |
| Baseline recovery rate mental health treatment | No distress | | 0 | Thase et al [88] |  |
|  | Distress No Disorder | | 0.09525994 |  |  |
|  | Distress Disorder | | 0.08339287 |  |  |
| Psychological treatment rate GP services | 0.48343875 | | | Australian Institute of Health and Welfare [90] |  |
| Recovery rate online services | No distress | | 0 | Christensen et al [86] and Cuijpers et al [92] |  |
|  | Distress No Disorder | | 0.4 |  |  |
|  | Distress Disorder | | 0.1850746 |  |  |
| Perceived needs for services per capita per week rate initial | No distress | | 0 (all age groups) | Estimated with constrained optimisation |  |
|  | Distress No Disorder | 0–4 years | 0.003272 |  |  |
|  |  | 5–11 years | 0.004060 |  |  |
|  |  | 12-14 years | 0.001455 |  |  |
|  |  | 15-17 years | 0.001319 |  |  |
|  |  | 18-24 years | 0.002434 |  |  |
|  |  | 25 years and older | 0.002132 |  |  |
|  | Distress Disorder | 0–4 years | 0.076895 |  |  |
|  |  | 5–11 years | 0.023252 |  |  |
|  |  | 12-14 years | 0.009915 |  |  |
|  |  | 15-17 years | 0.008948 |  |  |
|  |  | 18-24 years | 0.016401 |  |  |
|  |  | 25 years and older | 0.016951 |  |  |
| Perceived needs for services per capita per week rate initial | No distress | | 0 (all age groups) | Estimated with constrained optimisation |  |
|  | Distress No Disorder | 0–4 years | 0.000095 |  |  |
|  |  | 5–11 years | 0.000206 |  |  |
|  |  | 12-14 years | 0.000021 |  |  |
|  |  | 15-17 years | 0.000041 |  |  |
|  |  | 18-24 years | 0.0000002 |  |  |
|  |  | 25 years and older | 0.0000008 |  |  |
|  | Distress Disorder | 0–4 years | 0.012784 |  |  |
|  |  | 5–11 years | 0.003867 |  |  |
|  |  | 12-14 years | 0.000107 |  |  |
|  |  | 15-17 years | 0.000212 |  |  |
|  |  | 18-24 years | 0.000014 |  |  |
|  |  | 25 years and older | 0.000051 |  |  |
| Re-engaging excluding ED per capita per week rate initial | No distress | | 0 | Estimated with constrained optimisation |  |
|  | Distress No Disorder | | 0.003341 |  |  |
|  | Distress Disorder | | 0.118805 |  |  |
| Re-engaging excluding ED per capita per week rate increase per year | No distress | | 0 | Estimated with constrained optimisation |  |
|  | Distress No Disorder | | -0.000003 |  |  |
|  | Distress Disorder | | -0.003724 |  |  |
| Proportion of population in distress seeking help initial by distress | No distress | | 0 | Estimated with constrained optimisation |  |
|  | Distress No Disorder | | 0.184351 |  |  |
|  | Distress Disorder | | 0.803402 |  |  |
| Disengaged initial total | 0 | | | Estimated with constrained optimisation |  |
| GP services capacity per week initial | 1819.705233 | | | Estimated with constrained optimisation against the data from the Australian Institute of Health and Welfare [66] and the Department of Health [67]. |  |
| GP services capacity per week increase per year | 82.176689 | | |  |  |
| Psychiatrist and allied health services capacity per week initial | 3711.870822 | | |  |  |
| Psychiatrist and allied health services capacity per week increase per year | 225.934812 | | |  |  |
| CYMHS services capacity per week initial | 380.396649 | | | Estimated with constrained optimisation against the data from the Australian Institute of Health and Welfare [68] |  |
| CYMHS services capacity per week increase per year | 7.631373 | | |  |  |
| CMHC services (adult) capacity per week initial | 2863.109439 | | |  |  |
| CMHC services (adult) capacity per week increase per year | 61.500816 | | |  |  |
| Psychiatric hospitalisation capacity per week initial | 110.767341 | | | Estimated with constrained optimisation against the data from the Centre for Epidemiology and Evidence [70,73]. |  |
| Psychiatric hospitalisation capacity per week increase per year | 8.209896 | | |  |  |
| Non-specialised hospital care services capacity per week initial | 102.379810 | | |  |  |
| Non-specialised hospital care services capacity capacity per week increase per year | 4.414076 | | |  |  |
| headspace services capacity per week initial | 616.792050 | | | Estimated with constrained optimisation against the data from WSPHN. |  |
| headspace services capacity capacity per week increase per year | 108.963901 | | |  |  |
| ED presentations per capita per week rate by age initial | 0–4 years | | 0.0000100 | Estimated with constrained optimisation |  |
|  | 5–11 years | | 0.0000275 |  |  |
|  | 12-14 years | | 0.0002249 |  |  |
|  | 15-17 years | | 0.0002159 |  |  |
|  | 18-24 years | | 0.0003062 |  |  |
|  | 25 years and older | | 0.0002476 |  |  |
| ED presentations rate by age increase per year | 0–4 years | | 0.0000035 |  |  |
|  | 5–11 years | | 0.0000139 |  |  |
|  | 12-14 years | | 0.0000043 |  |  |
|  | 15-17 years | | 0.0000064 |  |  |
|  | 18-24 years | | -0.0000039 |  |  |
|  | 25 years and older | | -0.0000036 |  |  |
| ED presentation rate ratio help seeker | 2.8565384 | | | Estimated with constrained optimisation |  |
| ED to admission rate initial by age | 0–4 years | | 0.308980 | Estimated with constrained optimisation |  |
|  | 5–11 years | | 0.374244 |  |  |
|  | 12-14 years | | 0.339171 |  |  |
|  | 15-17 years | | 0.381382 |  |  |
|  | 18-24 years | | 0.428756 |  |  |
|  | 25 years and older | | 0.445572 |  |  |
| ED to admission rate increase per year | 0–4 years | | -0.012275 |  |  |
|  | 5–11 years | | -0.013189 |  |  |
|  | 12-14 years | | -0.00368814 |  |  |
|  | 15-17 years | | -0.004900 |  |  |
|  | 18-24 years | | -0.0047605 |  |  |
|  | 25 years and older | | -0.00404451 |  |  |
| Proportion of admissions into psychiatric hospitalisation by age | 0–4 years | | 0.617744 | Estimated with constrained optimisation |  |
|  | 5–11 years | | 0.952092 |  |  |
|  | 12-14 years | | 0.394692 |  |  |
|  | 15-17 years | | 0.676716 |  |  |
|  | 18-24 years | | 0.873577 |  |  |
|  | 25 years and older | | 0.225081 |  |  |
| Additional non-specialised hospitalisations per capita per week by age | 0–4 years | | 0.000069 | Estimated with constrained optimisation |  |
|  | 5–11 years | | 0.000062 |  |  |
|  | 12-14 years | | 0.000000 |  |  |
|  | 15-17 years | | 0.000346 |  |  |
|  | 18-24 years | | 0.000470 |  |  |
|  | 25 years and older | | 0.000537 |  |  |
| Referral rate from ED to CMHC | 0.061713 | | | Estimated with constrained optimisation |  |
| Waiting for psychiatric hospitalisation initial total | 20 | | | Estimated with constrained optimisation |  |
| Seeking help GP services rate by age 0-14, 15-24, 25+ initial | 0-14 years | | 0.002744 | Estimated with constrained optimisation |  |
|  | 15-24 years | | 0.003618 |  |  |
|  | 25 years and older | | 0.009076 |  |  |
| Seeking help GP services rate by age 0-14, 15-24, 25+ increase per year | 0-14 years | | 0.000136 | Estimated with constrained optimisation |  |
|  | 15-24 years | | 0.000390 |  |  |
|  | 25 years and older | | -0.000120 |  |  |
| Waiting for GP initial total | 42.946369 | | | Estimated with constrained optimisation |  |
| Referral rate from GP to psychiatrist and allied health service initial | 0.044998 | | | Estimated with constrained optimisation |  |
| Referral rate from GP to psychiatrist and allied health service increase per year | 0.005319 | | | Estimated with constrained optimisation |  |
| Referral rate from GP to CMHC | 0.001878 | | | Estimated with constrained optimisation |  |
| Additional psychiatrist or allied health services per capita per week initial by age 0-14, 15-24, 25+ | 0-14 years | | 0.009783 | Estimated with constrained optimisation |  |
|  | 15-24 years | | 0.006519 |  |  |
|  | 25 years and older | | 0.018245 |  |  |
| Additional psychiatrist or allied health services per capita per week increase per year by age 0-14, 15-24, 25+ | 0-14 years | | 0.000627 | Estimated with constrained optimisation |  |
|  | 15-24 years | | 0.004804 |  |  |
|  | 25 years and older | | 0.000128 |  |  |
| Referral rate from psychiatrist and allied health to psychiatric hospital care services by age 0-17, 18-24, 25+ | 0–4 years | | 0.002000 | Estimated with constrained optimisation |  |
|  | 5–11 years | | 0.002000 |  |  |
|  | 12-14 years | | 1.00E-06 |  |  |
|  | 15-17 years | | 0.006900 |  |  |
|  | 18-24 years | | 0.0075 |  |  |
|  | 25 years and older | | 0.0244 |  |  |
| Waiting for psychiatrist and allied health initial total | 24632.48458 | | | Estimated with constrained optimisation |  |
| Referral rate from post-discharge to CMHC services initial | 0–4 years | | 0.605707 | Estimated with constrained optimisation |  |
|  | 5–11 years | | 0.602860 |  |  |
|  | 12-14 years | | 0.587488 |  |  |
|  | 15-17 years | | 0.577826 |  |  |
|  | 18-24 years | | 0.578321 |  |  |
|  | 25 years and older | | 0.624130 |  |  |
| Referral rate from post-discharge to CMHC services increase per year | 0–4 years | | 0.016129 |  |  |
|  | 5–11 years | | 0.016129 |  |  |
|  | 12-14 years | | 0.016129 |  |  |
|  | 15-17 years | | 0.016129 |  |  |
|  | 18-24 years | | 0.016129 |  |  |
|  | 25 years and older | | 0.014167 |  |  |
| Additional CMHC per capita per week initial by age AIHW | 0–4 years | | 0.002129 | Estimated with constrained optimisation |  |
|  | 5–11 years | | 0.012153 |  |  |
|  | 12-17 years | | 0.019376 |  |  |
|  | 18-24 years | | 0.013936 |  |  |
|  | 25 years and older | | 0.019902 |  |  |
| Additional CMHC per capita per week increase per year by age AIHW | 0–4 years | | -0.000050 |  |  |
|  | 5–11 years | | -0.000050 |  |  |
|  | 12-17 years | | -0.000001 |  |  |
|  | 18-24 years | | 0.000400 |  |  |
|  | 25 years and older | | 0.000300 |  |  |
| Waiting for CMHC initial total | 376.090464 | | | Estimated with constrained optimisation |  |
| Seeking help headspace rate by age 12+ initial | 12-14 years | | 0.000001 | Estimated with constrained optimisation |  |
|  | 15-17 years | | 0.000000 |  |  |
|  | 18-24 years | | 0.005297 |  |  |
|  | 25 years and older | | 0.000194 |  |  |
| Seeking help headspace rate by age 12+ increase per year | 12-14 years | | 0.001764 |  |  |
|  | 15-17 years | | 0.003481 |  |  |
|  | 18-24 years | | 0.001367 |  |  |
|  | 25 years and older | | -0.000011 |  |  |
| Waiting for headspace initial total | 0 | | | Estimated with constrained optimisation |  |

# **References**

1. Department of Health and Aged Care. Primary Health Networks (PHNs) collection of concordance files. 2024. Available from: https://www.health.gov.au/resources/collections/primary-health-networks-phns-collection-of-concordance-files (accessed Feb 10, 2024).

2. Centre for Epidemiology and Evidence. HealthStats, NSW Ministry of Health: Death rates by Primary Health Networks in NSW. 2022. Available from: https://www.healthstats.nsw.gov.au/ (accessed Feb 10, 2024).

3. Centre for Epidemiology and Evidence. HealthStats, NSW Ministry of Health: Population estimates Primary Health Networks NSW. 2024. Available from: https://www.healthstats.nsw.gov.au/r/114532 (accessed Feb 10, 2024).

4. Centre for Epidemiology and Evidence. HealthStats, NSW Ministry of Health: Births by Primary Health Networks in NSW. 2022. Available from: https://www.healthstats.nsw.gov.au (accessed Feb 10, 2024).

5. PHIDU. Torrens University Australia: Social Health Atlas of Australia (Data by Primary Health Network). 2024. Available from: https://phidu.torrens.edu.au/social-health-atlases/data#social-health-atlas-of-australia-population-health-areas (accessed Feb 10, 2024).

6. Australian Bureau of Statistics. Data Explorer. Interstate migration: Arrivals, departures and net, State/territory, Age and sex - 1997 onwards. 2023. Available from: https://explore.data.abs.gov.au/ (accessed Feb 10, 2024).

7. Australian Bureau of Statistics. Data Explorer. Net overseas migration, Arrivals, departures and net, State/territory, Age and sex - 2004 onwards. 2023. Available from: https://explore.data.abs.gov.au/ (accessed Feb 10, 2024).

8. Australian Bureau of Statistics. Data Explorer. Net internal and overseas migration estimates by region (SA2 and above) and age, 2016-17 onwards. 2023. Available from: https://explore.data.abs.gov.au/ (accessed Feb 10, 2024).

9. Butterworth P, Leach LS. Early Onset of Distress Disorders and High-School Dropout: Prospective Evidence from a National Cohort of Australian Adolescents. Am J Epidemiol; 2018;187(6):1192–8. doi: 10.1093/aje/kwx353

10. Lee S, Tsang A, Breslau J, Aguilar-Gaxiola S, Angermeyer M, Borges G, et al. Mental disorders and termination of education in high-income and low- and middle-income countries: epidemiological study. Br J Psychiatry; 2009;194(5):411–7. doi: 10.1192/bjp.bp.108.054841

11. Australian Curriculum Assessment and Reporting Authority. Enrolments by Grade 2008-2022. 2023. Available from: https://acara.edu.au/contact-us/acara-data-access (accessed Feb 10, 2024).

12. Australian Curriculum Assessment and Reporting Authority. Senior Secondary Outcomes 2008-2021. 2023. Available from: https://acara.edu.au/contact-us/acara-data-access (accessed Feb 10, 2024).

13. Centre for Education Statistics and Evaluation. NSW Department of Education. NSW Post-School Destinations and Experiences Surveys. 2023. Available from: https://education.nsw.gov.au/about-us/education-data-and-research/cese/publications/research-reports/nsw-post-school-destinations-and-experiences-survey (accessed Feb 10, 2024).

14. Australian Bureau of Statistics. TableBuilder. Education and Work (2014-2022). 2023. Available from: https://www.abs.gov.au/statistics/microdata-tablebuilder/tablebuilder (accessed Feb 10, 2024).

15. Welsh J, Joshy G, Moran L, Soga K, Law H Di, Butler D, et al. Education-related inequalities in cause-specific mortality: first estimates for Australia using individual-level linked census and mortality data. Int J Epidemiol; 2022;50(6):1981–94. doi: 10.1093/ije/dyab080

16. Sorlie PD, Rogot E. Mortality by employment status in the National Longitudinal Mortality Study. Am J Epidemiol; 1990;132(5):983–92. doi: 10.1093/oxfordjournals.aje.a115741

17. Centre for Epidemiology and Evidence. HealthStats, NSW Ministry of Health: Psychological distress levels in adults by Psychological distress and Age. 2022. Available from: https://www.healthstats.nsw.gov.au/ (accessed Feb 10, 2024).

18. Centre for Epidemiology and Evidence. HealthStats, NSW Ministry of Health: Psychological distress levels in adults by Psychological distress and PHN. 2022. Available from: https://www.healthstats.nsw.gov.au (accessed Feb 10, 2024).

19. Frijters P, Johnston DW, Shields MA. The effect of mental health on employment: evidence from Australian panel data. Health Econ; 2014;23(9):1058–71. Available from: doi: 10.1002/hec.3083

20. Wilkins R, Creedy J, Hsu E, Kalb G, Morrison T, Tseng YP, et al. The Extent and Consequences of Underemployment in Australia. Melbourne Institute of Applied Economic and Social Research, The University of Melbourne, Melbourne Institute Working Paper Series. 2004. Available from: http://www.melbourneinstitute.com

21. Australian Bureau of Statistics. Labour Force, Australia, Detailed. 2023. Available from: https://www.abs.gov.au/statistics/labour/employment-and-unemployment/labour-force-australia-detailed (accessed Feb 10, 2024).

22. Australian Bureau of Statistics. Labour Force, Australia. 2023. Available from: https://www.abs.gov.au/statistics/labour/employment-and-unemployment/labour-force-australia (accessed Feb 10, 2024).

23. Kessler RC, Andrews G, Colpe LJ, Hiripi E, Mroczek DK, Normand SLT, et al. Short screening scales to monitor population prevalences and trends in non-specific psychological distress. Psychol Med; 2002; 32(6): 959–76. doi: 10.1017/s0033291702006074

24. Australian Bureau of Statistics. Estimating Homelessness: Census. 2021. Available from: https://www.abs.gov.au/statistics/people/housing/estimating-homelessness-census (accessed Feb 10, 2024).

25. Australian Bureau of Statistics. National Study of Mental Health and Wellbeing, 2020-2022. 2023. Available from: https://www.abs.gov.au/statistics/health/mental-health/national-study-mental-health-and-wellbeing/latest-release (accessed Feb 10, 2024).

26. Dooley D, Prause J, Ham-Rowbottom KA. Underemployment and depression: Longitudinal relationships. J Health Soc Behav; 2000;41(4):421–36.

27. Griffiths D, Sheehan L, van Vreden C, Petrie D, Grant G, Whiteford P, et al. The Impact of Work Loss on Mental and Physical Health During the COVID-19 Pandemic: Baseline Findings from a Prospective Cohort Study. J Occup Rehabil; 2021;31(3):455–62. doi: 10.1007/s10926-021-09958-7

28. Marmorstein NR, Iacono WG, Malone SM. Longitudinal associations between depression and substance dependence from adolescence through early adulthood. Drug Alcohol Depend; 2010;107(2–3):154–60. doi: 10.1016/j.drugalcdep.2009.10.002

29. Jirapramukpitak T, Harpham T, Prince M. Family violence and its “adversity package”: A community survey of family violence and adverse mental outcomes among young people. Soc Psychiatry Psychiatr Epidemiol; 2011;46(9):825–31. doi: 10.1007/s00127-010-0252-9

30. Renner LM, Schwab-Reese LM, Coppola EC, Boel-Studt S. The contribution of interpersonal violence victimization types to psychological distress among youth. Child Abuse Negl; 2020;106:104493.

31. Meurk C, Steele M, Yap L, Jones J, Heffernan E, Davison S, et al. Changing Direction: Mental Health Needs of Justice-Involved Young People in Australia. The Kirby Institute, The University of New South Wales Sydney. 2019. Available from: https://www.kirby.unsw.edu.au/research/reports/changing-direction-mental-health-needs-justice-involved-young-people-australia

32. Goodman A, Goodman R. Strengths and difficulties questionnaire as a dimensional measure of child mental health. J Am Acad Child Adolesc Psychiatry; 2009;48(4):400–3. doi: 10.1097/CHI.0b013e3181985068

33. Russ TC, Stamatakis E, Hamer M, Starr JM, Kivimäki M, Batty GD. Association between psychological distress and mortality: individual participant pooled analysis of 10 prospective cohort studies. BMJ; 2012;345(7871). doi: 10.1136/bmj.e4933

34. Australian Bureau of Statistics. National Health Survey. 2022. Available from: https://www.abs.gov.au/statistics/health/health-conditions-and-risks/national-health-survey/latest-release (accessed Feb 10, 2024).

35. Young Minds Matter Survey. Survey Results Query Tool. 2014. Available from: http://www.youngmindsmatterresults.org.au/PrevalenceK10BS.html (accessed Feb 10, 2024).

36. Australian Institute of Family Studies. Growing Up in Australia: Longitudinal Study of Australian Children (LSAC) Release 9.1 C2 (Waves 1-9C) [ADA Dataverse]. 2022. Available from: https://growingupinaustralia.gov.au/

37. Morrison DS. Homelessness as an independent risk factor for mortality: results from a retrospective cohort study. Int J Epidemiol; 2009;38(3):877–83. doi: 10.1093/ije/dyp160

38. Nilsson SF, Nordentoft M, Hjorthøj C. Individual-Level Predictors for Becoming Homeless and Exiting Homelessness: a Systematic Review and Meta-analysis. J Urban Health; 2019;96(5):741–50. doi: 10.1007/s11524-019-00377-x

39. Ranney K. Community Solutions. Brisbane Zero reduces the average length of time a person experiences homelessness by 40% with the help of real-time, by-name data. 2023. Available from: https://community.solutions/case-studies/brisbane-zero-reduces-the-average-length-of-time-a-person-experiences-homelessness-by-40-with-the-help-of-real-time-by-name-data/

40. Cox CE, Kotch JB, Everson MD. A Longitudinal Study of Modifying Influences in the Relationship between Domestic Violence and Child Maltreatment. J Fam Violence; 2003;18(1):5–17. doi: 10.1023/A:1021497213505

41. Kyriacou DN, Anglin D, Taliaferro E, Stone S, Tubb T, Linden JA, et al. Risk factors for injury to women from domestic violence. N Engl J Med; 1999;341:1892–8. Available from: https://www.taylorfrancis.com/chapters/edit/10.4324/9781315264905-10. eBook ISBN: 9781315264905

42. Australian Bureau of Statistics. Victims of family and domestic violence-related offences: Recorded Crime - Victims. 2023. Available from: https://www.abs.gov.au/statistics/people/crime-and-justice/recorded-crime-victims (accessed Feb 10, 2024).

43. McReynolds LS, Schwalbe CS, Wasserman GA. The Contribution of Psychiatric Disorder to Juvenile Recidivism. Crim Justice Behav; 2010;37(2):204–16. doi: 10.1177/0093854809354961

44. Wibbelink CJM, Hoeve M, Stams GJJM, Oort FJ. A meta-analysis of the association between mental disorders and juvenile recidivism. Aggress Violent Behav; 2017;33:78–90.

45. Ogilvie JM, Tzoumakis S, Thompson C, Allard T, Dennison S, Kisely S, et al. Psychiatric illness and the risk of reoffending: recurrent event analysis for an Australian birth cohort. BMC Psychiatry; 2023;23(1). doi: 10.1186/s12888-023-04839-0

46. Stoolmiller M, Blechman EA. Substance use is a robust predictor of adolescent recidivism. Crim Justice Behav; 2005;32(3):302–28.

47. Weber M, Baggio S, Gonçalves LC, Nieuwbeerta P, Dirkzwager AJE. Longitudinal trajectories of mental health problems and their association with reoffending in a Dutch pre-trial prison cohort. Front Psychiatry; 2022;13:976832. doi: 10.3389/fpsyt.2022.976832

48. Jacobs LA, Gottlieb A. The Effect of Housing Circumstances on Recidivism: Evidence From a Sample of People on Probation in San Francisco. Crim Justice Behav; 2020;47(9):1097. doi: 10.1177/0093854820942285

49. Astridge B, Li WW, McDermott B, Longhitano C. A systematic review and meta-analysis on adverse childhood experiences: Prevalence in youth offenders and their effects on youth recidivism. Child Abuse Negl; 2023;140:106055.

50. Katsiyannis A, Archwamety T. Factors related to recidivism among delinquent youths in a state correctional facility. J Child Fam Stud; 1997;6(1):43–55. doi: 10.1023/A:1025068623167

51. O’Dea B, Glozier N, Purcell R, McGorry PD, Scott J, Feilds KL, et al. A cross-sectional exploration of the clinical characteristics of disengaged (NEET) young people in primary mental healthcare. BMJ Open; 2014;4(12). doi: 10.1136/bmjopen-2014-006378

52. NSW Bureau of Crime Statistics and Research (BOCSAR). NSW Criminal Courts Statistics. 2022. Available from: https://bocsar.nsw.gov.au/research-evaluations/2024/criminal-court-statistics-dec-2023.html (accessed Feb 10, 2024).

53. NSW Bureau of Crime Statistics and Research (BOCSAR). Youth Offending. 2022. Available from: https://bocsar.nsw.gov.au/topic-areas/young-people.html (accessed Feb 10, 2024).

54. Australian Bureau of Statistics. Recorded Crime - Offenders. 2022. Available from: https://www.abs.gov.au/statistics/people/crime-and-justice/recorded-crime-offenders (accessed Feb 10, 2024).

55. Roerecke M, Rehm J. Alcohol use disorders and mortality: a systematic review and meta-analysis. Addiction; 2013;108(9):1562–78. doi: 10.1111/add.12231

56. Australian Institute of Health and Welfare. ADA Dataverse: National Drug Strategy Household Survey 2019. 2020. doi: 10.26193/WRHDUL

57. Johnson TP, Freels SA, Parsons JA, Vangeest JB. Substance abuse and homelessness: social selection or social adaptation? Addiction; 1997;92(4):437–45. PMID: 9177065

58. Gariépy G, Danna SM, Hawke L, Henderson J, Iyer SN. The mental health of young people who are not in education, employment, or training: a systematic review and meta-analysis. Soc Psychiatry Psychiatr Epidemiol; 2022;57(6):1107–21. doi: 10.1007/s00127-021-02212-8

59. Australian Institute of Health and Welfare. Alcohol & other drug treatment services by Primary Health Network (PHN) geography. 2023. Available from: https://www.aihw.gov.au/reports-data/health-welfare-services/alcohol-other-drug-treatment-services/data (accessed Feb 10, 2024).

60. Hockey M, Rocks T, Ruusunen A, Jacka FN, Huang W, Liao B, et al. Psychological distress as a risk factor for all-cause, chronic disease- and suicide-specific mortality: a prospective analysis using data from the National Health Interview Survey. Soc Psychiatry Psychiatr Epidemiol; 2022;57(3):541–52. doi: 10.1007/s00127-021-02116-7

61. Too LS, Spittal MJ, Bugeja L, Reifels L, Butterworth P, Pirkis J. The association between mental disorders and suicide: A systematic review and meta-analysis of record linkage studies. J Affect Disord; 2019;259:302–13.

62. Australian Institute of Health and Welfare. Suicide and Self-harm Monitoring: National Mortality Database—Suicide (ICD-10 X60–X84, Y87.0). 2022. Available from: https://www.aihw.gov.au/suicide-self-harm-monitoring/data/suicide-self-harm-monitoring-data (accessed Feb 10, 2024).

63. Centre for Epidemiology and Evidence. HealthStats, NSW Ministry of Health: Suicide by PHN. 2022. Available from: https://www.healthstats.nsw.gov.au/r/117083 (accessed Feb 10, 2024).

64. Centre for Epidemiology and Evidence. HealthStats, NSW Ministry of Health: Intentional self-harm hospitalisations by PHN. 2023. Available from: ttps://www.healthstats.nsw.gov.au/r/117084 (accessed Feb 10, 2024).

65. Scanlon Foundation Research Institute. Scanlon-Monash Index. 2023. Available from: https://scanloninstitute.org.au/research/scanlon-monash-index (accessed Feb 10, 2024).

66. Australian Institute of Health and Welfare. AIHW primary health care Data. Data tables: Medicare-subsidised GP, allied health and specialist health care across local areas. 2024. Available from: https://www.aihw.gov.au/reports-data/health-welfare-services/primary-health-care/data (accessed Feb 10, 2024).

67. Department of Health. Primary Mental Health Care Minimum Data Set (PMHC-MDS). 2024. Available from: https://pmhc-mds.com/ (accessed Feb 10, 2024).

68. Australian Institute of Health and Welfare. Community mental health care services. 2018. Available from: https://www.aihw.gov.au/mental-health/topic-areas/community-services (accessed Feb 10, 2024).

69. Centre for Epidemiology and Evidence. HealthStats, NSW Ministry of Health: Mental Health related Emergency Department visits by age and PHN. 2023. Available from: https://www.healthstats.nsw.gov.au/r/111663 (accessed Feb 10, 2024).

70. Centre for Epidemiology and Evidence. HealthStats, NSW Ministry of Health: Hospitalisations by category of cause by PHN. 2024. Available from: https://www.healthstats.nsw.gov.au/r/111647 (accessed Feb 10, 2024).

71. Australian Bureau of Statistics. Microdata: National Study of Mental Health and Wellbeing. 2007. Available from: https://www.abs.gov.au/statistics/microdata-tablebuilder/microdatadownload (accessed Feb 10, 2024).

72. Australian Institute of Health and Welfare. Key Performance Indicators for Australian Public Mental Health Services. 2022. Available from: https://www.aihw.gov.au/mental-health/monitoring/performance-indicators (accessed Feb 10, 2024).

73. Centre for Epidemiology and Evidence. HealthStats, NSW Ministry of Health: Hospitalisations by category of cause. 2024. Available from: https://www.healthstats.nsw.gov.au/r/111647 (accessed Feb 10, 2024).

74. Australian Bureau of Statistics. Education and Work, Australia. 2022. Available from: https://www.abs.gov.au/statistics/people/education/education-and-work-australia (accessed Feb 10, 2024).

75. Wilkins R. The Extent and Consequences of Underemployment in Australia. Melbourne Institute of Applied Economic and Social Research, The University of Melbourne, Melbourne Institute Working Paper NO. 16/04. 2004. Available from: https://melbourneinstitute.unimelb.edu.au/publications/working-papers/search/result?paper=2156141

76. Australian Bureau of Statistics. Microdata: National Health Survey. 2018. Available from: https://www.abs.gov.au/statistics/microdata-tablebuilder/microdatadownload (accessed Feb 10, 2024).

77. Australian Bureau of Statistics. Information Paper: Use of the Kessler Psychological Distress Scale in ABS Health Surveys, Australia, 2007-08. Cat. no. 4817.0.55.001. 2012. Available from: https://www.abs.gov.au/AUSSTATS/abs@.nsf/DetailsPage/4817.0.55.0012007-08?OpenDocument

78. Jokela M, Singh-Manoux A, Shipley MJ, Ferrie JE, Gimeno D, Akbaraly TN, et al. Natural course of recurrent psychological distress in adulthood. J Affect Disord; 2011;130(3):454–61.

79. Gartland D, Woolhouse H, Mensah FK, Hegarty K, Hiscock H, Brown SJ. The Case for Early Intervention to Reduce the Impact of Intimate Partner Abuse on Child Outcomes: Results of an Australian Cohort of First-Time Mothers. Birth; 2014;41(4):374–83. doi: 10.1111/birt.12123

80. Slade T, Johnston A, Teesson M, Whiteford H, Burgess P, Pirkis J, et al. The Mental health of Australians 2: report on the 2007 national survey of mental health and wellbeing. PsycEXTRA Dataset. 2009. doi: 10.1037/e676562010-001

81. Manning V, Garfield JBB, Best D, Berends L, Room R, Mugavin J, et al. Substance use outcomes following treatment: Findings from the Australian Patient Pathways Study. Aust N Z J Psychiatry; 2017;51(2):177–89. doi: 10.1177/0004867415625815

82. Tyrer P, Morgan J, Van Horn E, Jayakody M, Evans K, Brummell R, et al. A randomised controlled study of close monitoring of vulnerable psychiatric patients. The Lancet; 1995;345(8952):756–9.

83. Centre for Epidemiology and Evidence. HealthStats, NSW Ministry of Health: Hospitalisations that end in discharge against medical advice. 2023. Available from: https://www.healthstats.nsw.gov.au/r/111638 (accessed Feb 10, 2024).

84. Australian Institute of Health and Welfare. Mental health: Consumer outcomes in mental health care. 2023. Available from: https://www.aihw.gov.au/mental-health/topic-areas/consumer-outcomes (accessed Feb 10, 2024).

85. Australian Institute of Health and Welfare. Admitted patients - Mental health. 2023. Available from: https://www.aihw.gov.au/mental-health/topic-areas/admitted-patients (accessed Feb 10, 2024).

86. Christensen H, Griffiths KM, Jorm AF. Delivering interventions for depression by using the internet: randomised controlled trial. BMJ; 2004; 328(7434): 265–8. doi: 10.1136/bmj.37945.566632.EE

87. Australian Institute of Health and Welfare. Mental health services in Australia — Mental health-related care in general practice. 2017. Available from: https://www.aihw.gov.au/getmedia/86df866c-05c5-4d44-92c8-d35c3a36f18f/Mental-health-related-service-provided-by-general-practitioners-2015-16.xlsx.aspx (accessed Feb 10, 2024).

88. Thase ME, Greenhouse JB, Frank E, Reynolds CE, Pilkonis PA, Hurley K, et al. Treatment of major depression with psychotherapy or psychotherapy-pharmacotherapy combinations. Arch Gen Psychiatry; 1997;54(11):1009–15. doi: 10.1001/archpsyc.1997.01830230043006

89. KPMG. Evaluation of the National headspace Program. 2022. Available from: https://www.health.gov.au/sites/default/files/documents/2022/10/evaluation-of-the-national-headspace-program.pdf (accessed Feb 10, 2024).

90. Australian Institute of Health and Welfare. Mental health: Mental health services. 2016. Available from: https://www.aihw.gov.au/mental-health/overview/mental-health-services (accessed Feb 10, 2024).

91. Cuijpers P, Smits N, Donker T, ten Have M, de Graaf R. Screening for mood and anxiety disorders with the five-item, the three-item, and the two-item Mental Health Inventory. Psychiatry Res; 2009;168(3):250–5.

92. Cuijpers P, van Straten A, van Schaik A, Andersson G. Psychological treatment of depression in primary care: a meta-analysis. Br J Gen Pract; 2009;59(559):120–7. doi: 10.3399/bjgp09X395139
